# Characterization and evolutionary history of novel SARS-CoV-2-related viruses in bats from Cambodia

**DOI:** 10.1038/s41467-026-75954-1

**Published:** 2026-07-23

**Authors:** Tey Putita Ou, Julia Guillebaud, Artem Baidaliuk, Sothyra Tum, Dany Chheang, Deborah Delaune, Matthieu Prot, Elise Bruder, Rafael Rahal Guaragna Machado, Leakhena Pum, Vibol Hul, Thavry Hoem, Sovann Ly, Heidi Auerswald, Frederick Arnaud, Kei Sato, Gavin JD Smith, Philippe Dussart, Erik A. Karlsson, Véronique Chevalier, Julien Cappelle, Veasna Duong, Etienne Simon-Loriere

**Affiliations:** 1 Virology Unit, Institut Pasteur du Cambodge, Pasteur Network, Phnom Penh, Cambodia; 2 Institut Pasteur, Université Paris Cité, CNRS UMR2000, Evolutionary Genomics of RNA Viruses Unit, Paris, France; 3 Ecole Doctorale GAIA, University of Montpelier, Montpellier, France; 4 Institut Pasteur, Université Paris Cité, Bioinformatics and Biostatistics Hub, Paris, France; 5 General Directorate of Animal Health and Production, Ministry of Agriculture, Forestry and Fisheries, Phnom Penh, Cambodia; 6 Forestry Administration, Ministry of Agriculture, Forestry and Fisheries, Phnom Penh, Cambodia; 7 Communicable Diseases Control Department, Ministry of Health, Phnom Penh, Cambodia; 8 IVPC UMR754, EPHE, Université PSL, INRAE, Université Claude Bernard Lyon 1, Lyon, France; 9 Division of Systems Virology, Department of Microbiology and Immunology, The Institute of Medical Science, The University of Tokyo, Tokyo, Japan; 10 Programme in Emerging Infectious Diseases, Duke-NUS Medical School, Singapore, Singapore; 11 Institut Pasteur de Madagascar, Pasteur Network, Antananarivo, Madagascar; 12 CIRAD, UMR ASTRE, Antananarivo, Madagascar; 13 ASTRE, Université de Montpellier, CIRAD, INRAE, Montpellier, France; 14 CIRAD, UMR ASTRE, Montpellier, France

**Keywords:** SARS-CoV-2, Metagenomics, Viral evolution, Viral reservoirs

## Abstract

Circulating bat coronaviruses present a significant pandemic threat, yet our understanding of their genetic diversity and evolutionary dynamics remains limited. Over 3 years, we sampled 1,462 bats in Cambodia’s Steung Treng province, identifying extensive and diverse coronaviruses co-circulation. Using metatranscriptomic and amplicon sequencing, we generated 33 complete sarbecovirus genomes sequences, revealing novel lineages that cluster into four distinct groups, each associated with different *Rhinolophus* bat species. Our analysis highlights rapid migration and recombination of sarbecovirus lineages over short distances and timescales. Of note, the receptor-binding domains of two novel viral groups exhibit high similarity to SARS-CoV-2, and pseudovirus assays confirmed the ability of this spike protein to mediate entry into cells expressing human ACE2, suggesting a potential zoonotic risk. The observed genetic diversity underscores the urgent need for continuous surveillance to identify high-risk animal-to-human interfaces and inform pandemic preparedness.

## Abstract (web summary)

Bat coronaviruses pose potential pandemic risks. Here, the authors show extensive sarbecovirus diversity in Rhinolophus bats from Cambodia, revealing novel lineages with regional circulation, human ACE2-binding capacity, and highlighting the need for continued viral surveillance.

## Introduction

The emergence of SARS-CoV and SARS-CoV-2 in recent decades has significantly underscored the zoonotic potential inherent within coronaviruses, particularly those of the sarbecovirus subgenus^1^. These events substantiated the urgent necessity to explore the extensive genetic diversity of coronaviruses, especially within wildlife reservoirs, to better understand the mechanisms driving their evolution and transmission. A comprehensive understanding of the diversity and distribution of sarbecoviruses is essential to assess their potential spillover risks and the mechanisms by which they might cross regions and species boundaries, and to inform public health strategies aimed at preventing future pandemics^1^.

Since the onset of the COVID-19 pandemic, our knowledge of sarbecoviruses has expanded, including the detection of these viruses in various animal species^2,3^ and notably among insectivorous horseshoe bats (genus *Rhinolophus*) across Asia^4^. Close relatives of SARS-CoV-2 have been identified in several regions, including China (in *R. affinis, R. stheno, R. sinicus, R. marshalli, R. malayanus*, and *R. pusillus)*^5–11^, but also in Japan (*R. cornutus*)^12^, in Thailand (*R. acuminatus*)^13^, in Cambodia (*R. shameli*)^14^, in Laos (*R. malayanus, R. marshalli*, and *R. pusillus*)^15^ and in Vietnam (*R. pusillus* and *R. affinis*)^16^. Some of these *Rhinolophus* bats have relatively wide geographical ranges and are known to co-roost in large numbers^11^. This behavior may facilitate viral transmission and recombination, highlighting the need for further research on their ecology. In the biodiverse regions of Southeast Asia, bats play important ecological roles (e.g., pollination, seed dispersal, pest control), and represent one of the most abundant groups of mammals^17^. Their role as reservoirs of viruses, including coronaviruses^4^, and how their ecology influence the spread, maintenance, and evolution of these potentially zoonotic viruses remain insufficiently studied. The complexity of ecological interfaces in these regions raises concerns about the potential transmission of coronaviruses from their bat reservoir to other mammalian hosts and ultimately the risk of future sarbecovirus emergence in humans^18^. Effective monitoring of coronavirus circulation in bat populations before their emergence in humans or other animals, alongside understanding the molecular and epidemiological factors influencing spillover events^11^, remains a critical public health challenge.

In this study, we monitored coronaviruses in bats from Cambodia’s Steung Treng province by conducting repeated cross-sectional samplings. This region is characterized by karst hills formations, where *Rhinolophus* spp. populations are known to roost, and where positive samples were previously identified in 2010^14^. Our study aimed to characterize the sarbecoviruses circulating in the region and investigate their evolutionary history and their potential zoonotic risk.

## Results

### Sample collection and testing

Between August 2020 and June 2023, we conducted eight field missions and captured and sampled 1462 bats from nine genera (Table 1). Of the 1462 rectal swab samples tested, 146 were positive with at least one method: 23 tested positive exclusively by sarbecovirus RT-qPCR^19^, 56 tested positive for pan-coronavirus by at least one of two RT-PCRs^20,21^, and 67 tested positive by sarbecovirus RT-qPCR and at least one of the pan-coronaviruses RT-PCRs (Supplementary Table 1 and Supplementary Fig. 1). The 90 (23 + 67) rectal swab samples positive by sarbecovirus RT-qPCR included *Rhinolophus* spp. *(R. acuminatus, R. chaseni, R. malayanus, R. microglobosus, R. pusillus*, and *R. shameli*), *Hipposideros* spp. (*H. armiger, H. larvatus*), *Lyroderma lyra*, *Megaderma spasma*, and *Taphozous melanopogon* as detailed in Table 1. We performed Sanger sequencing of the PCR products targeting distinct parts of the RNA-dependent RNA polymerase (RdRp) gene^20,21^ obtained for the 123 (56 + 67) samples that tested positive with at least one of the pan-coronavirus RT-PCRs. Samples were identified using the bat species sampled (e.g., Rmi for *Rhinolophus microglobosus*), location (STT for Steung Treng), followed by an ID number. Phylogenetic analysis of the partial RdRp sequences revealed a diversity of alpha and betacoronaviruses, and seven samples showed signals of potential co-infections (Supplementary Fig. 2, highlighted with a red circle, and Supplementary Table 1): sample RmiSTT521 contained a partial RdRp sequence closely related to alphacoronaviruses (*Rhinacovirus*), and we obtained a near-complete genome sequence of a sarbecovirus by high-throughput sequencing (see below). The partial RdRp sequences of samples RshSTT288, RshSTT307, RshSTT313, and RshSTT517 clustered with alphacoronaviruses (*Decacovirus*), while samples RshSTT503 and RshSTT523 clustered within betacoronaviruses (*Hibecovirus*). In agreement with the late Ct values (>32) obtained in the sarbecovirus RT-qPCR for these samples, we were only able to obtain a partial sarbecovirus RdRp sequence for two of those samples (RshSTT307 and RshSTT313; sequencing attempts failed for samples RshSTT288, RshSTT503, RshSTT517, and RshSTT523). These three results, supported by sequence data, confirm co-infections involving betacoronaviruses and different alphacoronaviruses. While co-infection is the most likely explanation, we cannot rule out other factors contributing to the signal observed for the four multi-positive samples without sarbecovirus sequence data, such as recombinant viruses. Similarly, two samples among the 56 that tested negative for sarbecoviruses by RT-qPCR but were positive for at least one of the pan-coronaviruses RT-PCRs (HlaSTT489 and HlaSTT508) contained two distinct partial RdRp sequences. Phylogenetic analysis showed that one sequence clustered with alphacoronaviruses (*Decacovirus*) and the other with betacoronaviruses (*Hibecovirus*), also indicating likely co-infections. However, the presence of a recombinant virus cannot be excluded.

**Table 1:** Bat species captured by date of collection and tested by sarbecovirus real-time RT-qPCR^19^ and pan-coronavirus RT-PCRs^20,21^, at the four sites during active, longitudinal surveillance in Steung Treng, Cambodia, between 2020 and 2023

Genus/Species	Nb. of samples tested	Prevalence of samples positive for CoV (%) by date of sample collection								Nb. of samples positive by sarbecovirus RT-qPCR^a^	Nb. of samples positive by pan-CoV RT-PCRs^b^	Nb. of samples positive by both RT-PCRs^c^	Nb. of samples for sequencing
		Aug-20	Oct-2020	Mar-2021	Aug-Sep-2021	Nov-Dec-2021	Mar-2023	May-2023	Jun-2023				
*Cynopterus spp.*	4				0/4 (0%)								
*Cynopterus sphinx*	28		0/1 (0%)	0/6 (0%)	0/1 (0%)	0/16 (0%)		0/2 (0%)	0/2 (0%)				
*Eonycteris spelaea*	6			0/1 (0%)	0/2 (0%)	0/1 (0%)			0/2 (0%)				
*Hipposideros armiger*	33	0/3 (0%)	0/3 (0%)		0/10 (0%)		0/2 (0%)	**1/9 (11%)**	0/6 (0%)	**1/33 (3%)**			1
*Hipposideros cineraceus*	2					0/2 (0%)							
*Hipposideros diadema*	7						0/1 (0%)	0/5 (0%)	**1/1 (100%)**		**1/7 (14%)**		
*Hipposideros galeritus*	5		0/1 (0%)	0/1 (0%)		0/1 (0%)	0/1 (0%)	0/1 (0%)					
*Hipposideros larvatus*	55	**3/13 (23%)**	0/4 (0%)	0/3 (0%)	0/2 (0%)	0/1 (0%)	0/3 (0%)	**1/13 (8%)**	**8/16 (50%)**	**1/55 (2%)**	**11/55 (20%)**		1
*Hipposideros pomona*	11		**1/3 (33%)**				**1/2 (50%)**	0/1	**3/5 (60%)**		**5/11 (45%)**		
*Hypsugo dolichodon*	2								0/2 (0%)				
*Lyroderma lyra*	39	0/4 (0%)	0/7 (0%)		0/3 (0%)			0/2 (0%)	**1/23 (4%)**	**1/39 (3%)**			1
*Megaderma spasma*	30	0/2 (0%)	0/5 (0%)	0/2 (0%)	0/1 (0%)	**1/5 (20%)**	0/4 (0%)	0/4 (0%)	**1/7 (14%)**	**1/30 (3%)**	**1/30 (3%)**		1
*Rhinolophus acuminatus*	8							**5/8 (63%)**				**5/8 (63%)**	5
*Rhinolophus chaseni*	14					0/2 (0%)	0/1 (0%)	**3/10 (30%)**	0/1 (0%)	**2/14 (14%)**	**1/14 (7%)**		2
*Rhinolophus malayanus*	40	0/2 (0%)	0/15 (0%)	0/10 (0%)		0/1 (0%)	0/7 (0%)	0/1 (0%)	**1/4 (25%)**			**1/40 (3%)**	1
*Rhinolophus microglobosus*	34	**2/7 (29%)**	**1/4 (25%)**	0/2 (0%)		**3/9 (33%)**	0/6 (0%)	**3/3 (100%)**	**2/3 (67%)**	**1/34 (3%)**	**7/34 (21%)**	**3/34 (9%)**	4
*Rhinolophus pusillus*	14					0/5 (0%)	0/3 (0%)	**1/2 (50%)**	0/4 (0%)			**1/14 (7%)**	1
*Rhinolophus shameli*	872	**5/123 (4%)**	**4/80 (5%)**	**2/75 (3%)**	**1/63 (2%)**	**5/165 (3%)**	**10/158 (6%)**	**41/92 (45%)**	**33/116 (28%)**	**15/872 (3%)**	**29/872 (3%)**	**57/872 (7%)**	32#
*Rhinolophus spp.*	5					0/5 (0%)							
*Rousettus amplexicaudatus*	11					0/11 (0%)							
*Rousettus spp.*	5					0/5 (0%)							
*Taphozous melanopogon*	237	0/11 (0%)	0/15 (0%)	0/17 (0%)	0/40 (0%)	**1/92 (1%)**	0/14 (0%)	0/11 (0%)	**1/37 (3%)**	**1/237 (0.4%)**	**1/237 (0.4%)**		1
**Total**	1462	**10/165 (6%)**	**6/138 (4%)**	**2/117 (2%)**	**1/126 (0.8%)**	**10/321 (3%)**	**11/202 (5%)**	**55/164 (34%)**	**51/229 (22%)**	**23/1462 (2%)**	**56/1462 (4%)**	**67/1462 (5%)**	50

*Nb.* Number, *CoV* coronavirus.

^a^Real-time RT-qPCR detecting sarbecoviruses using a duplex assay targeting the E and N genes (19).

^b^RT-PCRs detecting pan-coronaviruses targeting the RdRp gene (20,21).

^c^One bat sample was positive for both the sarbecovirus real-time RT-qPCR and the pan-coronaviruses RT-PCRs.

# Positive *Rhinolophus shameli* bat samples in 2023 were selected for sequencing based on a Ct value lower than 31, as well as their geographic locations and sampling dates.

Bold values highlight prevalence greater than 0%.

### Identification of novel bat coronaviruses

We used metatranscriptomic and amplicon-based approaches to generate sequence data from a selection of 50 bat samples that tested positive for sarbecovirus RNA. We obtained 33 full-length and 7 partial genome sequences (Supplementary Table 2 and Supplementary Fig. 3), which were used to infer an initial maximum likelihood (ML) phylogenetic tree with publicly available sarbecoviruses sequences (Supplementary Fig. 4). With all the limitations related to recombination in consideration^22^, the phylogenetic analysis revealed the co-circulation of multiple SARS-CoV-2 related viruses in bat populations in Cambodia, most of which were not previously identified or characterized.

The seven partial sequences were excluded from further analyses due to their lower quality. Of note, four of them corresponded to non-*Rhinolophus* bat samples (*H. larvatus, L. lyra*, *M. spasma*, and *T. melanopogon*) with low viral load based on RT-qPCR results (Supplementary Table 3). Although extreme care was employed to limit the risk of cross-contamination during sample collection and processing, these results may not reflect productive infections in these bats, and further sampling will be needed to understand whether these viruses can indeed infect these bat species.

One sample (RshSTT564) presented numerous intrahost single nucleotide variants (iSNVs) dispersed throughout the genome at a frequency of ~0.23 [0.21 to 0.25], suggestive of contamination or co-infection with a closely related virus. This result was reproduced following an independent RNA extraction and sequencing run (Supplementary Fig. 3). Using the intra-host iSNVs frequency data, we generated an alternate RshSTT564 genome corresponding to the minor variant population (Supplementary Fig. 4). The constellation of mutations in this genome was distinct from all other consensus sequences generated in this work, suggesting that it does not correspond to cross-contamination during library preparation. All virus isolation attempts on both simian (Vero) and bat (Rhileki) cells were unsuccessful. This may be due to the viral load, the matrix (rectal swabs), but also to degradation occurring during sampling or freezing. Finally, we cannot exclude that other factors required for the replication of these viruses are absent in the cell lines tested.

### Evolutionary history of sarbecoviruses circulating in Cambodia

**Fig. 1:**
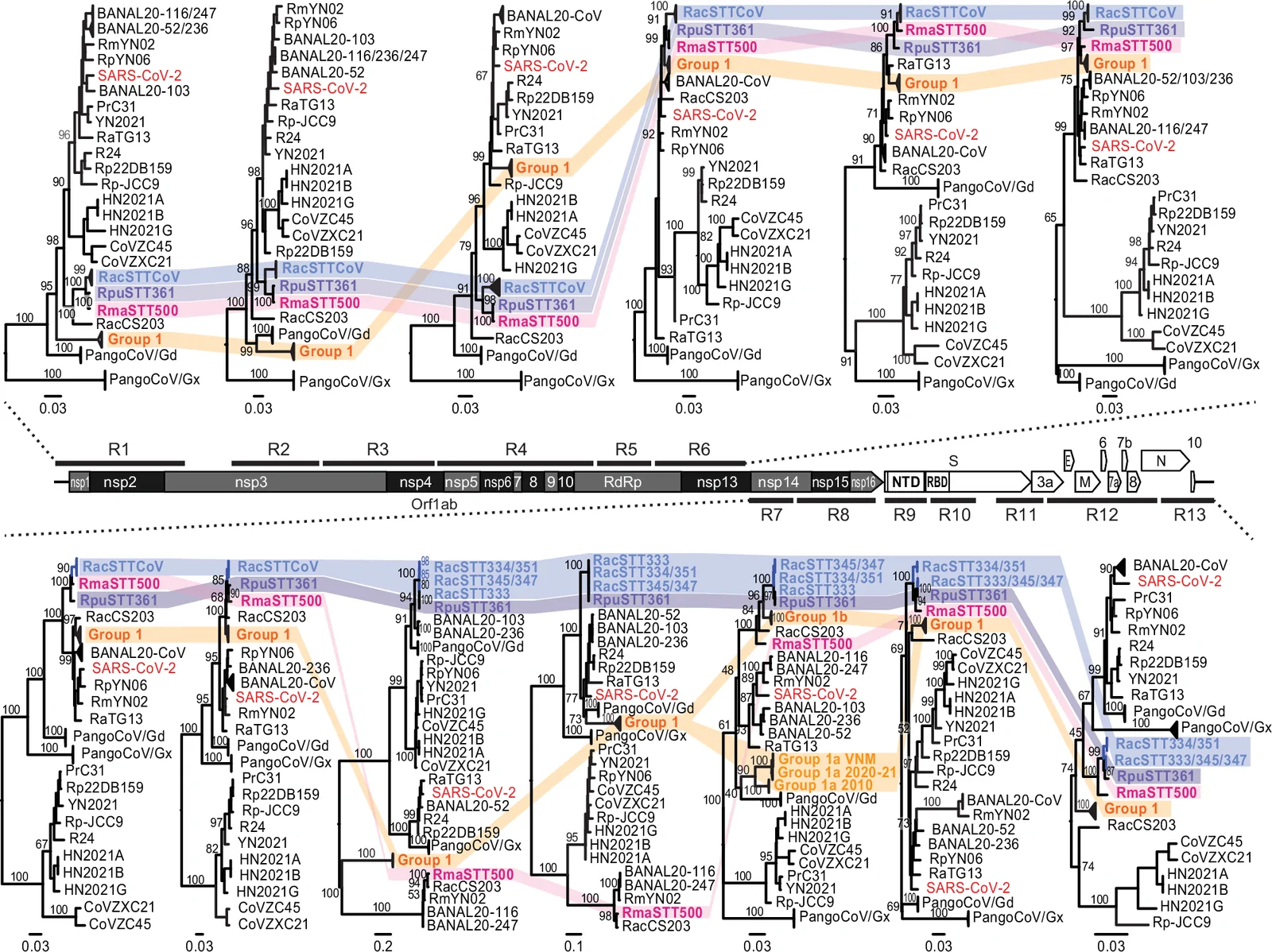
Maximum likelihood phylogenetic trees of the genomic regions identified by recombination analysis. The positions of the 13 genomic regions analyzed are shown on a sarbecovirus genome scheme. Annotated features include ORF1ab with its nonstructural proteins (nsps), the N-terminal domain (NTD) and receptor-binding domain (RBD) of the spike (S) gene, followed by ORF3a, envelope (E), membrane (M), ORF6, ORF7a, ORF7b, ORF8, nucleocapsid (N), and ORF10. The different mosaic patterns are highlighted in colors: group 1 in orange (with lighter and darker shades in region 11 for subgroups 1a and 1b, respectively), group 2 in blue, group 3 in purple and group 4 in pink. Branch support values obtained from 1000 ultrafast bootstrap replicates are shown on relevant nodes. Branch scale bars represent substitutions per site. Complete (non-collapsed) phylogenies are presented in Supplementary Fig. 4.

The evolution of sarbecoviruses is characterized by frequent recombination events^18,23^, making it challenging to reconstruct their evolutionary history using a single phylogeny. We thus used methods implemented in RDP4^24^ (Recombination Detection Program) to identify recombinant regions within the novel sequences. Based on recombination and phylogenetic analyses (Fig. 1 and Supplementary Fig. 5), we identified distinct genomic patterns among the sarbecoviruses circulating in Cambodia. We categorized these viruses into four groups based on their mosaic genome organization, spike receptor binding domain (RBD) sequence and sampling hosts.

The most prevalent sarbecoviruses (designated here as group 1) were found primarily in *R. shameli* samples, the most frequently captured bat species in this study (59% of total bats), as well as in *R. microglobosus* (Table 1). These genomes, sampled in 2020, 2021, and 2023, are closely related to two viruses previously detected in samples collected from *R. shameli* bats in the same area in 2010^14^, and to four genomes recently reported from Vietnam in 2022 from *R. affinis* bats^16^. Our analysis identified two mosaic patterns within group 1 (designated as 1a and 1b), with evidence of recombination in the spike S2 domain, while the viruses are otherwise monophyletic in all other parts of the genome (Fig. 1, region 11). However, it is unclear whether the subset 1a, which includes the 2010 viruses, are the recombinants, having acquired a portion of S2 from a divergent lineage, or if instead it corresponds to the ancestral sequence of group 1 viruses, with subset 1b having recombined with viruses of the groups 2, 3, or 4 lineages. Nevertheless, the absence of a close relative to the divergent S2 sequence suggests that at least one additional sarbecovirus lineage, currently unsampled, circulates or has circulated in recent years in the region. In parallel, groups 2, 3, and 4 viruses, which were sampled from *R. acuminatus*, *R. pusillus*, and *R. malayanus*, respectively, constitute a novel subclade within the sarbecovirus phylogeny. These viruses cluster together in most regions of the genome (Fig. 1), with branching variations suggestive of inter-group recombination. Of note, the group 4 virus, together with RacCS203, cluster separately from groups 2 and 3 in the regions of the spike. The grouping of groups 2 and 3 and the divergence with group 4 is also visible from a similarity analysis (Supplementary Fig. 6 and Supplementary Table 4).

RacCS203 was identified in Thailand in 2020, and it is also found basal to the viruses sampled in Cambodia in multiple other parts of the genome, further suggesting both common ancestry and genetic exchanges during the evolutionary history of the viruses found in these neighboring countries. Finally, our selective pressure analysis across the full dataset did not identify codon sites under positive selection with different methods, although one site (6957) detected by two methods was close to statistical significance (Supplementary Table 5).

To better understand the evolutionary histories of the sarbecoviruses co-circulating in Cambodia, we inferred time-measured phylogenies using a Bayesian phylogenetic approach for each of the 13 identified genomic regions as well as for a non-recombining alignment (NRA, see “Methods”) (Supplementary Fig. 7). In addition, as our data also allow the study of the continuous evolution of group 1 viruses lineage, which spans a 13-year window, we separately studied this subset of viruses, aiming to leverage complete genome data. However, as viruses within this group present evidence of recombination in the spike S2 domain (region 11), we conducted the phylodynamic analysis focusing on the genome truncated from region 11, which corresponds to 95.7% of the complete alignment (Supplementary Fig. 8). We used two datasets: group 1a viruses only, as they appear to have a continuous evolutionary history, and the complete group 1 viruses set that includes 1b viruses with a possibly distinct history. Using these near-complete genome data, the substitution rate estimated for group 1a was 6.82 × 10^−4^ substitutions per site yr^−1^ [95%HPDs 3.59 × 10^−4^–9.15 × 10^−4^], and slightly faster for the complete group 1 dataset (1.02 × 10^−3^ substitutions per site yr^–1^ [95%HPDs 7.76 × 10^−4^–1.26 × 10^−3^], Supplementary Fig. 9). Our estimates are within the range of the mean estimates previously reported for sarbecoviruses using partial (non-recombinant regions) genome data^25^, and comparable to estimates obtained for SARS-CoV-2^26^ or MERS-CoV^18^. These substitution rates may be affected by the small sample size and relatively short time range and may also reflect ongoing purifying selection processes. In agreement with the time-dependent rate phenomenon^27^, estimates over longer timescales could differ, and they may also be specific to this lineage, as variations have been noted within the *Sarbecovirus* phylogeny. Similarly, we noted variation in the substitution rates estimates across the 13 non-recombinant regions and the NRA, likely reflecting a combination of factors including alignment length, genomic location and potential functional constraints, or selective pressures (Supplementary Fig. 9).

**Fig. 2:**
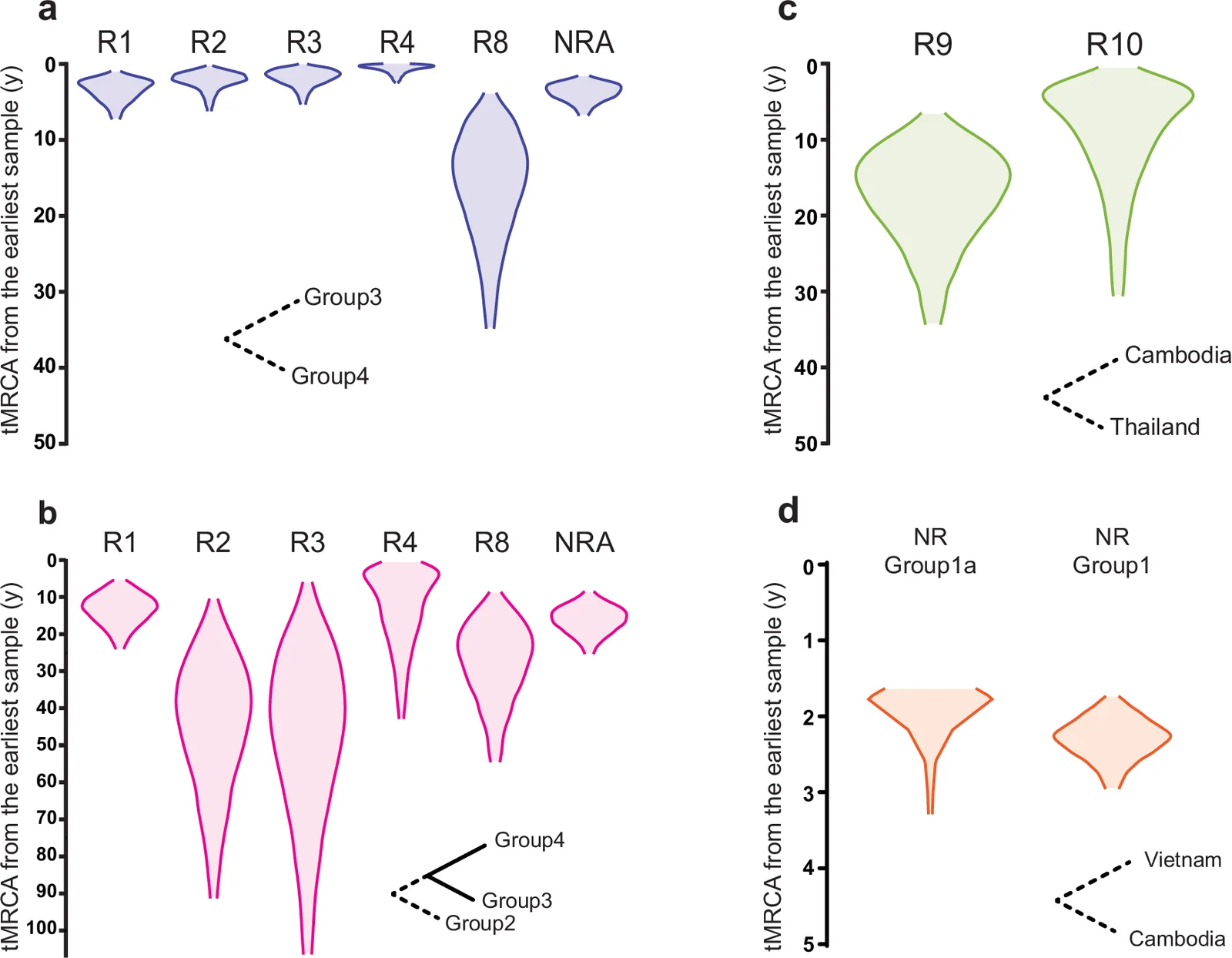
Distribution of the estimated time to the closest inferred ancestor of the viruses from bats sampled in Cambodia, estimated from different regions of the genome. **a** Distributions for the time to the closest ancestor to viruses from groups 3 and 4, estimated using regions 1–4, 8 and the non-recombining alignment (NRA). **b** Distributions for the time to the closest ancestor to viruses from group 2 and the ancestor to groups 3 and 4, estimated using regions 1–4, 8 and the NRA. **c** Distributions for the time to the closest ancestor to viruses from group 4 and the RacCS203 virus sampled in Thailand, estimated from regions 9 and 10. **d** Distribution of the time to the closest ancestor to virus from group 1 sampled in Vietnam, and viruses sampled in Cambodia. The violin plots represent the 95% highest posterior density (HPD) intervals of time using the oldest sample of each pair of clades as a reference time point. The corresponding violin plots visualized as calendar dates are presented in each of the Maximum Clade Credibility (MCC) trees in Supplementary Fig. 5.

Despite this variation, our results, both from the phylodynamic and the recombination analyses focusing on divergence times between specific lineages in relevant genomic regions, are compatible with multiple genetic exchanges over time between viral lineages sampled both within and outside Cambodia. Group 3 and group 4 viruses are monophyletic in 5 of the 13 genomic regions and in the NRA, and we estimate a median time to the most recent common ancestor (tMRCA) as recent as 6.5 months prior to the earliest sampling (Fig. 2a, and tMRCA visualized as dates in Supplementary Fig. 7), with short estimates obtained for 4 regions and the NRA. Within the same 5 regions and the NRA, the earliest median tMRCA for group 2 viruses with groups 3–4 viruses was estimated at 11.5 years (Fig. 2b), although with greater variation between regions, as expected for a deeper node. Based on regions 9 and 10, corresponding to mostly the spike, we estimated that a common ancestor to the group 4 virus and the RacCS203 virus sampled in Thailand, ~470 km away from the Steung Treng region of Cambodia, cocirculated with these lineages 7.8 to 14 years prior to the sampling [95% highest posterior density HPD 0.5–31.2 years] (Fig. 2c). Finally, within group 1, the viruses from Vietnam share a common ancestor with viruses from Cambodia with a median divergence time estimate of July 2020 to January 2021 (using group 1 and 1a respectively) (Supplementary Fig. 8). This suggests that these viruses would have spread across a distance of ~345 km (between Cambodia’s Steung Treng province and Vietnam’s Quang Tri province) within 1.74 to 2.94 years [95% HPD for group 1] or within 1.64 to 3.27 years [95% HPD for group 1a] (Fig. 2d).

### Analysis of the spikes and RBDs

We then focused our analysis on the spike protein of the different lineages co-circulating in Cambodia. First, considering that the four groups of viruses identified here were found in distinct bat species, we generated a tanglegram between a spike amino-acid phylogeny (again with the limitations due to recombination in mind) and the angiotensin-converting enzyme 2 (ACE2) amino acid sequences phylogeny of representative bat species, pangolin and human (Supplementary Fig. 10a). This comparison revealed a clustering pattern with the 3 bat species associated with group 1 viruses form a distinct clade from the bat species harboring viruses from groups 2, 3, and 4.

**Fig. 3:**
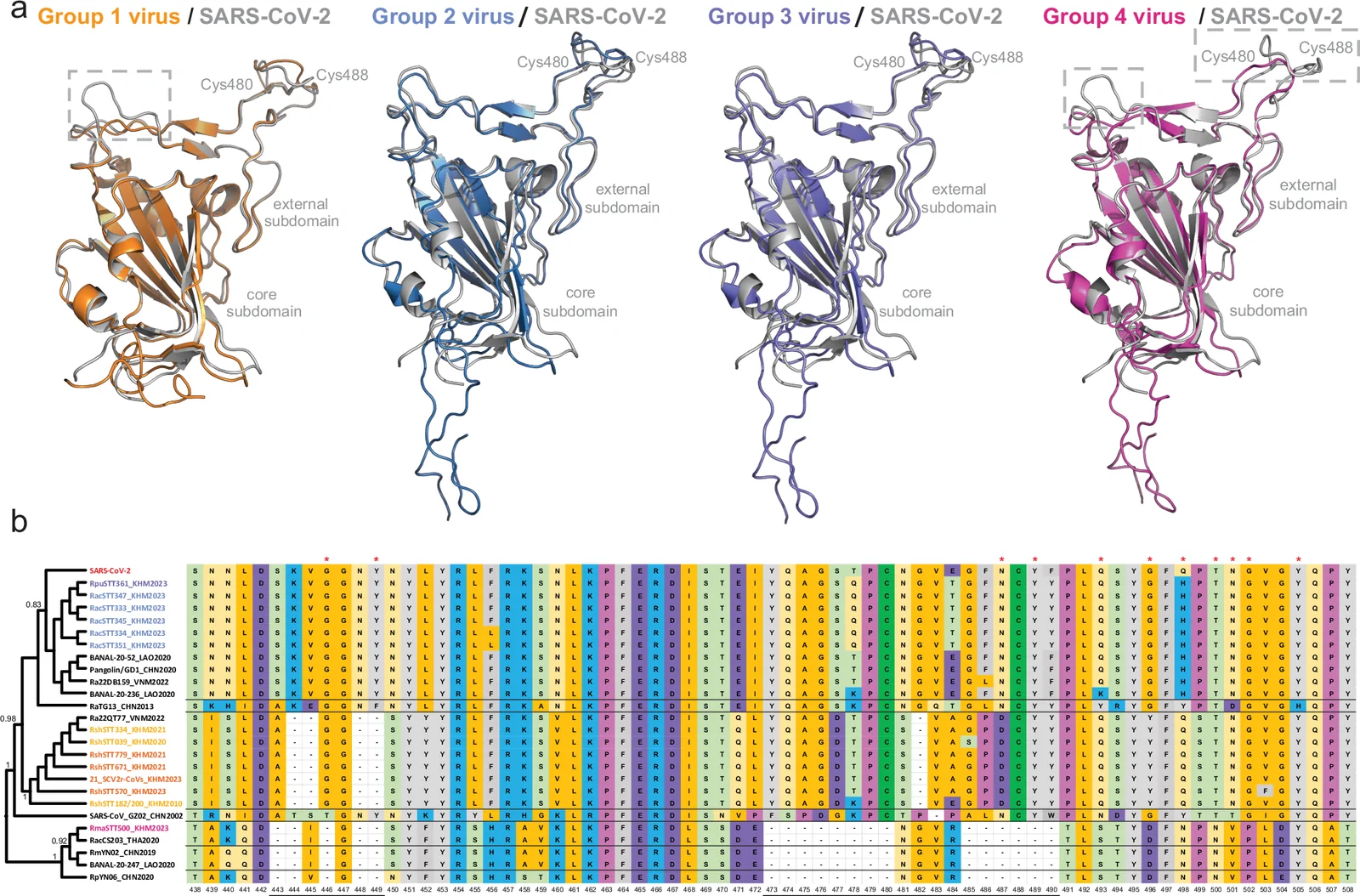
Analysis of the spike receptor-binding domain (RBD) of SARS-CoV-2 related bat viruses sampled in Cambodia. **a** Homology modeling of the structure of the RBDs of the 4 groups of viruses from Cambodia compared to SARS-CoV-2 RBD structure (PDB: 6yla.1). The three-dimensional structure of the RBDs of the viruses from Cambodia were modeled using the Swiss-Model program using the most fitting template. The structure of RshSTT182/200 RBD (PDB: 7xbh.1) was used for group 1 virus (orange and dark orange), the structure of BANAL-20-52 Spike trimer (PDB: 8hxj.1) was used for group 2 (light blue) and 3 (dark blue) viruses, and the structure coronavirus spike protein (PDB: 8aja.1) was used for group 4 virus (pink). **b** Alignment of the receptor binding motif (RBM) amino acid sequences of selected sarbecoviruses. Residues reported as involved in the interaction between SARS-CoV-2 RBD and the human ACE2 receptor^28^ are marked with a red asterisk (*). On the left, a cladogram shows the phylogenetic relationships among the RBM sequences. Branch support values superior to 0.8 are shown.

Next, we used homology modeling and sequence analysis to compare the external subdomain of each novel spike RBD structure to the SARS-CoV-2 RBD structure, and found that they are globally similar with some specific differences (Fig. 3). Groups 2–3 RBDs are highly similar to SARS-CoV-2 RBD, with only one amino acid difference among positions identified as contact residues for human ACE2 (hACE2) receptor binding^28^. This type of receptor-binding motif (RBM), also observed in viruses identified in Laos^15^, has been associated with broad ACE2 tropism, including efficient hACE2 binding. In contrast, group 1 viruses RBDs present two to three differences at key contact residues, as well as a shorter loop at the beginning of the RBM (spanning residues S:443–450, SARS-CoV-2 numbering), and a single amino acid deletion in loop 469–491 (Fig. 3). This pattern has been associated with tropism to a narrower range of ACE2 orthologs and found generally incompatible with efficient hACE2 usage. Finally, and consistently with the analysis of the RacCS203 and RmYN02 virus RBDs^8,13^, the group 4 virus displayed marked structural differences with SARS-CoV-2 RBD, including two shorter loops at the N terminal and central regions of the RBM, with the absence of residues involved in a conserved disulfide bond, and variations at most positions identified as contact residues for hACE2 receptor binding (Fig. 3). Conversely, we also noted variations in sites identified as proximity or interacting sites among ACE2 sequences of the corresponding bat host species and their close relatives (Supplementary Fig. 10b). None of the newly identified viruses harbored a poly-basic (Furin) cleavage site as present at the S1/S2 junction in SARS-CoV-2 (Supplementary Fig. 11). However, the group 4 virus, like viruses from Thailand and China, presents a three amino acid insertion (PVA) at this site.

### Functional assays

**Fig. 4:**
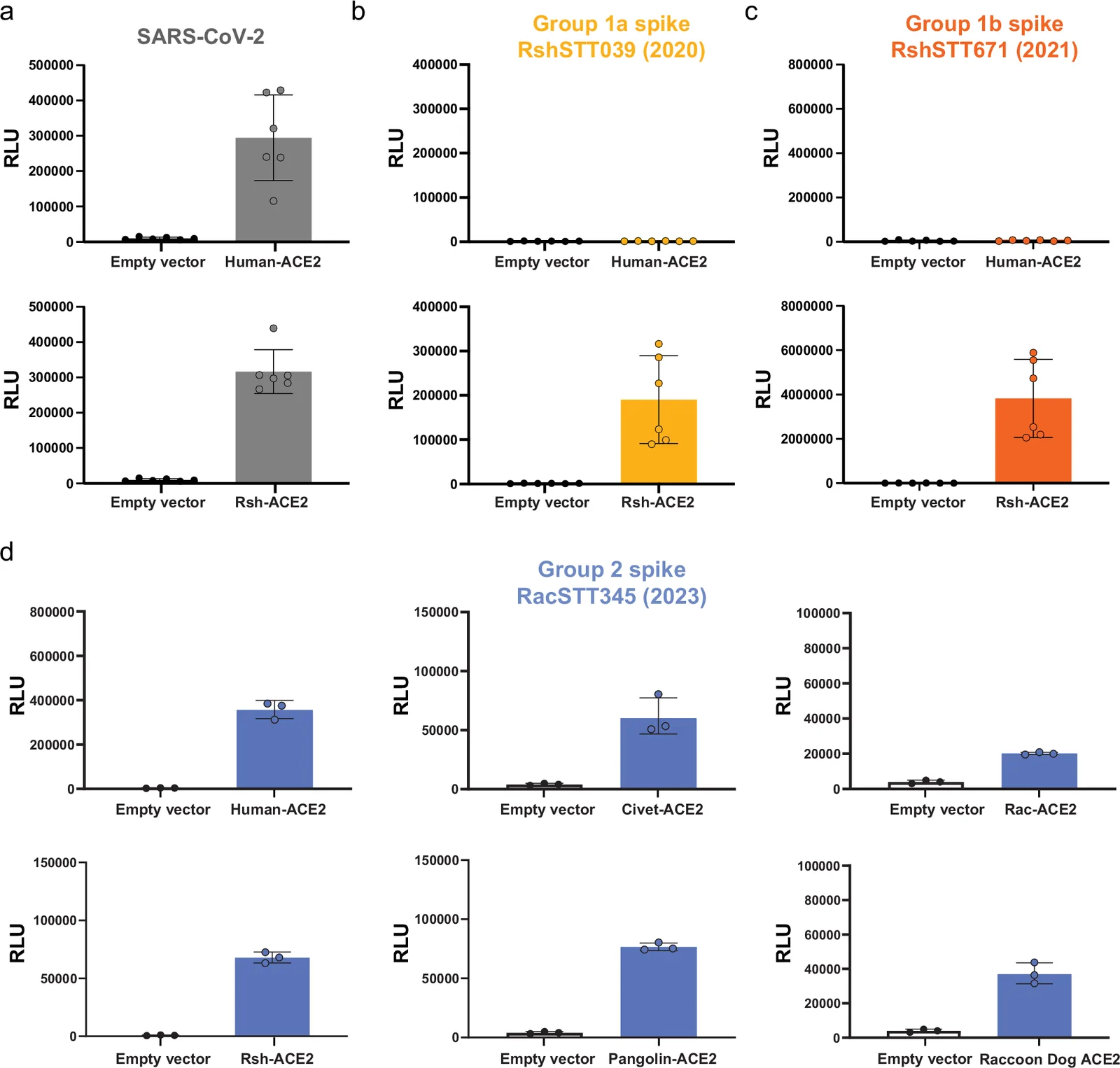
Pseudovirus entry assays. Entry into HEK293T cells transfected with either human ACE2 (hACE2), *Rhinolophus shameli* ACE2 (RshACE2), *Rhinolophus acuminatus* ACE2 (RacACE2), Civet ACE2, Pangolin ACE2, raccoon dog ACE2, or the corresponding empty vector, of pseudoviruses expressing the spike of **a** SARS-CoV-2 (grey), **b** group 1a RshSTT039 (orange), **c** group 1b RshSTT671 (dark orange), and **d** group 2 RacSTT345 (blue). **a**–**c** data were generated using a pLenti-puro plasmid to transfect the HEK293T cells, and **d** data using a pWPI-Zeo plasmid. Data are expressed in relative luminescence units (RLU) measured 48 h post-pseudovirus inoculation and are represented as mean ± standard deviation of technical replicates for two independent experiments (*n* = 3: RacSTT345, *n* = 6: SARS-CoV-2, RshSTT039, RshSTT671). Source data are provided as a Source Data file.

To further investigate the potential for human infection, we used a lentivirus-based pseudovirus system to assess the ability of representative spikes proteins from group 1 and groups 2–3 viruses to mediate entry into cells expressing hACE2, with the SARS-CoV-2 (Wuhan-Hu-1) spike as a control (Fig. 4a). In agreement with the homology modeling, the group 2 virus (RacSTT345) spike was able to mediate entry into cells expressing hACE2 (Fig. 4d), suggesting a lower barrier for spillover. We also tested the ability of RacSTT345 spike to enter cells expressing other ACE2 from different species. This spike was able to mediate entry into cells expressing either *R. acuminatus* ACE2 (RacACE2), *R. shameli* (RshACE2), civet ACE2, pangolin ACE2, or raccoon dog ACE2.

On the other hand, as observed for the 2010 viruses, the recent group 1 spikes (with or without recombination in S2, RshSTT039_2020 (group 1a) and RshSTT671_2021 (group 1b) respectively) were not able to mediate entry into cells expressing hACE2, while they were able to mediate entry in cells expressing RshACE2 (Fig. 4b, c). Of note, previous work has reported low levels of entry into hACE2-expressing cells using a VSV-based pseudovirus system with the 2010 spike, which could be improved by a single amino-acid mutation^29^. The isolation of a group 1 virus will be crucial to clarify the zoonotic potential of this viral lineage.

## Discussion

In this study, we report the repeated cross-sectional sampling of bats belonging to nine genera in the Steung Treng region of Cambodia, with a particular focus on *Rhinolophus* species. Using multiple sequencing approaches, we generated 33 novel sarbecovirus genomes, uncovering a much greater viral diversity than previously observed in the country. Through recombination and phylogenetic analyses, we identified distinct genomic patterns and evidence of further, unsampled, diversity. Although these different lineages were found in distinct groups of bat species, all the sarbecoviruses identified in Cambodia appear to share a common evolutionary history, at least in part, as they cluster closely together in certain regions of their genomes in the context of the currently known diversity of sarbecoviruses (Fig. 1). In addition, variations in tree topologies across the different genomic regions are consistent with recombination—and thus past co-infection of a host—between those lineages circulating in Cambodia.

These genomic exchanges are not confined to lineages found in Cambodia. Viruses recently detected in Vietnam fall within the diversity of group 1 viruses and share a very recent ancestry, with estimates in the order of two years, despite the geographic distance between the sampling sites. Similarly, a virus identified in Thailand in 2020 (RacCS203) shows close genetic proximity to the viral lineages from Cambodia, branching basal or within their diversity in multiple regions of the genome, evidencing a common ancestry. Furthermore, the group 4 virus exhibits strong evidence of recombination in the spike gene with an ancestor of RacCS203. Together, these findings suggest frequent and relatively long-distance virus migrations have occurred, potentially via bat migrations or transmissions across neighboring populations. This is particularly notable in very recent years for group 1 viruses, possibly facilitated by the dense forest environment that spans Northern Cambodia and Vietnam. Further studies will be necessary to determine whether the ongoing land-use changes in Southeast Asia, which may impact bat habitats, have contributed to these movements, alongside the potential effects of climate change^30^.

Even within more restricted geographic areas, such as the sites surveyed in the Steung Treng province, the habitat-sharing behavior of diverse bat species appears to facilitate viral exchanges, offering opportunities to expand the genetic diversity of viral populations. Interestingly, the four groups of newly detected viruses were each found to be associated with distinct bat species, despite being captured in the same geographical area where they likely share the same or close habitats. While further sampling could alter this observation, the current observation may reflect finer co-roosting habits of these species, or host-virus compatibilities and virus adaptations distinct from spike-ACE2 receptor interactions. Indeed, ACE2 amino acid phylogeny groups the bat species harboring the sarbecoviruses identified in Cambodia into two broad clusters, and amino-acid variations among bat species ACE2 can be found at positions identified as interaction sites between hACE2 and SARS-CoV-2 RBD^31^ (Supplementary Fig. 10). However, the evidence of recombination and functional assay data indicate that intergroup infections can occur. The 2010 group 1 spike has been shown to be able to mediate entry in cells expressing *R. malayanus* ACE2^32^ and we showed here that a group 2 spike is compatible with *R. shameli* ACE2. Isolating these viruses would be key to understanding these potential restrictions. Similarly, further sampling would also help to determine whether the low viral load signal and partial sequence data obtained from several non-*Rhinolophus* bats samples reflect productive infections, as this would come to challenge our understanding of the extent of the host range of these viruses.

Finally, as a crude indicator of emergence risk, we assessed the capacity of spike proteins from groups 1 and 2 to mediate entry into cells expressing hACE2. While the recent spikes of group 1 viruses were not compatible with human ACE2 in our pseudovirus system, consistent with previous findings with a spike from 2010^14^, the group 2 spike (RacSTT345) was able to mediate entry, suggesting potential for spillover. Interestingly, this spike was also able to mediate entry into cells expressing RshACE2, but also Civet ACE2, Pangolin ACE2 or Raccoon dog ACE2 in addition to RacACE2, suggesting that small mammals could be a potential intermediate host in case of cross-species transmission. This, with the high similarity with SARS-CoV-2 RBD amino acid sequence, suggests that groups 2 and 3 spikes may also be generalists in terms of host tropism^33^. However, there is currently no indication of a significant threat to public health, given the absence of a furin cleavage site. Additionally, immunity induced by SARS-CoV-2 exposure or vaccination may offer cross-protection against these viruses, considering the high amino acid similarity of the groups 2 and 3 spike RBDs to SARS-CoV-2, reaching up to 96.8% identity—a value comparable to the highest similarity reported for viruses from Laos^15^. Interestingly, the nucleotide-level similarity of groups 2 and 3 RBDs is lower, with numerous synonymous mutations compared to SARS-CoV-2. This observation could reflect a combination of factors, including differences in evolutionary rates, the accumulation of selectively neutral mutations over time, or host adaptation (at the species level) through codon usage optimization.

This work underscores the extensive genomic diversity of coronaviruses co-circulating in a small area of northern Cambodia and indicates both local and regional lineage migrations over short periods. Expanding surveillance efforts geographically and longitudinally will be crucial to identify high-risk animal-to-human interfaces and track their evolution to strengthen future pandemic preparedness efforts.

### Limitations of the study

Our work has several limitations. First, we were unable to isolate any of the viruses reported here. Successfully isolating or reconstructing these viruses via reverse genetics would allow for a proper assessment of their properties, host range, and a better understanding of their potential emergence risk. Additionally, it would be valuable to evaluate whether sera from individuals vaccinated against COVID-19 or with history of SARS-CoV-2 infection can neutralize these viruses. Second, although it was not the focus of this work, the timeline and frequency of sampling missions did not allow us to accurately capture the true temporal trends and ecological determinants of sarbecoviruses prevalence within these bat populations. Strong variations in the detection frequency over the years may be linked to the seasonality of bat gestation and birth, with synchronized pulses of immunologically naive individuals potentially facilitating the amplification of viral populations^34^. Further sampling efforts will be needed to reliably assess temporal patterns and enhance our understanding of the ecological drivers behind virus fluctuations in these bat communities. Despite this, our study revealed an extensive genetic diversity of sarbecoviruses found in a limited area within the Steung Treng province, with evidence of viral lineages movements. This highlights the need for broader sampling across the country and the region to better capture the extent and dynamics of sarbecoviruses circulation.

## Methods

### Sampling

Eight sampling sessions were conducted on the following dates: August 18–22, 2020; October 12–17, 2020; March 13–17, 2021; August 30-September 3, 2021; November 29-December 7, 2021; March 27–30, 2023; May 2–6, 2023; and June 12–17, 2023. Each mission spanned consecutive nights across four different karst hill locations (Phnom Chab Phleurng, Phnom Chhngauk, Phnom Ka Ngoak, and Phnom Kork Romeas) (Supplementary Fig. 1). Mist nets were set up each evening, either at the entrance of caves or on potential bat flyways and monitored during a consistent 2-h period per night. Capture, handling, species identification, sampling and release of live bats followed established standard procedures used by the study group and adhered to international standards for animal research^35,36^. For each captured bat, species, sex, age, and reproductive status of bat females were determined on site. Bat species were identified using morphological criteria in the field. The following biological samples were collected from captured bats: blood, oral swab, rectal swab, and wing punch. Swabs were collected twice, and individually stored, one in TRIzol™ reagent (Sigma-Aldrich) and one in viral transport medium (containing tryptose phosphate Broth 2.95%, 145 mM of NaCl, 5% gelatin, 54 mM Amphotericin B, 106 U of penicillin-streptomycin per liter, 80 mg of gentamycin per liter [Sigma-Aldrich, Steinheim, Germany]). All biological samples were stored on ice during collection and then transferred to liquid nitrogen within 24 h. Subsequently, they were kept at −80 °C at the Virology Unit of the IPC until further analysis. Downstream processing was conducted under biosafety level 3 (BSL-3) conditions.

### RNA extraction and molecular testing

RNAs were extracted from the rectal swab specimens stored in TRIzol™ reagent using the Zymo Research Direct-zol RNA MiniPrep kit (Zymo Research, CA, USA). RNA samples were subjected to real-time RT-qPCR^19^ targeting the E and N genes for sarbecovirus detection, and to two pan-coronavirus conventional RT-PCRs^20,21^ targeting distinct portions of the RdRp gene (Supplementary Table 6). These assays were selected with the aim of broadly detecting diverse coronaviruses, with a specific effort towards SARS-CoV-2-related viruses. The ref. ^21^ and ref. ^20^ protocols best detected coronavirus diversity but exhibited lower sensitivity for some sarbecoviruses. In contrast, the Corman et al. RT-PCR is more specific (narrower range) but is more sensitive for SARS-CoV-2-like viruses than the two nested RT-PCRs.

The amplification products of the positive RT-PCRs were Sanger sequenced (Macrogen, Inc., Seoul, Republic of Korea). The sequences were aligned using MAFFT v7.508^37^ with a subset of selected reference sequences retrieved on GenBank (https://www.ncbi.nlm.nih.gov/) and GISAID. The 294 nt long sequence alignment of the target region of ref. ^20^ includes 107 sequences from this study and 65 publicly available sequences. The 394 nt long alignment of the target region of ref. ^21^ (distinct from the Quan et al. target) includes 115 sequences from this study and 72 reference sequences.

The species of bats positive for a coronavirus were confirmed via DNA barcoding, using conventional PCR to amplify and Sanger-sequence the mitochondrial cytochrome c oxidase subunit 1 (CO1) gene using previously described primers^38,39^, followed by BLAST^40^ analysis. Additionally, the ACE2 of bats of interest were sequenced on an Oxford Nanopore Technology (ONT) GridION using previously described primers^41^.

### Sequencing and genome assembly

We employed a combination of sequencing approaches to attempt to obtain viral genomic sequences from the samples positive for sarbecoviruses by RT-qPCR. Ten samples were first processed with metatranscriptomic sequencing, and targeted amplicon sequencing was then used for 38 additional samples, and to attempt to complete partial genomes (Supplementary Fig. 3 and Supplementary Table 2). Metatranscriptomics allowed us to obtain data from novel or divergent viruses, but showed limited results with low input samples. Amplicon sequencing was a cost-effective approach to process related genomes from low-viral-load samples. For samples collected from *R. shameli* in 2023, a selection was performed based on Ct values < 31, geographic location and sampling dates. No-template controls were included at every step from extraction to sequencing to control for contamination, and no contamination was detected.

#### Metatranscriptomic sequencing

Metatranscriptomic sequencing following RNase H-based ribosomal RNA (rRNA) depletion was first utilized on two samples detected in 2020 and early 2021 (RshSTT039 and RshSTT334, respectively) and it was further conducted on two samples detected in 2023 (RshSTT570 and RacSTT351). Briefly, extracted RNA was treated with Turbo DNase (Ambion) before RNase H-based rRNA depletion (using custom DNA probes targeting human rRNA). After conversion into ds-cDNA, libraries were prepared with the Nextera XT library preparation kit (Illumina) and sequenced on an Illumina NextSeq 500 platform (2 × 75 cycles).

Metatranscriptomic sequencing was also attempted with other approaches. RNA samples were treated with Turbo DNase and subjected to sequence-independent single-primer amplification^42^. Treated RNA was reverse transcribed using the SISPA-Primer A (5′-GTTTCCCACTGGAGGATA-(N9)-3′) and SuperScript IV First-Strand kit according to the manufacturer’s instructions, followed by second-strand synthesis using Sequenase Version 2.0 DNA Polymerase (Thermo Fisher Scientific). The dsDNA was amplified with the SISPA-Primer B (5′-GTTTCCCACTGGAGGATA-3′) using Phusion High-Fidelity DNA Polymerase (Thermo Fisher Scientific), and sequenced on an ONT GridION.

Samples were also processed using the SMARTer Stranded Total RNA-Seq kit v3 (Takara Bio, USA) for cDNA synthesis and library preparation, and sequenced on the Illumina NovaSeq platform (Macrogen, Inc., Seoul, Republic of Korea).

#### Amplicon-based sequencing

Leveraging these novel data and the genomes from 2010 (EPI_ISL_852605), we designed a highly multiplexed PCR amplicon approach using Primalscheme^43^. Sequence gaps of RshSTT039 were subsequently bridged by Sanger sequencing. For samples with viruses divergent from the 2010 virus, additional amplicon sets were designed and used to re-sequence these samples (https://github.com/Simon-LoriereLab/BatCoVCambodia2020-23).

Amplicon sequencing was performed on an ONT GridION. RNAs were used to generate cDNA using SuperScript™ III First-Strand Synthesis SuperMix (Invitrogen, Carlsbad, CA, USA) or LunaScript® RT SuperMix Kit (NEB, E3010) and PCR amplicons were generated using the Q5 Hot Start High-Fidelity DNA Polymerase (NEB, M0493S). PCR products were multiplexed using ONT native barcodes and ran in batches of 12–24 samples per flow-cell (FLO-MIN106). The resulting reads were base-called and demultiplexed using Guppy (7.0.9) with a minimum quality score of 9.

The spike gene sequences of a selection of viruses identified as group 1 (RshSTT039, RshSTT671, RshSTT610, RshSTT570), and of all viruses of groups 2, 3, and 4 were confirmed by Sanger sequencing.

### Bioinformatic analyses

We employed a similar bioinformatic workflow for all metatranscriptomic data analyses^14^. After demultiplexing, reads were subjected to quality trimming and adapter removal. We then performed de novo assembly using the metaspades option of SPAdes v3.14.0 and MEGAHIT v1.2.9 for Illumina data and canu v2.2 for ONT data. Scaffolds were queried against the NCBI non-redundant protein database using DIAMOND v2.0.4. Scaffolds identified as sarbecovirus genomes were then used as template for iterative mapping using CLC Assembly Cell v5.1.0 (Qiagen) for Illumina reads, or minimap2 v2.17-r941 for ONT reads. Aligned reads were manually inspected using Integrative Genomics Viewer^44^ v2.12.3 (https://software.broadinstitute.org/software/igv/) and consensus sequences were generated using a minimum threshold of 10× read-depth coverage for Illumina and ONT data, except for RshSTT039, where a minimal coverage of 3 reads was used (Supplementary Fig. 3). iSNVs were determined using iVar v1.3.1 for Illumina reads.

The reads from amplicon sequencing were first filtered for minimum length of 100 nt using seqtk v1.3-r106. Reads were then processed using the ARTIC pipeline (https://github.com/artic-network/artic-ncov2019) to map the reads, trim the primers and generate a first consensus sequence. We also used iVar v1.3.1 to generate a second consensus. The consensus sequences were aligned, and potential artifacts were visually inspected. The cleaned consensus sequence was then used as a reference for a second mapping, and a final consensus was generated after primers trimming. All consensus sequences were produced using a minimum of 20× read-depth coverage. iVar v1.3.1 was used for iSNV analysis of a putative co-infection (sample RshSTT564). Coverage statistics of the partial and complete genomes obtained are presented in Supplementary Fig. 3.

### Dataset

Sarbecovirus genomes sequences and metadata available in the GenBank and GISAID EpiCoV databases (epi_set_250326kw, https://epicov.org/epi3/epi_set/250326kw) were retrieved in June 2024. Sequences with large stretches of missing data were removed, and a set of 34 genomes (Supplementary Table 7) were retained and selected for further analysis. We added the 33 complete, one alternate and 7 partial sequences generated here and sequences were aligned using MAFFT v.7.508^37^. The alignment was manually checked for accuracy using Geneious Prime 2023.0.1 (https://www.geneious.com/), resulting in a final alignment length of 29,991 nt.

### Phylogenetic inferences

All ML phylogenies were inferred using IQ-TREE v2.2.0^45^, with ModelFinder Plus to select the best-fit substitution model^46^, and branch support was estimated using the ultrafast bootstrap approximation with 1000 replicates^47^.

### Recombination analysis

We employed a combination of methods implemented in RDP4 v4.97^24^ (RDP, GENECONV, Chimaera, MaxChi, BootScan, SiSscan, and 3SEQ) to detect potential recombination events in the newly generated bat viruses genomes, and conservatively considered recombination signals detected by at least four methods. The coordinates of the detected breakpoints were used to partition the alignment, resulting in 13 regions. Of note, within these partitions, some sarbecoviruses not sampled in Cambodia and not directly related to the viruses from Cambodia also presented evidence of recombination. These genomes (highlighted in grey in Supplementary Fig. 5) were kept in these alignments as they were not the focus of the analyses, and the region of interest was not subdivided. Boundaries similar to those identified here for the viruses from Cambodia and related genomes have been reported in more exhaustive studies on recombination among all sarbecoviruses^25,48^, including some of the regions identified as hotspot for recombination^48^.

Following the approach described in ref. ^18^, we also used the results of the recombination analysis (here applied to all the genomes) to generate a NRA by keeping only the major non-recombinant region of each genome, masking minor recombination regions. Four sequences (PrC31, Rp22DB159, YN2021, and HN2021B) were excluded as they displayed recombination signals across their entire genomes. The novel alignment was reanalyzed with RDP4, resulting in an NRA containing 63 genomes.

Finally, we further analyzed the recombination signal detected in region R11 (spike C-terminal domain) within the highly related group 1 genomes, as no close relative corresponding to the donor parent lineage could be identified. To verify that the recombination signals detected could not be due to other processes such as strong selective pressures, we analyzed the nature and distribution of substitutions across the genomes of all group 1 viruses by comparing the 26 genome sequences collected in Cambodia and four from Vietnam to the two earliest sequences detected in 2010. In agreement with the recombination signal, the C-terminal region of the spike appeared enriched in changes compared to the rest of the genome, but mostly with synonymous mutations, a profile more compatible with the recombination hypothesis.

In parallel, similarity analysis was performed using SimPlot v3.5.1^49^ and the distance analysis tool implemented in Geneious Prime® 2025.0.3.

### Bayesian divergence time and evolutionary rate estimation

We used the 13 mosaic regions, the NRA, and non-recombinant regions of group 1 and group 1a for phylodynamic inferences. To examine temporal signal in each dataset, we first performed a root-to-tip regression analysis using TempEst v.1.5.3^50^ and the corresponding ML phylogenies. While all datasets showed temporal signal when selecting the root position that maximized *R*^2^, the signal was only strong for the group 1 viruses phylogenies, with and without the inclusion of the genomes sampled in 2010. We thus also used a marginal likelihood estimation procedure called BETS^51^ (Bayesian Evaluation of Temporal Signal) to further assess the temporal structure. We compared the statistical fit of two molecular clock models, strict and uncorrelated relaxed clock with an underlying lognormal distribution, and two configurations of sampling times: the correct sampling times and no sampling times. The BETS analyses provided positive (log) Bayes factors for the models with real sampling times over those with no sampling times, allowing us to confirm that all our datasets present temporal signal (Supplementary Table 8). In addition, the marginal likelihood and positive Bayes factor results from the BETS analysis indicated a better performance of the relaxed over the strict clock model.

We performed Bayesian phylodynamic analysis on each dataset using BEAST v.1.10.4^52^, with the GTR + I + G4 substitution model, and an uncorrelated lognormal relaxed clock, testing different model combinations of coalescent tree priors. We used posterior analyses with path (PS) and stepping-stone (SS) sampling methods to identify the best-fit demographic model (constant size, exponential growth, Bayesian Skygrid, Skyline and Skyride priors). MCMC samplings were run sufficiently long to ensure effective sampling sizes > 200.

According to the PS/SS analysis, the Bayesian Skyline was the best-fitting model for all datasets (Supplementary Table 9), except for the R11 dataset, which was better fitted by the Bayesian Skygrid model, and for group 1a dataset, better fitted by the Bayesian Skygrid model.

We used Tracer v.1.7.1 to summarize the evolutionary parameters, including the estimates of the substitution rate and the tMRCA for nodes of interest (Fig. 2 and Supplementary Fig. 9) and compared them to published estimates of coronaviruses. We reran a selection of BEAST analyses after excluding sequences identified as recombinant and did not observe differences in parameters estimates. The final trees were summarized using Tree Annotator v.1.10.4 and visualized using FigTree v.1.4.7.

### Selection analysis

The non-recombinant coding sequences (after removal of region 11, where recombination was identified) of the 32 sarbecoviruses genomes assigned to group 1 were used to perform selection analysis with DATAMONKEY, and the mixed effects model of evolution (MEME), adaptive branch-site random effects likelihood (aBSREL), fixed effects likelihood (FEL), single-likelihood ancestor counting (SLAC), and fast unconstrained Bayesian approximation (FUBAR) methods. Significance level was set with *p*-value threshold of 0.1 for MEME, FEL, and SLAC, and 0.05 for aBSREL, and with posterior probability of 0.9 for FUBAR. Site-by-site estimation of selection pressure was performed using the “dN-dS” test statistic to assess selection types on individual codons. A site detected by more than two algorithms was considered under selection.

Selective pressures were also measured with the non-synonymous to synonymous substitution rate ratio ω (dN/dS), where *ω* = 1, *ω* < 1, and *ω* > 1 were assumed to correspond to neutral, purifying, and positive selection, respectively. The ω ratio was estimated by the maximum-likelihood method implemented in EasyCODEML^53^, using four pairs of models (M0–M3, M1a–M2a, M7–M8, and M8–M8a).

### Structure modeling

The three-dimensional structures of the spike RBD of viruses identified in Cambodia was modeled using the SWISS-MODEL program^54^ and compared to the structure of the SARS-CoV-2 RBD (PDB: 6yla.1). The structure of RshSTT182/200 RBD (PDB: 7xbh.1) was used for group 1 viruses (RshSTT039: 1a and RshSTT671: 1b), BANAL-20-52 Spike trimer (PDB: 8hxj.1) for group 2 (RacSTT345) and 3 (RpuSTT361) viruses, and the SARS-CoV spike protein (PDB: 8aja.1) for group 4 virus (RmaSTT500).

### Cells and virus isolation

HEK293T (Sigma) and Vero (ATCC CCL-81) cells were maintained in Dulbecco’s modified Eagle medium (DMEM, Sigma-Aldrich) supplemented with 10% fetal bovine serum (FCS, Gibco) and 1% penicillin-streptomycin (Pen-Strep, Gibco). Rhileki (*R. lepidus*) cells, provided by the laboratory of Gavin J.D. Smith, were maintained in DMEM supplemented with 10% FCS, 1% nonessential amino acids (Gibco), 1 mM sodium pyruvate (Gibco), and 100 U/mL Pen-Strep. All cell lines were grown at 37 °C and 5% CO_2_.

Virus isolation was conducted in BSL-3 by inoculating Vero and Rhileki cells in parallel, followed by daily observation for cytopathic effect for 6 days. At the end of the observation period, cell culture supernatants were harvested and centrifuged to remove cellular debris. To screen for the presence of sarbecoviruses, a pan-sarbecovirus RT-qPCR^19^ assay was applied.

### Pseudovirus entry assay

Lentivirus pseudoparticles displaying different spike proteins of interest were produced using the system described by the Bloom laboratory^55^. The following reagent was obtained through BEI Resources, NIAID, NIH: SARS-Related Coronavirus 2, Wuhan-Hu-1 Spike-Pseudotyped Lentiviral Kit, NR-52948, kindly contributed by Alejandro B. Balazs and Jesse D. Bloom.

The sequences corresponding to the selected spikes deleted of the last 21 amino-acids and codon-optimized for expression in human cells were de novo synthesized (GeneArt, Life Technologies), and cloned into the pHDM expression plasmid from the lentiviral kit. The sequence of each construct was verified by Sanger sequencing.

Briefly, HEK293T cells were seeded in 10 cm dishes. The following day, the cells were co-transfected with 10 μg of pHAGE-CMV-Luc2-IRES-ZsGreen-W (NR-52516), 3.33 μg each of the helper plasmids HDM-Hgpm2 (NR-52717), HDMtat1b (NR-52518), and pRC-CMV-Rev1b (NR-52519), and 5 μg of a spike-expressing plasmid encoding either the SARS-CoV-2 spike or the selected spikes from virus group 1 (RshSTT200, RshSTT039, or RshSTT671) or from group 2 (RacSTT345). The transfection was performed using Lipofectamine 3000 (Invitrogen) according to the manufacturer’s protocol. The supernatants were harvested 72 h after transfection, clarified by centrifugation, aliquoted, and then stored at −80 °C.

To assay entry, HEK293T were seeded in 96-wells plates one day prior to transfection with either pLenti-puro-RshACE2, pHAGE2-EF1aInt-ACE2-WT (NR52512), or pLenti-puro (empty); or with pWPI-Zeo-hACE2, pWPI-Zeo-RshACE2, pWPI-Zeo-RacACE2, pWPI-Zeo-Civet ACE2, pWPI-Zeo-Pangolin ACE2, pWPI-Zeo-Raccoon dog ACE2 using Lipofectamine 3000 (Invitrogen) according to the manufacturer’s protocol. The day after transfection, media was removed and cells were transduced with pseudoparticles expressing one of the spikes with 5 µg/ml of polybrene transfection reagent (Merck-Millipore) in a final volume of 150 µl. Three days later, an equal volume of Bright Glo reagent (Promega) was added and mixed by pipetting. After 10 min of incubation, quantification was done with a Centro XS LB 960 luminometer (Berthold technologies). Two independent replicates were performed.

### Reporting summary

Further information on research design is available in the Nature Portfolio Reporting Summary linked to this article.

## Supplementary information

Supplementary Information

Reporting Summary

Transparent Peer Review file

## Source data

Source Data

## Data Availability

The raw sequencing data and the partial RdRp sequences were deposited on the European Nucleotide Archive (ENA, https://www.ebi.ac.uk/ena/browser/home) under BioProject PRJEB86850^56^. Assembled genomes were deposited on the GISAID EpiCoV database (https://gisaid.org/) with ID EPI_SET_250414kp 10.55876/gis8.250414kp and on the GenBank database (https://www.ncbi.nlm.nih.gov/genbank/) with accession numbers PZ487726-PZ487758. The CO1 and ACE2 sequences deposited on ENA under BioProject PRJEB87055. Primers designed in this study are available at https://github.com/Simon-LoriereLab/BatCoVCambodia2020-23 (10.5281/zenodo.20629491). Source data are provided with this paper.

## Code availability

This work has only used publicly available software using the default settings, and the corresponding publications are cited in the reference list.
