# Supplementary material for: Characterization and evolutionary history of novel SARS-CoV-2-related viruses in bats from Cambodia
Source: Nat Commun. 2026 Jul 23;17:9002. doi: 10.1038/s41467-026-75954-1 (PMC13507159; doi:10.1038/s41467-026-75954-1)
Supplement: Supplementary file 1 [file 41467_2026_75954_MOESM1_ESM.pdf]

Supplementary information for

Characterization and evolutionary history of novel SARS-CoV-2-related viruses in bats from Cambodia

Tey Putita Ou[1,2,3], Julia Guillebaud[1], Artem Baidaliuk[2,4], Sothyra Tum[5], Dany Chheang[6], Deborah Delaune[2], Matthieu Prot[2], Elise Bruder[2], Rafael Rahal Guaragna Machado[2], Leakhena Pum[1], Vibol Hul[1], Thavry Hoem[1], Sovann Ly[7], Heidi Auerswald[1], Frederick Arnaud[8], Kei Sato[9,10], Gavin JD Smith[10], Philippe Dussart[11], Erik A Karlsson[1], Véronique Chevalier[11,12,13], Julien Cappelle[13,14], Veasna Duong[1#], Etienne Simon-Loriere[2#]

Affiliations

[1]Virology Unit, Institut Pasteur du Cambodge, Pasteur Network, Phnom Penh, Cambodia
[2]Institut Pasteur, Université Paris Cité, CNRS UMR2000, Evolutionary Genomics of RNA Viruses unit, Paris, France
[3]Ecole Doctorale GAIA, University of Montpelier, 641 Av. du Doyen Gaston Giraud, Montpellier 34000, France
[4]Institut Pasteur, Université Paris Cité, Bioinformatics and Biostatistics Hub, Paris, France
[5]General Directorate of Animal Health and Production, Ministry of Agriculture, Forestry and Fisheries, Cambodia
[6]Forestry Administration, Ministry of Agriculture, Forestry and Fisheries, Cambodia
[7]Communicable Diseases Control Department, Ministry of Health, Cambodia
[8]IVPC UMR754, EPHE, Université PSL, INRAE, Université Claude Bernard Lyon 1, Lyon, France
[9]Division of Systems Virology, Department of Microbiology and Immunology, The Institute of Medical Science, The University of Tokyo, Tokyo, Japan
[10]Programme in Emerging Infectious Diseases, Duke-NUS Medical School, Singapore
[11]Institut Pasteur de Madagascar, Pasteur Network, Antananarivo, Madagascar
[12]CIRAD, UMR ASTRE, Antananarivo, Madagascar
[13]ASTRE, Université de Montpellier, CIRAD, INRAE, Montpellier, France.
[14]CIRAD, UMR ASTRE, F-34398 Montpellier, France

#correspondance to dveasna@pasteur-kh.org or etienne.simon-loriere@pasteur.fr

Supplementary Data Figure Legends

Supplementary Fig. 1: a. Geographic location of samples from which previously described sarbecovirus genomes were identified in Southeast Asia. *The viruses reported in these locations were sampled in animals confiscated by customs, and likely originate from another location. b. Map of Cambodia with provinces, highlighting in grey the Steung Treng province. In inset, detail of part of the Thala Barivat district within the Steung Treng province, showing the four karst hills (Phnom Chab Phleurng, Phnom Chhngauk, Phnom Ka Ngoak, and Phnom Kork Romeas) where sampling missions were conducted. The number and proportion (%) of positive among the samples collected in each site are noted. The maps were made with Natural Earth data (https://www.naturalearthdata.com/).

Supplementary Fig. 2: Maximum likelihood phylogenetic trees of partial RdRp gene sequences using novel and publicly available data. a, Phylogenetic tree inferred using partial RdRp gene sequences (n= 172, alignment length 294 bp) corresponding to the target of the Quan et al RT-PCR[1]. b, Phylogenetic tree inferred using partial RdRp gene sequences (n=187, alignment length 394 bp) corresponding to the target of the Chu et al RT-PCR[2]. Sequences from viruses identified in Cambodia are noted with a triangle for single signal of infection, and with a red circle for sample with signal of multiple infections. Samples with signal of multiple infections but for which the Sanger sequencing of one of the PCR products failed are further highlighted with a * symbol, and sequences obtained from high-throughput sequencing are noted with a ** symbol. Branch support values at relevant nodes are shown. Scale bar indicates nucleotide substitutions per site. The classification of coronaviruses was based on the International Committee on Taxonomy of Viruses (https://ictv.global/report/chapter/coronaviridae/coronaviridae).

Supplementary Fig. 3: Genome coverage of 42 bat SARS-CoV-2-related viruses detected in Cambodia. a, Coverage values are the combination of the sequence data obtained with the different approaches. b, Intrahost single nucleotide variant (iSNV) percentages and sequencing coverage depth are plotted relative to genome position, with a minimum read depth threshold indicated as a dashed line.
Group 1 samples were sequenced using the RshCoV amplicon primer set (AmpliSeq). Groups 2-4 were sequenced using a combination of RshCoV, RacCoV, RpuCoV, and RmaCoV AmpliSeqs, along with sequence-independent single-primer amplification (SISPA) and metatranscriptomic methods. Note: For sample RshSTT039, a coverage depth of at least 3 was used at nucleotide positions 7147-7351 and 9856-9867. The red line shows coverage generated by combining BAM files from untargeted and targeted sequencing. Data represent: Group 1 (Nextera-XT + RshCoV), Group 2 (SISPA + RshCoV + RacCoV), Group 3 (SISPA + RshCoV + RpuCoV), and Group 4 (SISPA + RshCoV + RmaCoV). Nextera-XT: Untargeted sequencing with Nextera-XT kit, SISPA: Untargeted SISPA sequencing, RshCoV, RacCoV, RpuCoV, RmaCoV: Primer sets used for amplicon sequencing for groups 1, 2, 3 and 4, respectively. Source data are provided as a Source Data file.

Supplementary Fig. 4: Phylogenetic analysis of SARS-CoV-2 related viruses. The maximum likelihood phylogeny was estimated from complete betacoronavirus genome sequences (n=75) using IQ-TREE with 1000 ultrafast bootstrap replicates. The sarbecovirus lineages genomes identified in Cambodia are colored according to groups identified based on their mosaic genome organization, spike receptor binding domain (RBD) sequence and sampling hosts. Scale bar

indicates nucleotide substitutions per site. Of note, recombination has been shown to be prevalent in the evolutionary history of sarbecoviruses, and the topology of this tree should therefore be interpreted with caution. *R*. for *Rhinolophus*, *H*. for *Hipposideros*, *L*. for *Lyroderma*, *T*. for *Taphozous*, and *M*. for *Megaderma*. Partial viral genome sequences are denoted with an asterisk (*).

Supplementary Fig. 5: Maximum likelihood phylogenetic trees of each of the genomic regions identified by recombination analysis. The positions of the 13 genomic regions analyzed are shown on a sarbecovirus genome scheme. Annotated features include ORF1ab with its nonstructural proteins (nsps), the N-terminal domain (NTD) and receptor-binding domain (RBD) of the spike (S) gene, followed by ORF3a, envelope (E), membrane (M), ORF6, ORF7a, ORF7b, ORF8, nucleocapsid (N), and ORF10. The different mosaic patterns are highlighted in colors: group 1 in orange (with lighter and darker shades in region 11 for subgroups 1a and 1b, respectively), group 2 in blue, group 3 in purple and group 4 in pink. Branch support values obtained from 1,000 ultrafast bootstrap replicates are shown on relevant nodes. Branch scale bars represent substitutions per site and the trees are midpoint rooted for clarity.

Supplementary Fig. 6: Group 2 similarity analysis. The sliding window analysis illustrates the changing patterns of sequence Identity comparing a representative group 2 virus (RacSTT345) to SARS-CoV-2, the novel group 3 or 4 viruses reported here, and representative sarbecoviruses. The analysis was performed using SimPlot v3.5.1, with a window size of 1,000 bp and a step size of 100 bp. Source data are provided as a Source Data file.

Supplementary Fig. 7: Maximum clade credibility (MCC) trees inferred for each of the 13 genomic regions and the NRA. Posterior probabilities are indicated for relevant nodes. Violin plots denote probability distributions for the estimated time to the most recent common ancestor (tMRCA) of clades of interest. The tips names of the viruses sampled in Cambodia are colored according to the group identified based on their mosaic genome organization, spike receptor binding domain (RBD) sequence and sampling hosts: group 1 in orange (with lighter and darker shades in region 11 for subgroups 1a and 1b respectively), group 2 in blue, group 3 in purple and group 4 in pink.

Supplementary Fig. 8: Maximum Clade Credibility trees inferred for the non-recombinant regions of a, group 1a and b, group 1. Node labels are posterior probabilities indicating support for the node. The violin plot indicates the 95% highest posterior density (HPD) interval distribution for the estimated time to the most recent common ancestor (tMRCA) for the clade of viruses sampled in Vietnam and the closest ancestor sampled in Cambodia. Group 1a viruses are shown in light orange, and group 1b viruses in dark orange.

Supplementary Fig. 9: Substitution rate estimates across genomic regions and virus groups. The mean and 95% highest posterior density (HPD) of the substitution rate estimates obtained with the best model tested for the 13 regions, the NRA, and the near complete genomes for group 1 and subgroup 1a are presented in black with a diamond symbol. For comparison, mean substitution rates and 95% HPD estimated in previous studies are presented in grey with a circle symbol. The SARS-CoV, hCoV-OC43, MERS-CoV, and NRR1, 2 and 3 estimates are from Boni et al.[3]. The SARS-CoV-2 estimate is from Markov et al.[4].The SARS-CoV-1-like and SARS-CoV-2-like viruses estimates are from Pekar et al.[25] Source data are provided as a Source Data file.

Supplementary Fig. 10: a. Spike proteins and ACE2 amino acid sequences phylogenies. The left maximum likelihood phylogeny was inferred using the amino acid sequences of the sarbecoviruses spikes (n = 67). Of note, recombination has been shown to be prevalent in the history of sarbecoviruses, and the topology of this tree should be interpreted with caution. The sarbecovirus lineages genomes identified in Cambodia are color-coded according to groups identified based on their mosaic genome organization, spike receptor binding domain (RBD) sequence and sampling hosts: group 1 in orange, group 2 in blue, group 3 in purple and group 4 in pink. The maximum likelihood phylogeny on the right was inferred using the ACE2 amino acid sequences (n=83) from different bat species, pangolin and human. The ACE2 sequences from which sarbecovirus sequence data was obtained are colored using the same code. The scale bars indicate amino-acid substitutions per site. The shaded boxes link the spike sequences groups to the ACE2 sequence of the bat species from which the viruses were sampled in this study. b. Comparison of ACE2 sequences within SARS-CoV-2 RBD-interaction and proximity positions, based on hACE2 numbering. Positions identified as contact residues between hACE2 and SARS-CoV-2 are highlighted with a red * symbol. When no differences were found among sequences of the same species, only one sequence is presented. The name of the species (and sample ID) corresponding to samples from which a sarbecovirus sequence was obtained in this work are highlighted using the same color code: group 1 in orange, group 2 in blue, group 3 in purple and group 4 in pink.

Supplementary Fig. 11: Comparison of the spike protein furin-like S1/S2 cleavage site sequences in the spike proteins of SARS-CoV-2, SARS-CoV-2-related viruses detected in Cambodia, and other selected representative sarbecoviruses. The alignment highlights the presence or absence of a furin-like cleavage site, as well as sequence variation in this region. Sequences are grouped by phylogenetic relatedness and colored according to virus origin or host species where applicable.

Supplementary Table Legends

Supplementary Table 1: Summary of 146 positive and 29 negative bat samples tested by Sarbecovirus RT-qPCR[5] and Pan-coronaviruses RT-PCRs[1,2], used for species identification and ACE2 sequencing.

Supplementary Table 2: List of the 50 SARS-CoV-2 related virus positive bats samples processed for sequencing. NA: Not available. RdRp: RNA-dependent RNA polymerase. The sarbecovirus RT-qPCR corresponds to the E and N genes targets from Corman et al[5].

Supplementary Table 3. Repeated molecular testing of bat samples with low viral loads. The sarbecovirus RT-qPCR corresponds to the E and N genes targets from Corman et al[5].

Supplementary Table 4. Sequence identity of group 2 (RacSTT345) compared with SARS-CoV-2 and representative sarbecovirus genomes

Supplementary Table 5. Codon sites presenting signal of selection in protein-coding genes of group 1 virus. Analysis performed in EasyCodeML v1.41 with M8 (beta and ω > 1) codon substitution model and Bayes Empirical Bayes (BEB) approach; and in HyPhy (https://www.datamonkey.org/) with MEME (Mixed Effects Model of Evolution), aBSREL (Adaptive Branch-Site Random Effects Likelihood), SLAC (Single-Likelihood Ancestor Counting), FEL (Fixed Effects Likelihood), FUBAR (Fast, Unconstrained Bayesian AppRoximation) methods.

Supplementary Table 6. Primers and probes for coronaviruses molecular detection.

Supplementary Table 7: List of publicly available sequences used in this study.

Supplementary Table 8: Evaluation of temporal signal and clock models comparisons. We tested two molecular clock models. SC: Strict clock, UCLN: uncorrelated relaxed clock with lognormal distribution. Log-marginal likelihood estimates using the generalized stepping stone sampling (GSS) model selection approach are presented for dataset using the sample dates, and the same dataset analyzed without dates, resulting in positive Bayes factors and indicating that there was temporal signal in the data.

Supplementary Table 9: Coalescent tree priors comparisons. The log marginal likelihood values were estimated using both path sampling (PS) and stepping-stone sampling (SS), and the values highlighted in bold indicate the best supported model from log Bayes factor comparisons.

References

1. Quan, P.-L. *et al.* Identification of a severe acute respiratory syndrome coronavirus-like virus in a leaf-nosed bat in Nigeria. *mBio* **1**, e00208-10 (2010).
2. Chu, D. K. W. *et al.* Avian coronavirus in wild aquatic birds. *J. Virol.* **85**, 12815–12820 (2011).
3. Boni, M. F. *et al.* Evolutionary origins of the SARS-CoV-2 sarbecovirus lineage responsible for the COVID-19 pandemic. *Nat. Microbiol.* **5**, 1408–1417 (2020).
4. Markov, P. V. *et al.* The evolution of SARS-CoV-2. *Nat. Rev. Microbiol.* **21**, 361–379 (2023).
5. Corman, V. M. *et al.* Detection of 2019 novel coronavirus (2019-nCoV) by real-time RT-PCR. *Eurosurveillance* **25**, 2000045 (2020).

Supplementary Fig. 1

a

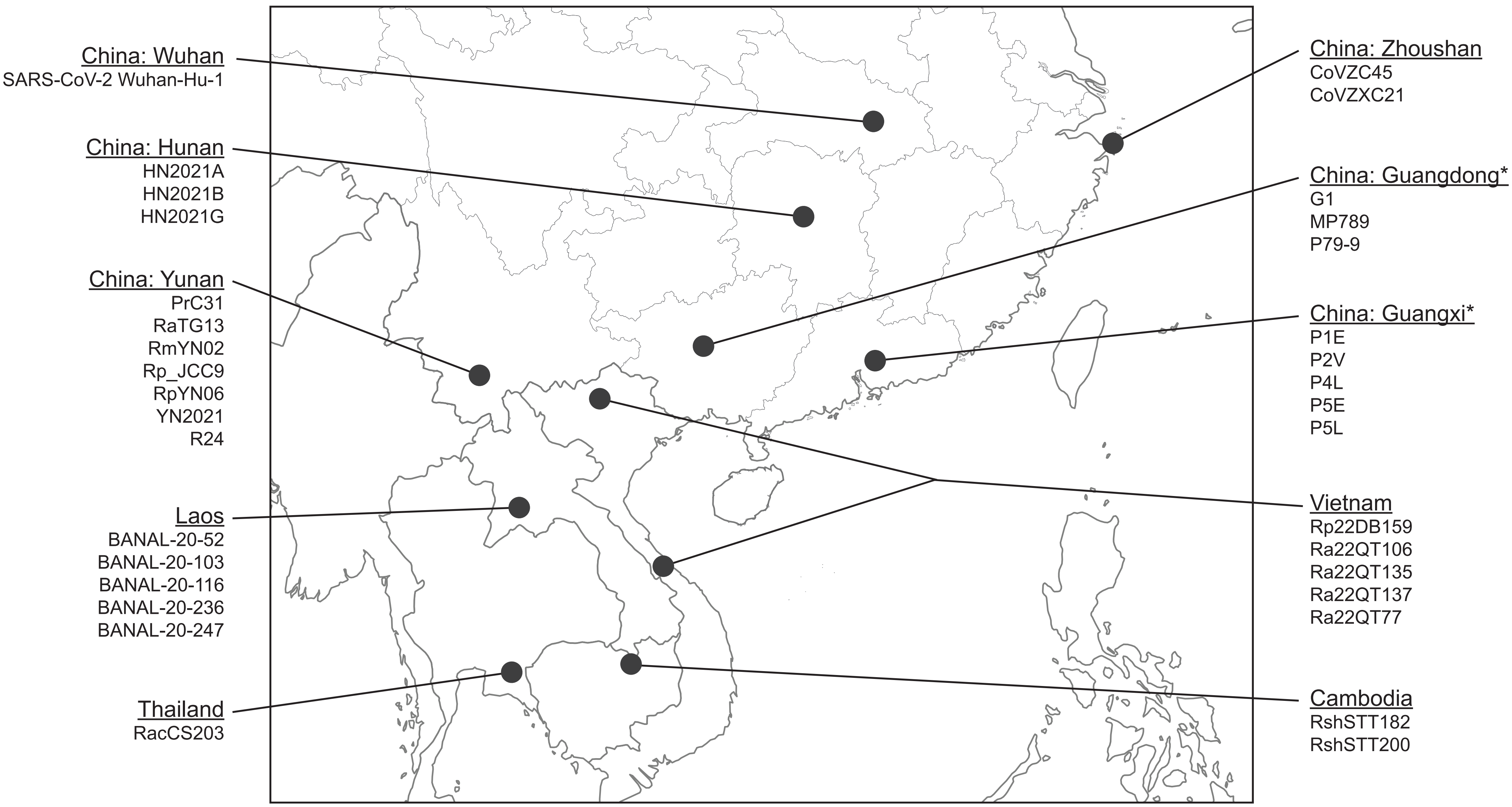

b

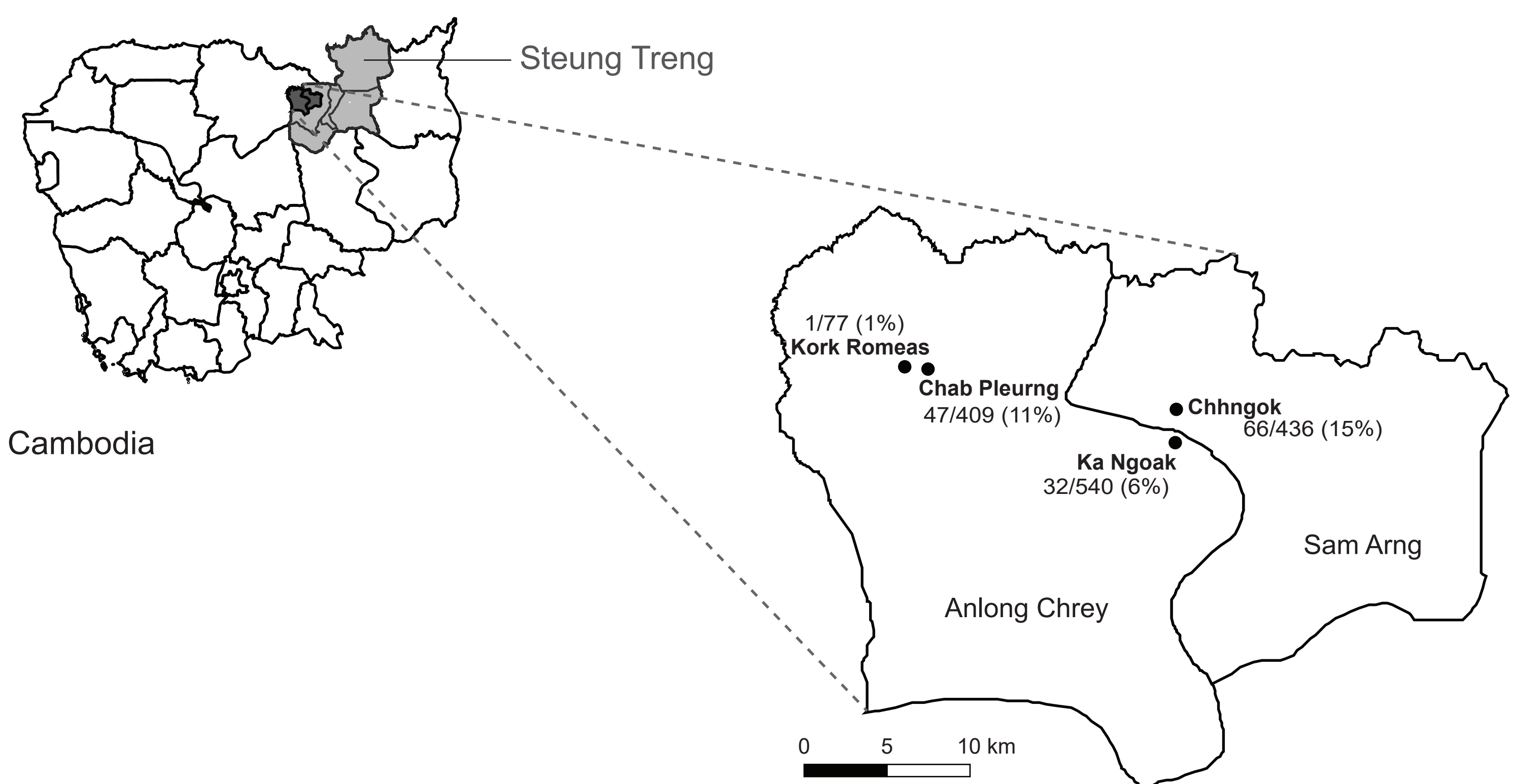

Supplementary Fig. 2

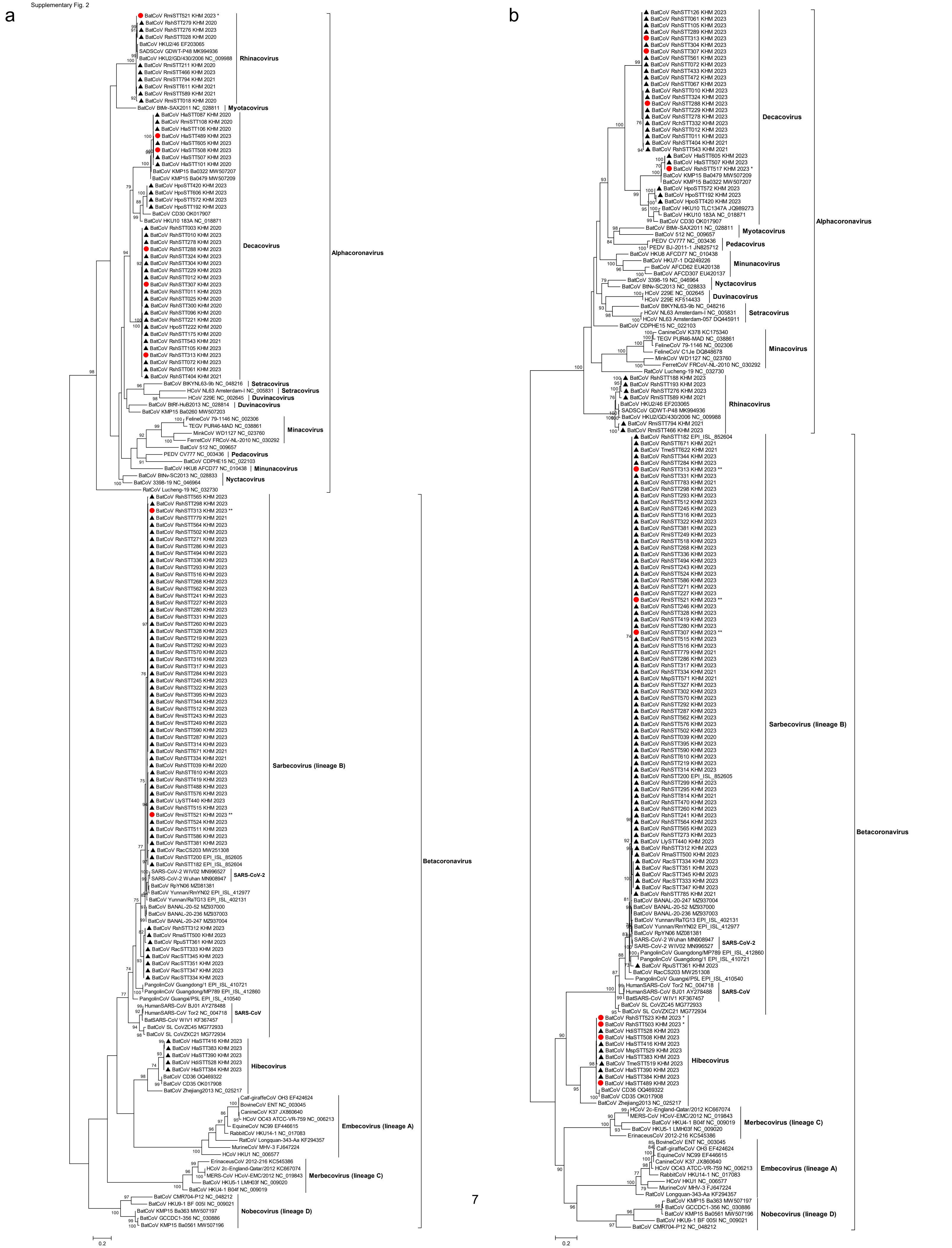

Supplementary Fig. 3

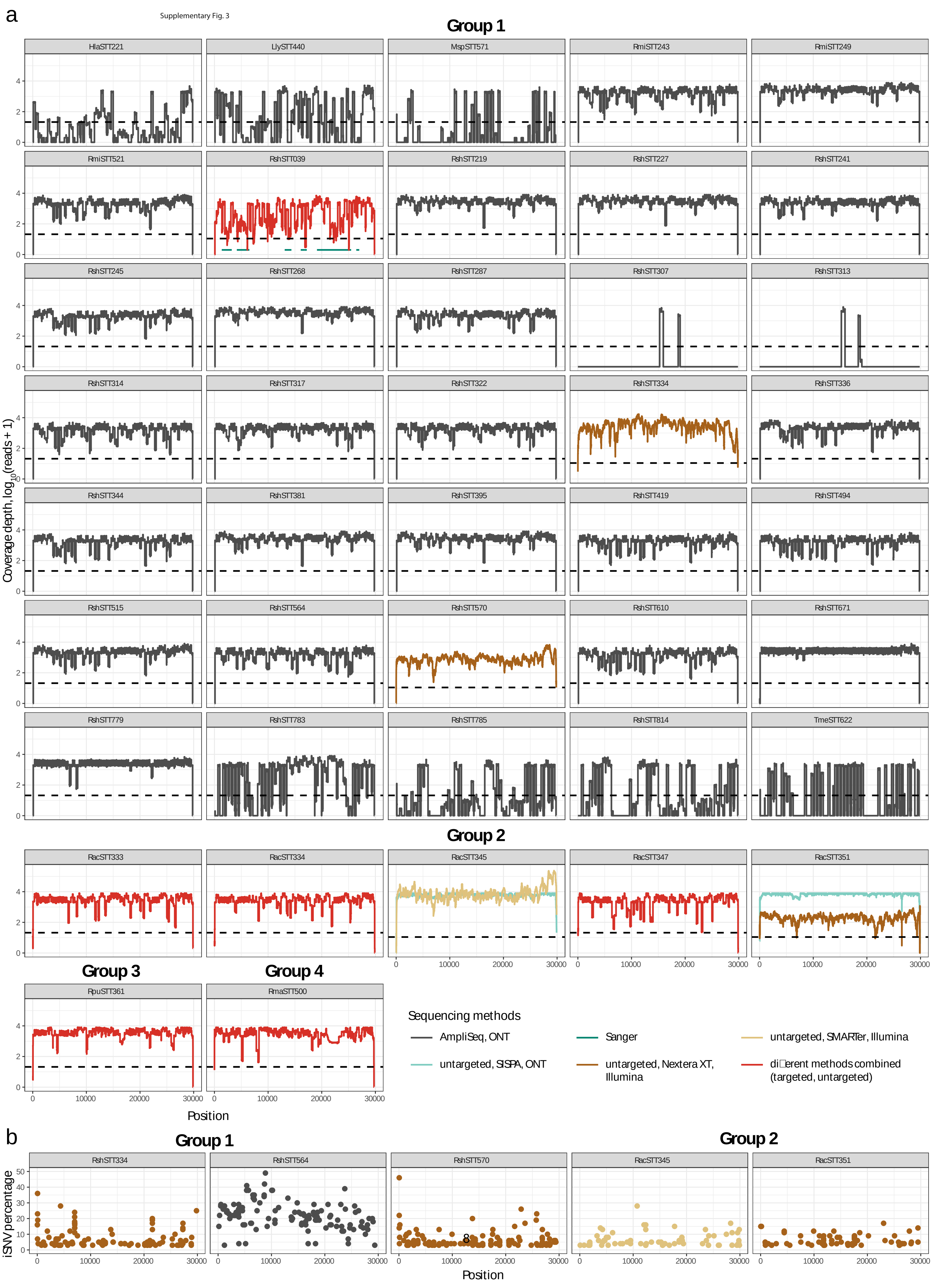

Supplementary Fig. 4

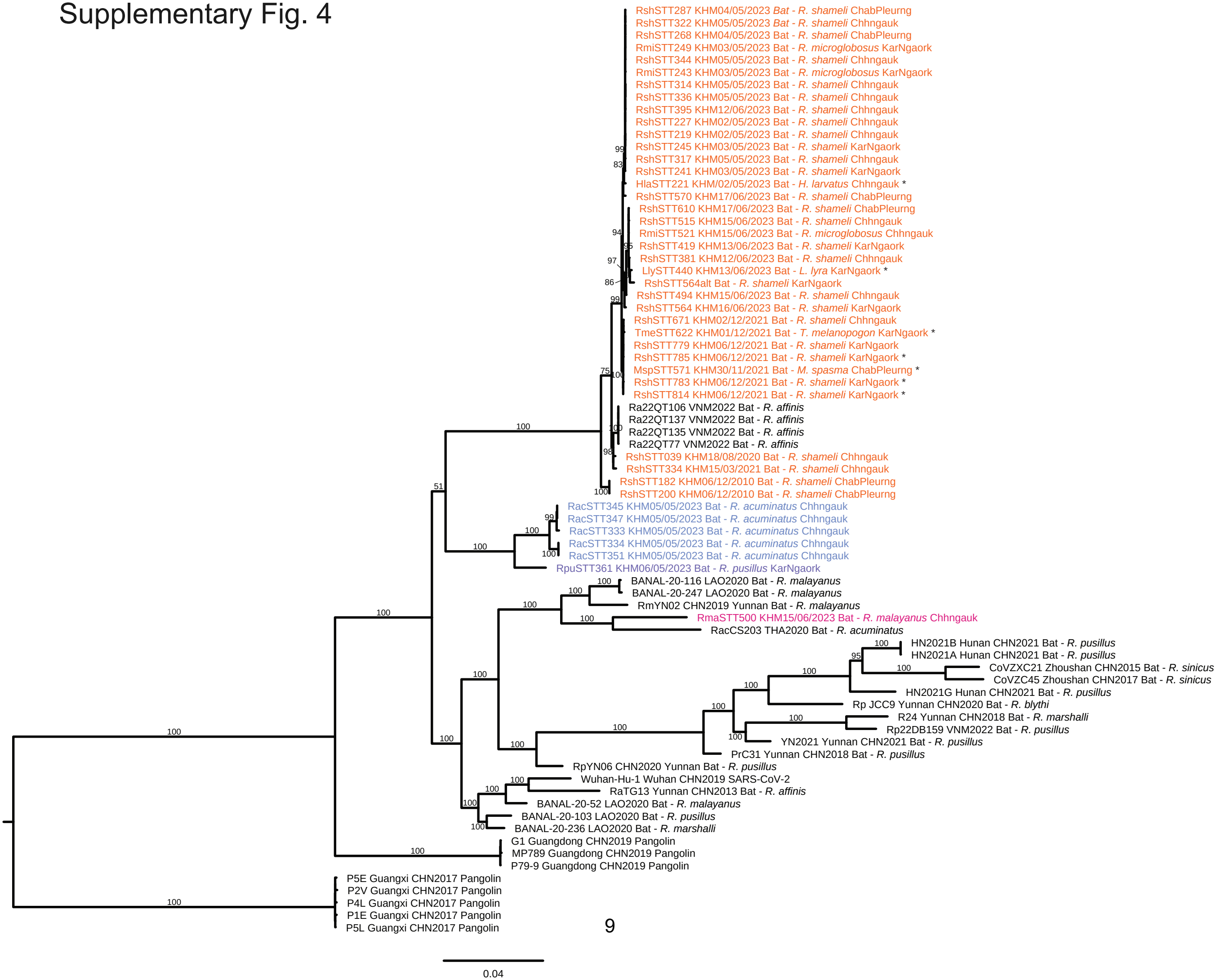

Supplementary Fig. 5

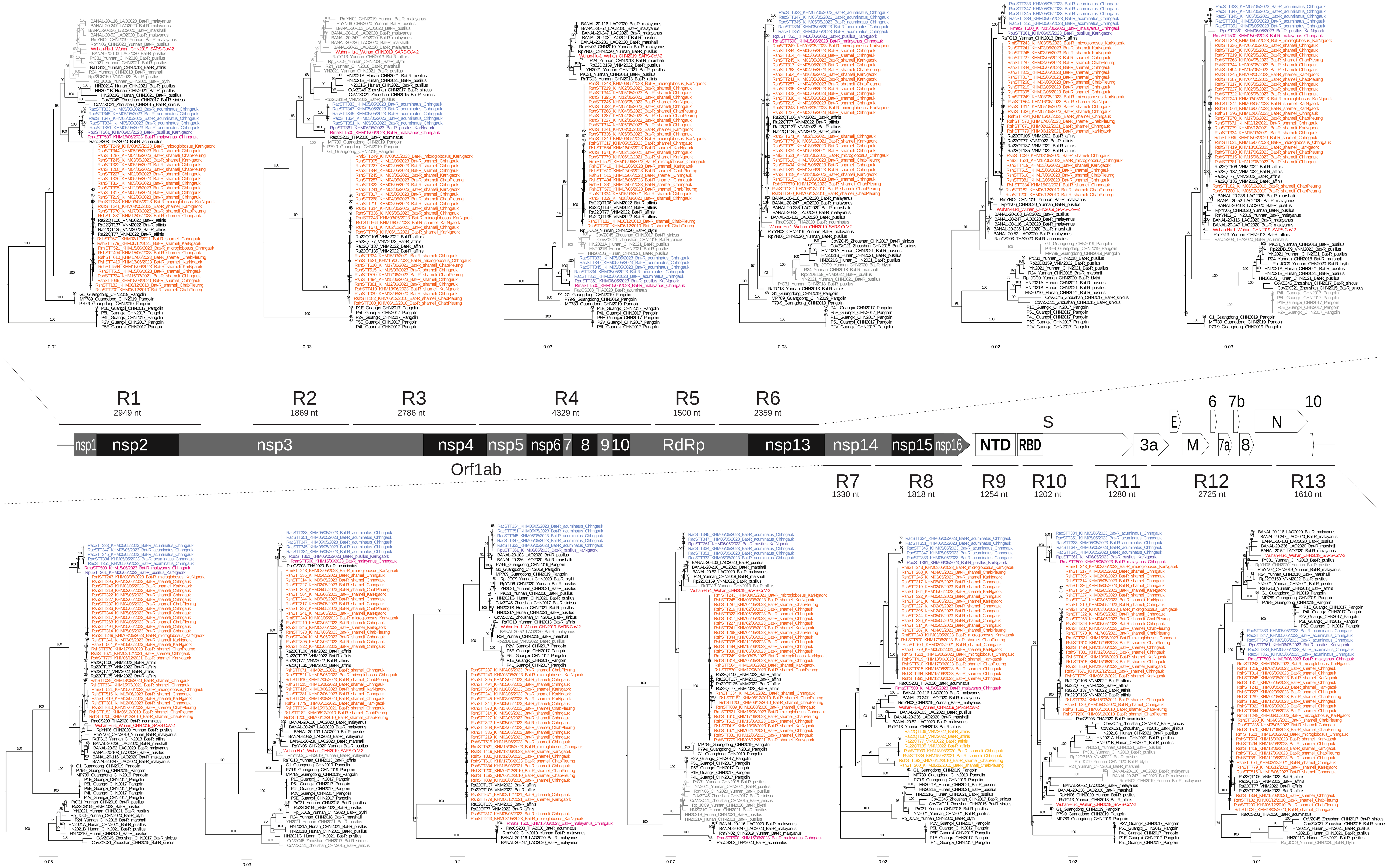

Supplementary Fig. 6

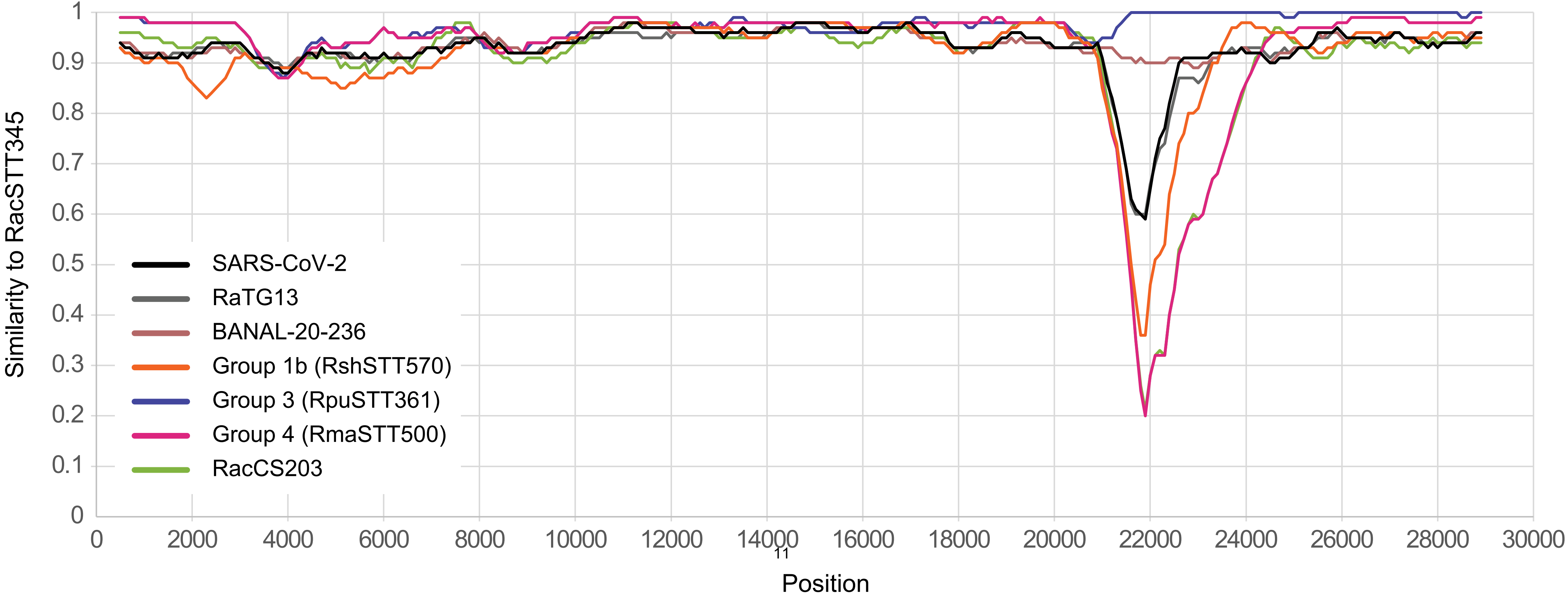

Supplementary Fig. 7

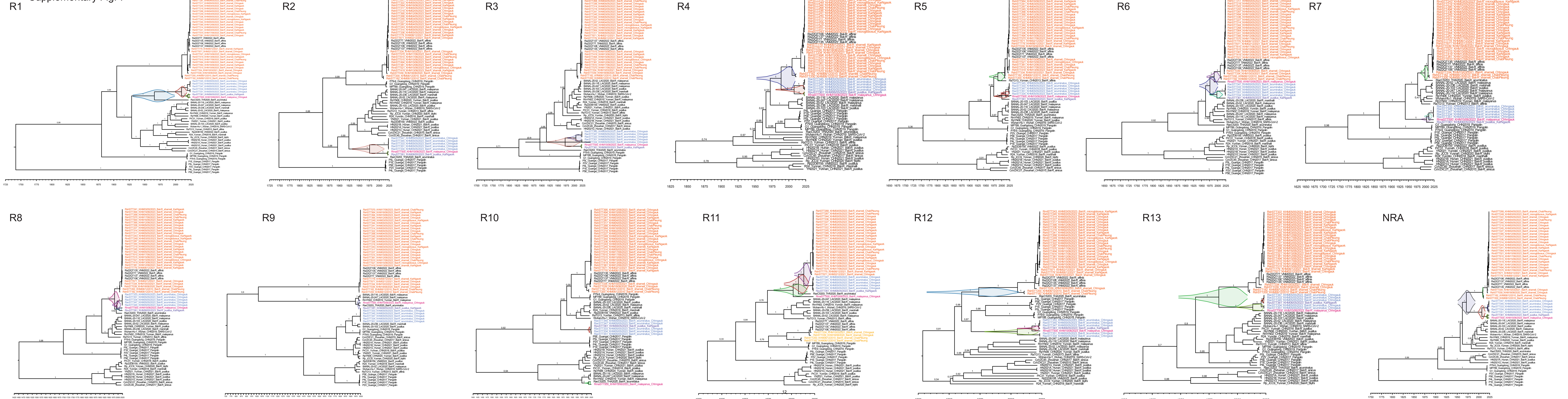

Supplementary Fig. 8

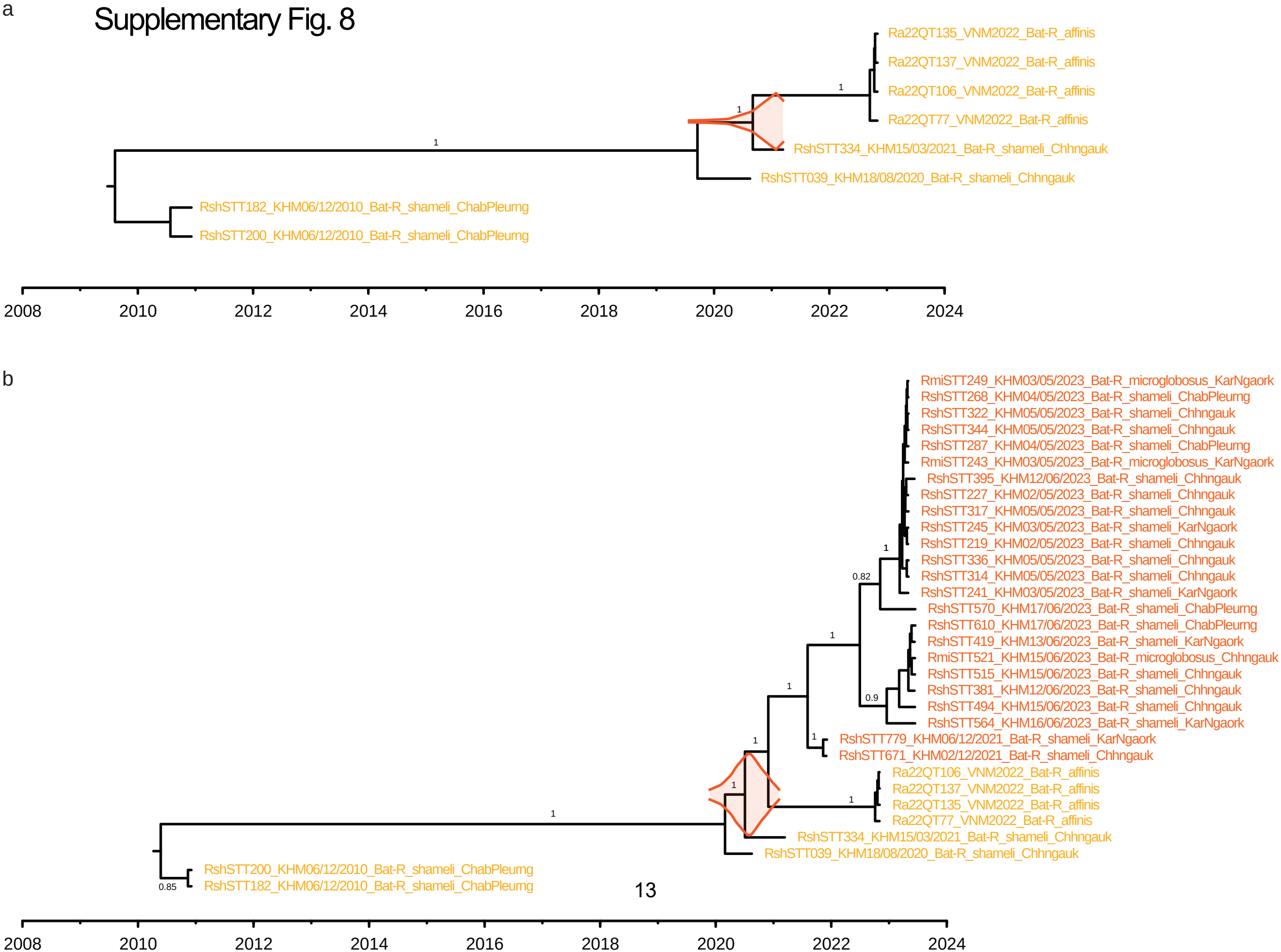

Supplementary Fig. 9

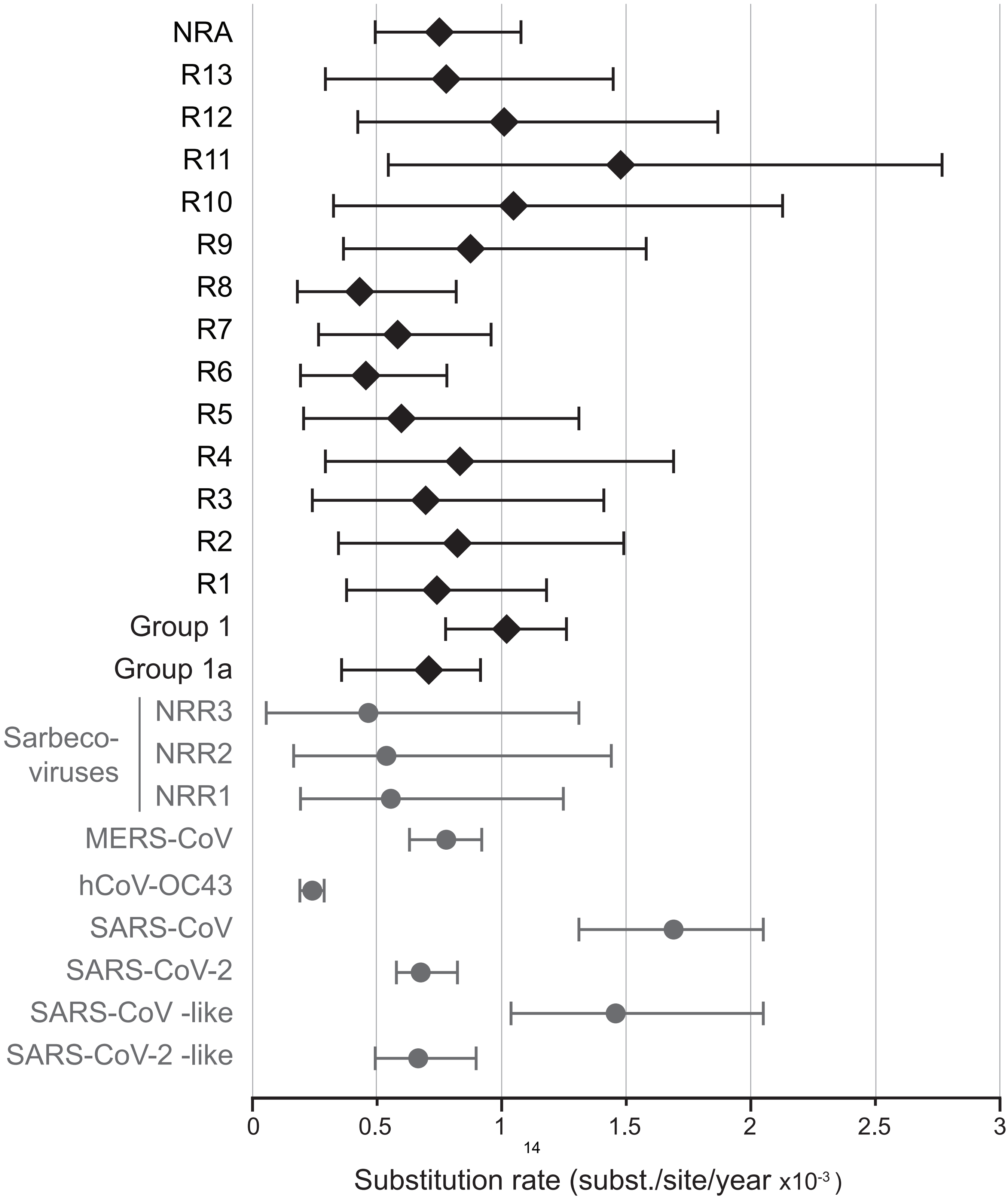

Supplementary Fig. 10

a

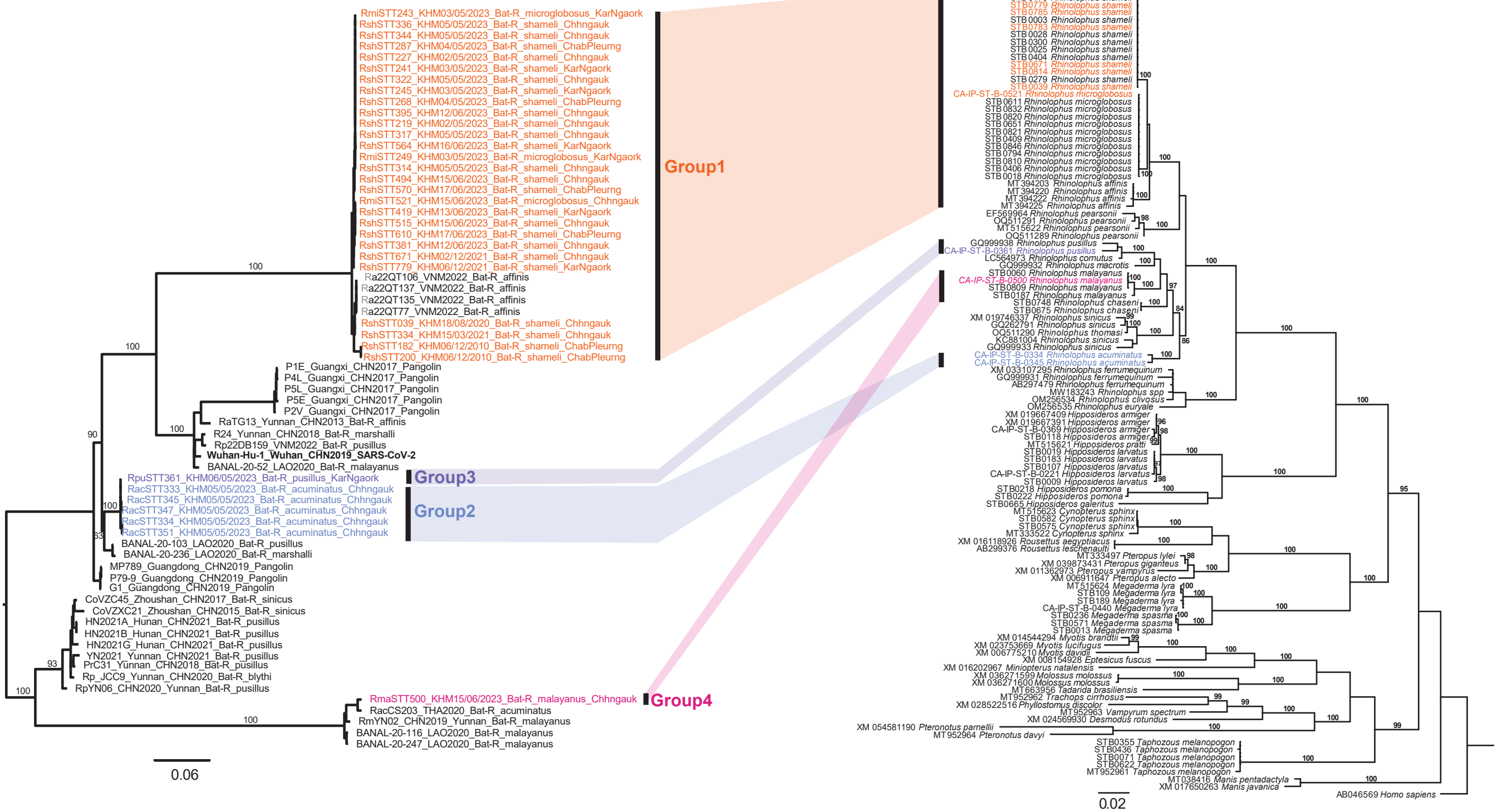

b

	24	27	28	30	31	34	35	37	38	41	42	45	79	82	83	329	330	353	354	355	357	393
R. shameli (14 sequences)	R	A	F	D	K	P	E	E	E	H	Q	L	L	N	F	N	N	K	N	D	R	R
R. microglobosus (11 sequences)	R	E	F	D	K	R	E	E	E	H	Q	L	L	N	Y	N	N	K	G	D	R	R
R. affinis (MT394225)	R	I	F	D	N	H	E	E	D	Y	Q	L	L	N	Y	N	N	K	G	D	R	R
R. affinis (MT394203)	R	I	F	D	N	R	E	E	E	Y	Q	L	L	N	Y	N	N	K	G	D	R	R
R. pearsonii (OQ511289)	Q	I	F	D	K	R	E	E	D	H	E	L	L	D	Y	N	N	K	D	D	R	R
R. pearsonii (EF569964)	R	T	F	D	K	H	E	E	D	H	E	L	L	D	Y	N	N	K	D	D	R	R
R. pearsonii (OQ511291)	R	I	F	D	K	H	E	E	D	H	E	L	L	D	Y	N	N	K	D	D	R	R
R. pusillus (GQ999938)	K	K	F	N	D	S	E	E	D	Y	Q	L	I	N	Y	N	N	K	G	D	R	R
R. pusillus (RpuSTT361)	E	K	F	N	D	S	E	E	N	Y	Q	L	I	N	Y	N	N	K	G	D	R	R
R cornutus (LC564973)	E	K	F	N	D	S	E	E	N	Y	Q	L	I	N	Y	N	N	K	G	D	R	R
R. macrotis (GQ999932)	E	K	F	D	K	S	K	E	D	Y	E	L	L	N	Y	N	K	K	G	D	R	R
R. malayanus (RmaSTT500)	K	T	F	D	T	T	E	E	D	Y	E	L	L	N	Y	N	N	R	G	D	R	R
R. malayanus (RmaSTT60)	K	T	F	D	T	T	E	E	D	Y	E	L	L	N	Y	N	N	R	G	D	R	R
R. malayanus (RmaSTT187)	K	T	F	D	K	S	K	E	D	Y	E	L	L	N	Y	N	N	K	G	D	R	R
R. chaseni (2 sequences)	A	K	F	D	T	F	E	E	N	H	Q	L	R	N	Y	N	N	K	G	D	R	R
R. sinicus (XM019746337)	R	T	F	D	E	F	E	E	N	Y	Q	L	L	N	Y	N	N	K	G	D	R	R
R. sinicus (KC881004)	E	M	F	D	K	T	K	E	D	H	Q	L	L	N	Y	N	N	K	G	D	R	R
R. sinicus (GQ999933)	E	I	F	D	K	T	K	E	D	H	Q	L	L	N	Y	N	N	K	G	D	R	R
R. sinicus (GQ262791)	L	I	F	D	E	S	E	E	N	Y	Q	L	L	N	Y	N	N	K	G	D	R	R
R. thomasi (OQ511290)	L	I	F	D	E	S	E	E	N	Y	Q	L	I	N	Y	N	N	K	G	D	R	R
R. acuminatus (RacSTT345)	E	E	F	D	K	S	E	E	D	Y	N	L	L	N	Y	N	D	K	C	D	R	R
R. acuminatus (RacSTT334)	E	A	F	D	K	S	E	E	D	Y	Q	L	L	N	Y	N	N	N	G	D	R	R
Homo sapiens	Q	T	F	D	K	H	E	E	D	Y	Q	L	L	M	Y	E	N	K	G	D	R	R
	*			*			*	*	*	*	*				*	*	*	*				*

Supplementary Fig. 11

Furin cleavage site (marked by arrowheads above positions 682–685; * at 685)

Group	Sequence	667	668	669	670	671	672	673	674	675	676	677	678	679	680	681	682	683	684	685	686	687	688	689	690	691	692	693
	SARS-CoV-2_2021-2024	G	A	G	I	C	A	S	Y	Q	T	Q	T	K	S	H	R	R	A	R	S	V	A	S	Q	S	I	I
	SARS-CoV-2_2020	G	A	G	I	C	A	S	Y	Q	T	Q	T	N	S	P	R	R	A	R	S	V	A	S	Q	S	I	I
Group1	21_SCV2r-CoVs_KHM_2023	G	A	G	I	C	A	S	Y	Q	T	Q	T	N	S	-	-	-	-	R	S	V	T	S	Q	S	I	I
	RshSTT570_2023	G	A	G	I	C	A	S	Y	Q	T	Q	T	N	S	-	-	-	-	R	S	V	T	S	Q	S	I	I
	RshSTT779_2021	G	A	G	I	C	A	S	Y	Q	T	Q	T	N	S	-	-	-	-	R	S	V	T	S	Q	S	I	I
	RshSTT671_2021	G	A	G	I	C	A	S	Y	Q	T	Q	T	N	S	-	-	-	-	R	S	V	T	S	Q	S	I	I
	RshSTT334_2021	G	A	G	I	C	A	S	Y	Q	T	Q	T	N	S	-	-	-	-	R	S	V	T	S	Q	S	I	I
	RshSTT039_2020	G	A	G	I	C	A	S	Y	Q	T	Q	T	N	S	-	-	-	-	R	S	V	T	S	Q	S	I	I
	RshSTT182/200_2010	G	A	G	I	C	A	S	Y	Q	T	Q	T	N	S	-	-	-	-	R	S	V	T	S	Q	S	I	I
	Ra22QT77_VNM2022	G	A	G	I	C	A	S	Y	Q	T	Q	T	N	S	-	-	-	-	R	S	V	T	S	Q	S	I	I
Group2	RacSTT333_2023	G	A	G	I	C	A	S	Y	Q	T	Q	I	K	S	-	-	-	-	R	S	V	T	S	Q	S	I	I
	RacSTT334_2023	G	A	G	I	C	A	S	Y	Q	T	Q	I	K	S	-	-	-	-	R	S	V	T	S	Q	S	I	I
	RacSTT345_2023	G	A	G	I	C	A	S	Y	Q	T	Q	I	K	S	-	-	-	-	R	S	V	T	S	Q	S	I	I
	RacSTT345_2023	G	A	G	I	C	A	S	Y	Q	T	Q	I	K	S	-	-	-	-	R	S	V	T	S	Q	S	I	I
	RacSTT351_2023	G	A	G	I	C	A	S	Y	Q	T	Q	I	K	S	-	-	-	-	R	S	V	T	S	Q	S	I	I
Group3	RpuSTT361_2023	G	A	G	I	C	A	S	Y	Q	T	Q	I	K	S	-	-	-	-	R	S	V	T	S	Q	S	I	I
	Ra22DB159_VNM2022	G	A	G	I	C	A	S	Y	Q	T	Q	T	N	S	-	-	-	-	R	S	V	A	S	Q	S	I	I
	BANAL-20-52_LAO2020	G	A	G	I	C	A	S	Y	Q	T	Q	T	N	S	-	-	-	-	R	S	V	A	S	Q	S	I	I
	BANAL-20-236_LAO2020	G	A	G	I	C	A	S	Y	Q	T	Q	T	N	S	-	-	-	-	R	S	V	A	S	Q	S	I	I
	RaTG13_CHN2013	G	A	G	I	C	A	S	Y	Q	T	Q	T	N	S	-	-	-	-	R	S	V	A	S	Q	S	I	I
	Pangolin/GD1_CHN2020	G	A	G	I	C	A	S	Y	Q	T	Q	T	N	S	-	-	-	-	R	S	V	S	S	Q	A	I	I
Group4	RmaSTT500_2023	G	A	G	V	C	A	S	Y	-	-	-	-	N	S	P	-	V	A	R	-	V	G	T	N	S	I	I
	RacCS203_THA2020	G	A	G	V	C	A	S	Y	-	-	-	-	N	S	P	-	V	A	R	-	V	G	T	N	S	I	I
	RmYN02_CHN2019	G	A	G	V	C	A	S	Y	-	-	-	-	N	S	P	-	A	A	R	-	V	G	T	N	S	I	I
	BANAL-20-247_LAO2020	G	A	G	V	C	A	S	Y	-	-	-	-	N	S	P	-	A	A	R	-	V	G	T	N	S	I	I
	RpYN06_CHN2020	G	A	G	I	C	A	S	Y	H	A	A	S	-	-	-	-	I	L	R	S	T	S	Q	K	A	I	V
	SARS-CoV_Human/GZ02_CHN2002	G	A	G	I	C	A	S	Y	H	T	V	S	-	-	-	-	L	L	R	S	T	S	Q	K	S	I	V
	MERS-CoV_Human_SAU2016	Q	S	L	C	A	L	P	D	T	P	S	T	L	T	P	R	S	V	R	S	V	P	G	E	M	R	L

Supplementary Table 1. Summary of 146 positive and 29 negative bat samples tested by Sarbecovirus RT-qPCR and Pan-coronaviruses RT-PCRs used for species identification and ACE2 sequencing

Sample ID	Genus/Species	Sex	Age-Status	Site of sample collection	Date of sampling	Sarbecovirus RT-qPCR (19)		Pan-CoV RT-PCR		ID GenBank of partial RdRp gene	Classification based on partial RdRp sequence	COI GenBank ID (ENA)	ACE2 GenBank ID (ENA)
						E-gene Ct value	N-gene Ct value	Quan et al. (20)	Chu et al. (21)				
RshSTT039	*Rhinolophus shameli*	Male	Mature	Phnom Chhngauk	2020-08-18	29.22	27.33	Negative	NA	PQ305308	BetaCoV-Sarbecovirus	OZ248192	OZ248119
RshSTT334	*Rhinolophus shameli*	Male	Immature	Phnom Chhngauk	2021-03-15	24.35	23.5	Negative	NA	PQ305309	BetaCoV-Sarbecovirus	OZ248193	NA
MspSTT571	*Megaderma spasma*	Female	Juvenile	Phnom Chab Phleurng	2021-11-30	38.4	38.27	Negative	NA	PQ305310	BetaCoV-Sarbecovirus	OZ248194	OZ248090
TmeSTT622	*Taphozous melanopogon*	Female	Parous	Phnom Ka Ngoak	2021-12-01	37.99	36.38	Negative	NA	PQ305311	BetaCoV-Sarbecovirus	OZ248195	OZ248094
RshSTT671	*Rhinolophus shameli*	Male	Mature	Phnom Chhngauk	2021-12-02	21.22	19.21	Positive	NA	PQ305312	BetaCoV-Sarbecovirus	OZ248196	OZ248124
RshSTT779	*Rhinolophus shameli*	Male	Mature	Phnom Ka Ngoak	2021-12-06	23.98	22.23	Negative	NA	PQ305313	BetaCoV-Sarbecovirus	OZ248197	OZ248125
RshSTT783	*Rhinolophus shameli*	Female	Parous	Phnom Ka Ngoak	2021-12-06	34.38	33.34	Negative	NA	PQ305314	BetaCoV-Sarbecovirus	OZ248198	OZ248126
RshSTT785	*Rhinolophus shameli*	Male	Mature	Phnom Ka Ngoak	2021-12-06	40.63	36.77	Negative	NA	NA	BetaCoV-Sarbecovirus	OZ248199	OZ248127
RshSTT814	*Rhinolophus shameli*	Male	Mature	Phnom Ka Ngoak	2021-12-06	36.77	37.03	Negative	NA	PQ305315	BetaCoV-Sarbecovirus	OZ248200	OZ248128
RshSTT003	*Rhinolophus shameli*	Male	Mature	Phnom Chhngauk	2020-08-18	Negative	Negative	Positive	NA	PQ305323	AlphaCoV-Decacovirus	OZ248136	OZ248116
RmiSTT018	*Rhinolophus microglobosus*	NA	NA	Phnom Chhngauk	2020-08-18	Negative	Negative	Positive	NA	PQ305324	AlphaCoV-Rhinacovirus	OZ248201	OZ248104
RshSTT025	*Rhinolophus shameli*	Female	Nulliparous	Phnom Chhngauk	2020-08-18	Negative	Negative	Positive	NA	PQ305325	AlphaCoV-Decacovirus	OZ248137	OZ248117
RshSTT028	*Rhinolophus shameli*	Female	Nulliparous	Phnom Chhngauk	2020-08-18	Negative	Negative	Positive	NA	PQ305326	AlphaCoV-Rhinacovirus	OZ248138	OZ248118
HlaSTT087	*Hipposideros larvatus*	Female	Nulliparous	Phnom Chab Phleurng	2020-08-19	Negative	Negative	Positive	NA	PQ305327	AlphaCoV-Decacovirus	OZ248202	NA
RshSTT096	*Rhinolophus shameli*	Female	Nulliparous	Phnom Chab Phleurng	2020-08-19	Negative	Negative	Positive	NA	PQ305328	AlphaCoV-Decacovirus	OZ248203	NA
HlaSTT101	*Hipposideros larvatus*	Male	Mature	Phnom Chab Phleurng	2020-08-19	Negative	Negative	Positive	NA	PQ305329	AlphaCoV-Decacovirus	OZ248204	NA
HlaSTT106	*Hipposideros larvatus*	Male	Mature	Phnom Chhngauk	2020-08-20	Negative	Negative	Positive	NA	PQ305330	AlphaCoV-Decacovirus	OZ248205	NA
RmiSTT108	*Rhinolophus microglobosus*	Female	Nulliparous	Phnom Chhngauk	2020-08-20	Negative	Negative	Positive	NA	PQ305331	AlphaCoV-Decacovirus	OZ248139	NA
RshSTT175	*Rhinolophus shameli*	Female	Nulliparous	Phnom Chhngauk	2020-10-13	Negative	Negative	Positive	NA	PQ305332	AlphaCoV-Decacovirus	OZ248140	OZ248120
RmiSTT211	*Rhinolophus microglobosus*	Male	Immature	Phnom Chhngauk	2020-10-15	Negative	Negative	Positive	NA	PQ305333	AlphaCoV-Rhinacovirus	OZ248206	NA
RshSTT221	*Rhinolophus shameli*	Female	Nulliparous	Phnom Chhngauk	2020-10-15	Negative	Negative	Positive	NA	PQ305334	AlphaCoV-Decacovirus	OZ248141	NA
HpoSTT222	*Hipposideros pomona*	Female	Nulliparous	Phnom Chhngauk	2020-10-15	Negative	Negative	Positive	NA	PQ305335	AlphaCoV-Decacovirus	OZ248142	OZ248084
RshSTT279	*Rhinolophus shameli*	Male	mature	Phnom Ka Ngoak	2020-10-16	Negative	Negative	Positive	NA	PQ305336	AlphaCoV-Rhinacovirus	OZ248143	OZ248121
RshSTT300	*Rhinolophus shameli*	Female	Nulliparous	Phnom Chhngauk	2020-10-17	Negative	Negative	Positive	NA	PQ305337	AlphaCoV-Decacovirus	OZ248144	OZ248122
RshSTT404	*Rhinolophus shameli*	Male	Mature	Phnom Ka Ngoak	2021-03-17	Negative	Negative	Positive	NA	PQ305316	AlphaCoV-Decacovirus	OZ248145	OZ248123
RshSTT543	*Rhinolophus shameli*	Male	Mature	Phnom Kork Romeas	2021-09-03	Negative	Negative	Positive	NA	PQ305317	AlphaCoV-Decacovirus	OZ248207	NA
RmiSTT589	*Rhinolophus microglobosus*	Male	Mature	Phnom Ka Ngoak	2021-12-01	Negative	Negative	Positive	NA	PQ305318	AlphaCoV-Rhinacovirus	OZ248208	NA
RmiSTT611	*Rhinolophus microglobosus*	Male	Mature	Phnom Ka Ngoak	2021-12-01	Negative	Negative	Positive	NA	PQ305338	AlphaCoV-Rhinacovirus	OZ248146	OZ248107
RmiSTT794	*Rhinolophus microglobosus*	Male	Immature	Phnom Ka Ngoak	2021-12-06	Negative	Negative	Positive	NA	PQ305319	AlphaCoV-Rhinacovirus	OZ248209	OZ248109
HlaSTT009	*Hipposideros larvatus*	Female	Nulliparous	Phnom Chhngauk	2020-08-18	Negative	Negative	Negative	NA	NA	NA	OZ248147	OZ248078
MspSTT013	*Megaderma spasma*	Male	Mature	Phnom Chhngauk	2020-08-18	Negative	Negative	Negative	NA	NA	NA	OZ248148	OZ248088
HlaSTT019	*Hipposideros larvatus*	Female	Nulliparous	Phnom Chhngauk	2020-08-18	Negative	Negative	Negative	NA	NA	NA	OZ248149	OZ248079
RmaSTT060	*Rhinolophus malayanus*	Male	NA	Phnom Kork Romeas	2020-08-19	Negative	Negative	Negative	NA	NA	NA	OZ248150	OZ248097
TmeSTT071	*Taphozous melanopogon*	Female	Nulliparous	Phnom Kork Romeas	2020-08-19	Negative	Negative	Negative	NA	NA	NA	OZ248151	OZ248091
HlaSTT107	*Hipposideros larvatus*	Male	Mature	Phnom Chhngauk	2020-08-20	Negative	Negative	Negative	NA	NA	NA	OZ248152	OZ248080
LlySTT109	*Lyroderma lyra*	Female	Nulliparous	Phnom Chhngauk	2020-08-20	Negative	Negative	Negative	NA	NA	NA	OZ248153	OZ248085
HarSTT118	*Hipposideros armiger*	Male	Mature	Phnom Ka Ngoak	2020-08-21	Negative	Negative	Negative	NA	NA	NA	OZ248154	OZ248075
HlaSTT183	*Hipposideros larvatus*	Female	Nulliparous	Phnom Chhngauk	2020-10-13	Negative	Negative	Negative	NA	NA	NA	OZ248155	OZ248081
RmaSTT187	*Rhinolophus malayanus*	Male	Mature	Phnom Chhngauk	2020-10-13	Negative	Negative	Negative	NA	NA	NA	OZ248156	OZ248098
LlySTT189	*Lyroderma lyra*	Female	Nulliparous	Phnom Chhngauk	2020-10-13	Negative	Negative	Negative	NA	NA	NA	OZ248157	OZ248086
HpoSTT218	*Hipposideros pomona*	Male	mature	Phnom Chhngauk	2020-10-15	Negative	Negative	Negative	NA	NA	NA	OZ248158	OZ248083
MspSTT236	*Megaderma spasma*	Female	Nulliparous	Phnom Chhngauk	2020-10-15	Negative	Negative	Negative	NA	NA	NA	OZ248159	OZ248089
TmeSTT355	*Taphozous melanopogon*	Female	Nulliparous	Phnom Chhngauk	2021-03-15	Negative	Negative	Negative	NA	NA	NA	OZ248160	OZ248092
RmiSTT406	*Rhinolophus microglobosus*	Male	Mature	Phnom Ka Ngoak	2021-03-17	Negative	Negative	Negative	NA	NA	NA	OZ248161	OZ248105
RmiSTT409	*Rhinolophus microglobosus*	Male	Mature	Phnom Ka Ngoak	2021-03-17	Negative	Negative	Negative	NA	NA	NA	OZ248162	OZ248106
TmeSTT436	*Taphozous melanopogon*	Male	Mature	Phnom Chhngauk	2021-08-31	Negative	Negative	Negative	NA	NA	NA	OZ248163	OZ248093
CspSTT575	*Cynopterus sphinx*	Male	Immature	Phnom Chab Phleurng	2021-11-30	Negative	Negative	Negative	NA	NA	NA	OZ248164	OZ248074
CspSTT582	*Cynopterus sphinx*	Female	Parous	Phnom Chab Phleurng	2021-11-30	Negative	Negative	Negative	NA	NA	NA	OZ248165	OZ248073
RmiSTT651	*Rhinolophus microglobosus*	Female	Parous	Phnom Chhngauk	2021-12-02	Negative	Negative	Negative	NA	NA	NA	OZ248166	OZ248108
HgaSTT665	*Hipposideros galeritus*	Male	Immature	Phnom Chhngauk	2021-12-02	Negative	Negative	Negative	NA	NA	NA	OZ248167	OZ248077
RchSTT675	*Rhinolophus chaseni*	Female	Parous	Phnom Kork Romeas	2021-12-04	Negative	Negative	Negative	NA	NA	NA	OZ248168	OZ248095
RchSTT748	*Rhinolophus chaseni*	Female	Parous	Phnom Chab Phleurng	2021-12-05	Negative	Negative	Negative	NA	NA	NA	OZ248169	OZ248096
RmaSTT809	*Rhinolophus malayanus*	Male	Mature	Phnom Ka Ngoak	2021-12-06	Negative	Negative	Negative	NA	NA	NA	OZ248170	OZ248099
RmiSTT810	*Rhinolophus microglobosus*	Female	Parous	Phnom Ka Ngoak	2021-12-06	Negative	Negative	Negative	NA	NA	NA	OZ248171	OZ248110
RmiSTT820	*Rhinolophus microglobosus*	Female	Nulliparous	Phnom Ka Ngoak	2021-12-06	Negative	Negative	Negative	NA	NA	NA	OZ248172	OZ248111
RmiSTT821	*Rhinolophus microglobosus*	Female	Parous	Phnom Ka Ngoak	2021-12-06	Negative	Negative	Negative	NA	NA	NA	OZ248173	OZ248112
RmiSTT832	*Rhinolophus microglobosus*	Male	Mature	Phnom Ka Ngoak	2021-12-06	Negative	Negative	Negative	NA	NA	NA	OZ248174	OZ248113
RmiSTT846	*Rhinolophus microglobosus*	Male	Mature	Phnom Ka Ngoak	2021-12-06	Negative	Negative	Negative	NA	NA	NA	OZ248175	OZ248114
RshSTT219	*Rhinolophus shameli*	Female	Nulliparous	Phnom Chhngauk	2023-05-02	24.29	24.02	Positive	Positive	OZ250989/OZ250910	BetaCoV-Sarbecovirus	OZ248210	NA
HlaSTT221	*Hipposideros larvatus*	Female	Lactating	Phnom Chhngauk	2023-05-02	39.25	38.74	Negative	Negative	NA	Sarbecovirus positive by RT-qPCR	OZ248211	OZ248082
RshSTT227	*Rhinolophus shameli*	Female	Nulliparous	Phnom Chhngauk	2023-05-02	23.29	23.17	Positive	Positive	OZ250990/OZ250911	BetaCoV-Sarbecovirus	OZ248212	NA
RshSTT241	*Rhinolophus shameli*	Male	Immature	Phnom Ka Ngoak	2023-05-03	28.08	27.05	Positive	Positive	OZ250992/OZ250913	BetaCoV-Sarbecovirus	OZ248213	NA
RmiSTT243	*Rhinolophus microglobosus*	Male	Immature	Phnom Ka Ngoak	2023-05-03	17.86	15.76	Positive	Positive	OZ250993/OZ250914	BetaCoV-Sarbecovirus	OZ248176	NA
RshSTT245	*Rhinolophus shameli*	Male	Immature	Phnom Ka Ngoak	2023-05-03	17.05	16.86	Positive	Positive	OZ250994/OZ250915	BetaCoV-Sarbecovirus	OZ248214	NA
RshSTT246	*Rhinolophus shameli*	Female	Parous	Phnom Ka Ngoak	2023-05-03	35.9	36.05	Negative	Positive	OZ250884	BetaCoV-Sarbecovirus	OZ248215	NA
RmiSTT249	*Rhinolophus microglobosus*	Male	Immature	Phnom Ka Ngoak	2023-05-03	23.04	22.06	Positive	Positive	OZ250995/OZ250916	BetaCoV-Sarbecovirus	OZ248177	NA
RmiSTT252	*Rhinolophus microglobosus*	Male	Mature	Phnom Ka Ngoak	2023-05-03	39.79	37.47	Negative	Negative	NA	Sarbecovirus positive by RT-qPCR	OZ248178	NA
RshSTT260	*Rhinolophus shameli*	Female	Parous	Phnom Ka Ngoak	2023-05-03	27.17	25.98	Positive	Positive	OZ250996/OZ250917	BetaCoV-Sarbecovirus	OZ248216	NA
RshSTT265	*Rhinolophus shameli*	Male	Mature	Phnom Chab Phleurng	2023-05-04	37.47	37.68	Negative	Negative	NA	Sarbecovirus positive by RT-qPCR	OZ248217	NA
RshSTT268	*Rhinolophus shameli*	Male	Mature	Phnom Chab Phleurng	2023-05-04	21.46	21.12	Positive	Positive	OZ250997/OZ250918	BetaCoV-Sarbecovirus	OZ248218	NA
RshSTT271	*Rhinolophus shameli*	Male	Mature	Phnom Chab Phleurng	2023-05-04	22.39	22.2	Positive	Positive	OZ250998/OZ250919	BetaCoV-Sarbecovirus	OZ248219	NA
RshSTT273	*Rhinolophus shameli*	Male	Mature	Phnom Chab Phleurng	2023-05-04	28.49	26.81	Negative	Positive	OZ250885	BetaCoV-Sarbecovirus	OZ248220	NA
RshSTT280	*Rhinolophus shameli*	Male	Mature	Phnom Chab Phleurng	2023-05-04	25.29	24.13	Positive	Positive	OZ251001/OZ250922	BetaCoV-Sarbecovirus	OZ248221	NA
RshSTT284	*Rhinolophus shameli*	Male	Mature	Phnom Chab Phleurng	2023-05-04	34.09	33.84	Positive	Positive	OZ251002/OZ250923	BetaCoV-Sarbecovirus	OZ248222	NA
RshSTT286	*Rhinolophus shameli*	Male	Mature	Phnom Chab Phleurng	2023-05-04	24.03	22.16	Positive	Positive	OZ251003/OZ250924	BetaCoV-Sarbecovirus	OZ248223	NA
RshSTT287	*Rhinolophus shameli*	Male	Mature	Phnom Chab Phleurng	2023-05-04	23.5	21.45	Positive	Positive	OZ251004/OZ250925	BetaCoV-Sarbecovirus	OZ248224	NA
RshSTT288	*Rhinolophus shameli*	Male	Mature	Phnom Chab Phleurng	2023-05-04	37.12	36.22	Positive	Positive	OZ251005/OZ250926	BetaCoV-sarbecovirus & AlphaCoV-Decacovirus	OZ248225	NA
RshSTT290	*Rhinolophus shameli*	Male	Mature	Phnom Chab Phleurng	2023-05-04	38.61	37.65	Negative	Negative	NA	Sarbecovirus positive by RT-qPCR	OZ248226	NA
RshSTT292	*Rhinolophus shameli*	Male	Immature	Phnom Chab Phleurng	2023-05-04	31.37	30.65	Positive	Positive	OZ251006/OZ250927	BetaCoV-Sarbecovirus	OZ248227	NA
RshSTT293	*Rhinolophus shameli*	Male	Immature	Phnom Chab Phleurng	2023-05-04	28.68	29.46	Positive	Positive	OZ251007/OZ250928	BetaCoV-Sarbecovirus	OZ248228	NA
RshSTT295	*Rhinolophus shameli*	Male	Mature	Phnom Chab Phleurng	2023-05-04	32.29	31.77	Negative	Positive	OZ250887	BetaCoV-Sarbecovirus	OZ248229	NA
RshSTT296	*Rhinolophus shameli*	Female	Parous	Phnom Chab Phleurng	2023-05-04	40.73	36.29	Negative	Negative	NA	Sarbecovirus positive by RT-qPCR	OZ248230	NA
RshSTT298	*Rhinolophus shameli*	Female	Parous	Phnom Chab Phleurng	2023-05-04	34.28	34.18	Positive	Positive	OZ251008/OZ250929	BetaCoV-Sarbecovirus	OZ248231	NA
RshSTT299	*Rhinolophus shameli*	Male	Mature	Phnom Chab Phleurng	2023-05-04	37.65	35.81	Negative	Positive	OZ250888	BetaCoV-Sarbecovirus	OZ248232	NA
RshSTT302	*Rhinolophus shameli*	Male	Mature	Phnom Chab Phleurng	2023-05-04	32.64	30.3	Negative	Positive	OZ250889	BetaCoV-Sarbecovirus	OZ248233	NA
RshSTT307	*Rhinolophus shameli*	Female	Parous	Phnom Chab Phleurng	2023-05-04	36.13	33.92	Positive	Positive	OZ251010/OZ250931	BetaCoV-sarbecovirus & AlphaCoV-Decacovirus	OZ248234	NA
RshSTT312	*Rhinolophus shameli*	Female	Parous	Phnom Chhngauk	2023-05-05	32.94	34.57	Positive	Positive	OZ251011/OZ250932	BetaCoV-Sarbecovirus	OZ248235	NA
RshSTT313	*Rhinolophus shameli*	Female	Parous	Phnom Chhngauk	2023-05-05	36.65	36.33	Positive	Positive	OZ251012/OZ250933	BetaCoV-sarbecovirus & AlphaCoV-Decacovirus	OZ248236	NA
RshSTT314	*Rhinolophus shameli*	Male	Mature	Phnom Chhngauk	2023-05-05	20.87	18.09	Positive	Positive	OZ251013/OZ250934	BetaCoV-Sarbecovirus	OZ248237	NA
RshSTT316	*Rhinolophus shameli*	Female	Nulliparous	Phnom Chhngauk	2023-05-05	31.84	30.75	Positive	Positive	OZ251014/OZ250935	BetaCoV-Sarbecovirus	OZ248238	NA
RshSTT317	*Rhinolophus shameli*	Female	Lactating	Phnom Chhngauk	2023-05-05	20.15	17.34	Positive	Positive	OZ251015/OZ250936	BetaCoV-Sarbecovirus	OZ248239	NA
RshSTT322	*Rhinolophus shameli*	Female	Nulliparous	Phnom Chhngauk	2023-05-05	21.32	19.14	Positive	Positive	OZ251016/OZ250937	BetaCoV-Sarbecovirus	OZ248240	NA
RshSTT327	*Rhinolophus shameli*	Female	Parous	Phnom Chhngauk	2023-05-05	33.37	31.43	Negative	Positive	OZ250890	BetaCoV-Sarbecovirus	OZ248241	NA
RshSTT328	*Rhinolophus shameli*	Female	Nulliparous	Phnom Chhngauk	2023-05-05	25.02	22.61	Positive	Positive	OZ251018/OZ250939	BetaCoV-Sarbecovirus	OZ248242	NA
RshSTT331	*Rhinolophus shameli*	Female	Nulliparous	Phnom Chhngauk	2023-05-05	25.41	23.4	Positive	Positive	OZ251019/OZ250940	BetaCoV-Sarbecovirus	OZ248243	NA
RacSTT333	*Rhinolophus acuminatus*	Female	Parous	Phnom Chhngauk	2023-05-05	29.07	28.72	Positive	Positive	OZ251020/OZ250941	BetaCoV-Sarbecovirus	OZ248244	NA
RacSTT334	*Rhinolophus acuminatus*	Female	Parous	Phnom Chhngauk	2023-05-05	32.12	31.42	Positive	Positive	OZ251021/OZ250942	BetaCoV-Sarbecovirus	OZ248179	OZ248101
RshSTT336	*Rhinolophus shameli*	Female	Parous	Phnom Chhngauk	2023-05-05	19.27	17.78	Positive	Positive	OZ251022/OZ250943	BetaCoV-Sarbecovirus	OZ248245	NA
RchSTT337	*Rhinolophus chaseni*	Female	Parous	Phnom Chhngauk	2023-05-05	39.58	38.37	Negative	Negative	NA	Sarbecovirus positive by RT-qPCR	OZ248246	NA
RshSTT344	*Rhinolophus shameli*	Female	Parous	Phnom Chhngauk	2023-05-05	19.05	17.33	Positive	Positive	OZ251023/OZ250944	BetaCoV-Sarbecovirus	OZ248247	NA
RacSTT345	*Rhinolophus acuminatus*	Female	Parous	Phnom Chhngauk	2023-05-05	17.68	15.52	Positive	Positive	OZ251024/OZ250945	BetaCoV-Sarbecovirus	OZ248180	OZ248102
RacSTT347	*Rhinolophus acuminatus*	Female	Parous	Phnom Chhngauk	2023-05-05	30.51	28.78	Positive	Positive	OZ251025/OZ250946	BetaCoV-Sarbecovirus	OZ248181	NA
RacSTT351	*Rhinolophus acuminatus*	Female	Parous	Phnom Chhngauk	2023-05-05	22.13	19.66	Positive	Positive	OZ251026/OZ250947	BetaCoV-Sarbecovirus	OZ248182	NA
RchSTT352	*Rhinolophus chaseni*	Female	Parous	Phnom Chhngauk	2023-05-05	38.19	37.25	Negative	Negative	NA	Sarbecovirus positive by RT-qPCR	OZ248183	NA
RpuSTT361	*Rhinolophus pusillus*	Female	Lactating	Phnom Ka Ngoak	2023-05-06	31.41	33.09	Positive	Positive	OZ251027/OZ250948	BetaCoV-Sarbecovirus	OZ248248	OZ248103
HarSTT369	*Hipposideros armiger*	Male	Juvenile	Phnom Ka Ngoak	2023-05-06	38.9	38.47	Negative	Negative	NA	Sarbecovirus positive by RT-qPCR	OZ248249	OZ248076
RshSTT381	*Rhinolophus shameli*	Male	Adult	Phnom Chhngauk	2023-06-12	23.66	20.39	Positive	Positive	OZ251028/OZ250949	BetaCoV-Sarbecovirus	OZ248250	NA
RshSTT395	*Rhinolophus shameli*	Female	Adult	Phnom Chhngauk	2023-06-12	24.85	23.64	Positive	Positive	OZ251032/OZ250953	BetaCoV-Sarbecovirus	OZ248251	NA
RshSTT419	*Rhinolophus shameli*	Male	Juvenile	Phnom Ka Ngoak	2023-06-13	19.33	18.45	Negative	Positive	OZ250892	BetaCoV-Sarbecovirus	OZ248252	NA
LlySTT440	*Lyroderma lyra*	Female	Adult	Phnom Ka Ngoak	2023-06-13	37.94	36.87	Negative	Negative	NA	BetaCoV-Sarbecovirus	OZ248184	OZ248087
RshSTT463	*Rhinolophus shameli*	Female	Adult	Phnom Chab Phleurng	2023-06-14	38.33	37.88	Negative	Negative	NA	Sarbecovirus positive by RT-qPCR	OZ248253	NA
RshSTT470	*Rhinolophus shameli*	Female	Adult	Phnom Chab Phleurng	2023-06-14	36.01	35.08	Negative	Positive	OZ250894	BetaCoV-Sarbecovirus	OZ248254	NA
RshSTT488	*Rhinolophus shameli*	Female	Adult	Phnom Chab Phleurng	2023-06-14	34.72	34.58	Positive	Negative	OZ250978	BetaCoV-Sarbecovirus	OZ248255	NA
RshSTT491	*Rhinolophus shameli*	Male	Adult	Phnom Chab Phleurng	2023-06-14	39.27	38.35	Negative	Negative	NA	Sarbecovirus positive by RT-qPCR	OZ248256	NA
RshSTT494	*Rhinolophus shameli*	Female	Adult	Phnom Chhngauk	2023-06-15	21.08	19.68	Positive	Positive	OZ251037/OZ250958	BetaCoV-Sarbecovirus	OZ248257	NA
RmaSTT500	*Rhinolophus malayanus*	Female	Adult	Phnom Chhngauk	2023-06-15	27.47	28.45	Positive	Positive	OZ251038/OZ250959	BetaCoV-Sarbecovirus	OZ248185	OZ248100
RshSTT502	*Rhinolophus shameli*	Male	Adult	Phnom Chhngauk	2023-06-15	26.51	24.89	Positive	Positive	OZ251039/OZ250960	BetaCoV-Sarbecovirus	OZ248258	NA
RshSTT503	*Rhinolophus shameli*	Male	Adult	Phnom Chhngauk	2023-06-15	34.86	34.27	Negative	Positive	OZ250896	BetaCoV-sarbecovirus & BetaCoV-Hibecovirus	OZ248259	NA
RshSTT511	*Rhinolophus shameli*	Male	Juvenile	Phnom Chhngauk	2023-06-15	28.01	25.92	Positive	Negative	OZ250979	BetaCoV-Sarbecovirus	OZ248260	NA
RshSTT512	*Rhinolophus shameli*	Female	Juvenile	Phnom Chhngauk	2023-06-15	25.53	23.66	Positive	Positive	OZ251042/OZ250963	BetaCoV-Sarbecovirus	OZ248261	NA
RshSTT515	*Rhinolophus shameli*	Female	Adult	Phnom Chhngauk	2023-06-15	18.65	17.22	Positive	Positive	OZ251043/OZ250964	BetaCoV-Sarbecovirus	OZ248262	NA
RshSTT516	*Rhinolophus shameli*	Female	Juvenile	Phnom Chhngauk	2023-06-15	33.8	31.44	Positive	Positive	OZ251044/OZ250965	BetaCoV-Sarbecovirus	OZ248263	NA
RshSTT517	*Rhinolophus shameli*	Female	Adult	Phnom Chhngauk	2023-06-15	35.69	35.21	Negative	Positive	OZ250897	BetaCoV-sarbecovirus & AlphaCoV-Decacovirus	OZ248264	NA
RshSTT518	*Rhinolophus shameli*	Female	Adult	Phnom Chhngauk	2023-06-15	30.93	29.43	Negative	Positive	OZ250898	BetaCoV-Sarbecovirus	OZ248265	NA
RmiSTT521	*Rhinolophus microglobosus*	Male	Adult	Phnom Chhngauk	2023-06-15	28.29	26.39	Positive	Negative	OZ250980	BetaCoV-sarbecovirus & AlphaCoV-Rhinacovirus	OZ248186	OZ248115
RshSTT523	*Rhinolophus shameli*	Male	Adult	Phnom Chhngauk	2023-06-15	38.18	39.38	Negative	Positive	OZ250900	BetaCoV-sarbecovirus & BetaCoV-Hibecovirus	OZ248266	NA
RshSTT524	*Rhinolophus shameli*	Male	Juvenile	Phnom Chhngauk	2023-06-15	26.55	24.8	Positive	Positive	OZ251045/OZ250966	BetaCoV-Sarbecovirus	OZ248267	NA
RshSTT562	*Rhinolophus shameli*	Male	Juvenile	Phnom Ka Ngoak	2023-06-16	25.17	24.02	Positive	Positive	OZ251047/OZ250968	BetaCoV-Sarbecovirus	OZ248268	NA
RshSTT564	*Rhinolophus shameli*	Male	Juvenile	Phnom Ka Ngoak	2023-06-16	21.01	19.27	Positive	Positive	OZ251048/OZ250969	BetaCoV-Sarbecovirus	OZ248269	NA
RshSTT565	*Rhinolophus shameli*	Male	Juvenile	Phnom Ka Ngoak	2023-06-16	29.49	29.04	Positive	Positive	OZ251049/OZ250970	BetaCoV-Sarbecovirus	OZ248270	NA
RshSTT570	*Rhinolophus shameli*	Male	Adult	Phnom Chab Phleurng	2023-06-17	21.55	20.28	Positive	Positive	OZ251050/OZ250971	BetaCoV-Sarbecovirus	OZ248271	NA
RshSTT575	*Rhinolophus shameli*	Female	Juvenile	Phnom Chab Phleurng	2023-06-17	33.17	32	Negative	Negative	NA	Sarbecovirus positive by RT-qPCR	OZ248187	NA
RshSTT576	*Rhinolophus shameli*	Female	Adult	Phnom Chab Phleurng	2023-06-17	27.39	26.43	Positive	Positive	OZ251052/OZ250973	BetaCoV-Sarbecovirus	OZ248272	NA
RshSTT586	*Rhinolophus shameli*	Female	Adult	Phnom Chab Phleurng	2023-06-17	26.36	25.3	Positive	Positive	OZ251053/OZ250974	BetaCoV-Sarbecovirus	OZ248273	NA
RshSTT587	*Rhinolophus shameli*	Female	Juvenile	Phnom Chab Phleurng	2023-06-17	33.47	32.74	Negative	Negative	NA	Sarbecovirus positive by RT-qPCR	OZ248188	NA
RshSTT590	*Rhinolophus shameli*	Male	Adult	Phnom Chab Phleurng	2023-06-17	25.15	22.28	Positive	Positive	OZ251054/OZ250975	BetaCoV-Sarbecovirus	OZ248274	NA
RshSTT599	*Rhinolophus shameli*	Female	Juvenile	Phnom Chab Phleurng	2023-06-17	39.58	39.27	Negative	Negative	NA	Sarbecovirus positive by RT-qPCR	OZ248189	NA
RshSTT610	*Rhinolophus shameli*	Female	Adult	Phnom Chab Phleurng	2023-06-17	18.36	16.45	Positive	Positive	OZ251056/OZ250977	BetaCoV-Sarbecovirus	OZ248275	NA
RshSTT612	*Rhinolophus shameli*	Male	Adult	Phnom Chab Phleurng	2023-06-17	40.49	38.15	Negative	Negative	NA	Sarbecovirus positive by RT-qPCR	OZ248276	NA
RshSTT010	*Rhinolophus shameli*	Female	Pregnant	Phnom Chhngauk	2023-03-27	Negative	Negative	Positive	Positive	OZ250982/OZ250903	AlphaCoV-Decacovirus	OZ248277	NA
RshSTT011	*Rhinolophus shameli*	Female	Pregnant	Phnom Chhngauk	2023-03-27	Negative	Negative	Positive	Positive	OZ250983/OZ250904	AlphaCoV-Decacovirus	OZ248278	NA
RshSTT012	*Rhinolophus shameli*	Female	Pregnant	Phnom Chhngauk	2023-03-27	Negative	Negative	Positive	Positive	OZ250984/OZ250905	AlphaCoV-Decacovirus	OZ248279	NA
RshSTT061	*Rhinolophus shameli*	Female	Pregnant	Phnom Ka Ngoak	2023-03-28	Negative	Negative	Positive	Positive	OZ250985/OZ250906	AlphaCoV-Decacovirus	OZ248280	NA
RshSTT067	*Rhinolophus shameli*	Female	Pregnant	Phnom Ka Ngoak	2023-03-28	Negative	Negative	Negative	Positive	OZ250880	AlphaCoV-Decacovirus	OZ248190	NA
RshSTT072	*Rhinolophus shameli*	Male	Adult	Phnom Ka Ngoak	2023-03-28	Negative	Negative	Positive	Positive	OZ250986/OZ250907	AlphaCoV-Decacovirus	OZ248281	NA
RshSTT105	*Rhinolophus shameli*	Female	Pregnant	Phnom Chab Phleurng	2023-03-29	Negative	Negative	Positive	Positive	OZ250987/OZ250908	AlphaCoV-Decacovirus	OZ248282	NA
RshSTT126	*Rhinolophus shameli*	Male	Adult	Phnom Chab Phleurng	2023-03-29	Negative	Negative	Positive	Positive	OZ250881	AlphaCoV-Decacovirus	OZ248283	NA
RshSTT188	*Rhinolophus shameli*	Female	Pregnant	Phnom Chhngauk	2023-03-30	Negative	Negative	Negative	Positive	OZ250882	AlphaCoV-Rhinacovirus	OZ248284	NA
HpoSTT192	*Hipposideros pomona*	Male	Adult	Phnom Chhngauk	2023-03-30	Negative	Negative	Positive	Positive	OZ250988/OZ250909	AlphaCoV-Decacovirus	OZ248285	NA
RshSTT193	*Rhinolophus shameli*	Female	Pregnant	Phnom Chhngauk	2023-03-30	Negative	Negative	Negative	Positive	OZ250883	AlphaCoV-Rhinacovirus	OZ248286	NA
RshSTT229	*Rhinolophus shameli*	Female	Parous	Phnom Chhngauk	2023-05-02	Negative	Negative	Positive	Positive	OZ250991/OZ250912	AlphaCoV-Decacovirus	OZ248287	NA
RshSTT276	*Rhinolophus shameli*	Male	Mature	Phnom Chab Phleurng	2023-05-04	Negative	Negative	Positive	Positive	OZ250999/OZ250920	AlphaCoV-Rhinacovirus	OZ248288	NA
RshSTT278	*Rhinolophus shameli*	Male	Mature	Phnom Chab Phleurng	2023-05-04	Negative	Negative	Positive	Positive	OZ251000/OZ250921	AlphaCoV-Decacovirus	OZ248289	NA
RshSTT289	*Rhinolophus shameli*	Female	Mature	Phnom Chab Phleurng	2023-05-04	Negative	Negative	Negative	Positive	OZ250886	AlphaCoV-Decacovirus	OZ248290	NA
RshSTT304	*Rhinolophus shameli*	Female	Nulliparous	Phnom Chab Phleurng	2023-05-04	Negative	Negative	Positive	Positive	OZ251009/OZ250930	AlphaCoV-Decacovirus	OZ248291	NA
RshSTT324	*Rhinolophus shameli*	Female	Nulliparous	Phnom Chhngauk	2023-05-05	Negative	Negative	Positive	Positive	OZ251017/OZ250938	AlphaCoV-Decacovirus	OZ248292	NA
RchSTT332	*Rhinolophus chaseni*	Female	Parous	Phnom Chhngauk	2023-05-05	Negative	Negative	Negative	Positive	OZ250891	AlphaCoV-Decacovirus	OZ248293	NA
HlaSTT383	*Hipposideros larvatus*	Male	Adult	Phnom Chhngauk	2023-06-12	Negative	Negative	Positive	Positive	OZ251029/OZ250950	BetaCoV-Hibecovirus	OZ248294	NA
HlaSTT384	*Hipposideros larvatus*	Female	Adult	Phnom Chhngauk	2023-06-12	Negative	Negative	Positive	Positive	OZ251030/OZ250951	BetaCoV-Hibecovirus	OZ248295	NA
HlaSTT390	*Hipposideros larvatus*	Female	Adult	Phnom Chhngauk	2023-06-12	Negative	Negative	Positive	Positive	OZ251031/OZ250952	BetaCoV-Hibecovirus	OZ248296	NA
HlaSTT416	*Hipposideros larvatus*	Female	Adult	Phnom Ka Ngoak	2023-06-13	Negative	Negative	Positive	Positive	OZ251033/OZ250954	BetaCoV-Hibecovirus	OZ248297	NA
HpoSTT420	*Hipposideros pomona*	Female	Adult	Phnom Ka Ngoak	2023-06-13	Negative	Negative	Positive	Positive	OZ251034/OZ250955	AlphaCoV-Decacovirus	OZ248298	NA
RshSTT433	*Rhinolophus shameli*	Male	Adult	Phnom Ka Ngoak	2023-06-13	Negative	Negative	Negative	Positive	OZ250893	AlphaCoV-Decacovirus	OZ248299	NA
RmiSTT466	*Rhinolophus microglobosus*	Male	Juvenile	Phnom Chab Phleurng	2023-06-14	Negative	Negative	Positive	Positive	OZ251035/OZ250956	AlphaCoV-Rhinacovirus	OZ248300	NA
RshSTT472	*Rhinolophus shameli*	Female	Adult	Phnom Chab Phleurng	2023-06-14	Negative	Negative	Negative	Positive	OZ250895	AlphaCoV-Decacovirus	OZ248301	NA
HlaSTT489	*Hipposideros larvatus*	Male	Adult	Phnom Chab Phleurng	2023-06-14	Negative	Negative	Positive	Positive	OZ251036/OZ250957	AlphaCoV-Decacovirus & BetaCoV-Hibecovirus	OZ248302	NA
HlaSTT507	*Hipposideros larvatus*	Female	Adult	Phnom Chhngauk	2023-06-15	Negative	Negative	Positive	Positive	OZ251040/OZ250961	AlphaCoV-Decacovirus	OZ248303	NA
HlaSTT508	*Hipposideros larvatus*	Female	Juvenile	Phnom Chhngauk	2023-06-15	Negative	Negative	Positive	Positive	OZ251041/OZ250962	AlphaCoV-Decacovirus & BetaCoV-Hibecovirus	OZ248304	NA
TmeSTT519	*Taphozous melanopogon*	Female	Adult	Phnom Chhngauk	2023-06-15	Negative	Negative	Negative	Positive	OZ250899	BetaCoV-Hibecovirus	OZ248191	NA
HdiSTT528	*Hipposideros diadema*	Male	Adult	Phnom Chhngauk	2023-06-15	Negative	Negative	Positive	Positive	OZ251046/OZ250967	BetaCoV-Hibecovirus	OZ248305	NA
MspSTT529	*Megaderma spasma*	Male	Adult	Phnom Ka Ngoak	2023-06-16	Negative	Negative	Negative	Positive	OZ250901	BetaCoV-Hibecovirus	OZ248306	NA
RshSTT561	*Rhinolophus shameli*	Male	Adult	Phnom Ka Ngoak	2023-06-16	Negative	Negative	Negative	Positive	OZ250902	AlphaCoV-Decacovirus	OZ248307	NA
HpoSTT572	*Hipposideros pomona*	Male	Adult	Phnom Chab Phleurng	2023-06-17	Negative	Negative	Positive	Positive	OZ251051/OZ250972	AlphaCoV-Decacovirus	OZ248308	NA
HlaSTT605	*Hipposideros larvatus*	Female	Adult	Phnom Chab Phleurng	2023-06-17	Negative	Negative	Positive	Positive	OZ251055/OZ250976	AlphaCoV-Decacovirus	OZ248309	NA
HpoSTT606	*Hipposideros pomona*	Male	Adult	Phnom Chab Phleurng	2023-06-17	Negative	Negative	Positive	Negative	OZ250981	AlphaCoV-Decacovirus	OZ248310	NA

NA: Not available

Supplementary Table 2. List of the 50 SARS-CoV-2 related virus positive bats samples processed for sequencing

Sample ID	Bat Genus/Species	Sampling date	Collection site	Sarbecovirus RT-qPCR (19)		Sequence length (nt)	Virus group	Sequencing method
				E-gene Ct value	N-gene Ct value			
RshSTT039	*Rhinolophus shameli*	2020-08-18	Phnom Chhngauk	29.22	27.33	29735	1	Untargeted, Nextera XT, Illumina and Ampliseq, ONT
RshSTT334	*Rhinolophus shameli*	2021-03-15	Phnom Chhngauk	24.35	23.5	29802	1	Untargeted, Nextera XT, Illumina
MspSTT571	*Megaderma Spasma*	2021-11-30	Phnom Chab Pleurng	38.4	38.27	5767 (partial)	1	Ampliseq, ONT
TmeSTT622	*Taphozous melanopogon*	2021-12-01	Phnom Ka Ngoak	37.99	36.38	11077 (partial)	1	Ampliseq, ONT
RshSTT671	*Rhinolophus shameli*	2021-12-02	Phnom Chhngauk	21.22	19.21	29694	1	Ampliseq, ONT
RshSTT779	*Rhinolophus shameli*	2021-12-06	Phnom Ka Ngoak	23.98	22.23	29694	1	Ampliseq, ONT
RshSTT783	*Rhinolophus shameli*	2021-12-06	Phnom Ka Ngoak	34.38	33.34	20907 (partial)	1	Ampliseq, ONT
RshSTT785	*Rhinolophus shameli*	2021-12-06	Phnom Ka Ngoak	40.63	36.77	10002 (partial)	1	Ampliseq, ONT
RshSTT814	*Rhinolophus shameli*	2021-12-06	Phnom Ka Ngoak	36.77	37.03	11415 (partial)	1	Ampliseq, ONT
RshSTT219	*Rhinolophus shameli*	2023-05-02	Phnom Chhngauk	24.29	24.02	29694	1	Ampliseq, ONT
RshSTT227	*Rhinolophus shameli*	2023-05-02	Phnom Chhngauk	23.29	23.17	29694	1	Ampliseq, ONT
RshSTT241	*Rhinolophus shameli*	2023-05-03	Phnom Ka Ngoak	28.08	27.05	29694	1	Ampliseq, ONT
RshSTT245	*Rhinolophus shameli*	2023-05-03	Phnom Ka Ngoak	17.05	16.86	29694	1	Ampliseq, ONT
RshSTT268	*Rhinolophus shameli*	2023-05-04	Phnom Chab Pleurng	21.46	21.12	29694	1	Ampliseq, ONT
RshSTT287	*Rhinolophus shameli*	2023-05-04	Phnom Chab Pleurng	23.5	21.45	29694	1	Ampliseq, ONT
RshSTT288	*Rhinolophus shameli*	2023-05-04	Phnom Chab Phleurng	37.12	36.22	Negative	NA	NA
RshSTT307	*Rhinolophus shameli*	2023-05-04	Phnom Chab Phleurng	36.13	33.92	1039 (partial)	Partial RdRp	Ampliseq, ONT
RshSTT313	*Rhinolophus shameli*	2023-05-05	Phnom Chhngauk	36.65	36.33	1033 (partial)	Partial RdRp	Ampliseq, ONT
RshSTT314	*Rhinolophus shameli*	2023-05-05	Phnom Chhngauk	20.87	18.09	29694	1	Ampliseq, ONT
RshSTT317	*Rhinolophus shameli*	2023-05-05	Phnom Chhngauk	20.15	17.34	29694	1	Ampliseq, ONT
RshSTT322	*Rhinolophus shameli*	2023-05-05	Phnom Chhngauk	21.32	19.14	29694	1	Ampliseq, ONT
RshSTT336	*Rhinolophus shameli*	2023-05-05	Phnom Chhngauk	19.27	17.78	29694	1	Ampliseq, ONT
RshSTT344	*Rhinolophus shameli*	2023-05-05	Phnom Chhngauk	19.05	17.33	29694	1	Ampliseq, ONT
RshSTT381	*Rhinolophus shameli*	2023-06-12	Phnom Chhngauk	23.66	20.39	29694	1	Ampliseq, ONT
RshSTT395	*Rhinolophus shameli*	2023-06-12	Phnom Chhngauk	24.85	23.64	29694	1	Ampliseq, ONT
RshSTT419	*Rhinolophus shameli*	2023-06-13	Phnom Ka Ngoak	19.33	18.45	29692	1	Ampliseq, ONT
RshSTT494	*Rhinolophus shameli*	2023-06-15	Phnom Chhngauk	21.08	19.68	29694	1	Ampliseq, ONT
RshSTT503	*Rhinolophus shameli*	2023-06-15	Phnom Chhngauk	34.86	34.27	Negative	NA	NA
RshSTT515	*Rhinolophus shameli*	2023-06-15	Phnom Chhngauk	18.65	17.22	29694	1	Ampliseq, ONT
RshSTT517	*Rhinolophus shameli*	2023-06-15	Phnom Chhngauk	35.69	35.21	Negative	NA	NA
RshSTT523	*Rhinolophus shameli*	2023-06-15	Phnom Chhngauk	38.18	39.38	Negative	NA	NA
RshSTT564	*Rhinolophus shameli*	2023-06-16	Phnom Ka Ngoak	21.01	19.27	29694	1	Ampliseq, ONT
RshSTT570	*Rhinolophus shameli*	2023-06-17	Phnom Chab Pleurng	21.55	20.28	29821	1	Untargeted, Nextera XT, Illumina
RshSTT610	*Rhinolophus shameli*	2023-06-17	Phnom Chab Pleurng	18.36	16.45	29694	1	Ampliseq, ONT
RmiSTT243	*Rhinolophus microglobosus*	2023-05-03	Phnom Ka Ngoak	17.86	15.76	29694	1	Ampliseq, ONT
RmiSTT249	*Rhinolophus microglobosus*	2023-05-03	Phnom Ka Ngoak	23.04	22.06	29694	1	Ampliseq, ONT
RmiSTT252	*Rhinolophus microglobosus*	2023-05-03	Phnom Ka Ngoak	39.58	37.47	Negative	NA	NA
RmiSTT521	*Rhinolophus microglobosus*	2023-06-15	Phnom Chhngauk	28.29	26.39	29694	1	Ampliseq, ONT
HlaSTT221	*Hipposideros larvatus*	2023-05-02	Phnom Chhngauk	39.57	38.74	8756 (partial)	1	Ampliseq, ONT
LlySTT440	*Lyroderma lyra*	2023-06-13	Phnom Ka Ngoak	37.94	36.87	16012 (partial)	1	Ampliseq, ONT
RchSTT337	*Rhinolophus chaseni*	2023-05-05	Phnom Chhngauk	37.05	38.37	Negative	NA	NA
RchSTT352	*Rhinolophus chaseni*	2023-05-05	Phnom Chhngauk	38.41	37.25	Negative	NA	NA
HarSTT369	*Hipposideros armiger*	2023-05-06	Phnom Ka Ngoak	38.47	38.9	Negative	NA	NA
RacSTT333	*Rhinolophus acuminatus*	2023-05-05	Phnom Chhngauk	29.07	28.72	29747	2	Untargeted, SISPA, ONT, and Ampliseq, ONT
RacSTT334	*Rhinolophus acuminatus*	2023-05-05	Phnom Chhngauk	32.12	31.42	29744	2	Untargeted, SISPA, ONT, and Ampliseq, ONT
RacSTT345	*Rhinolophus acuminatus*	2023-05-05	Phnom Chhngauk	17.68	15.52	29891	2	Untargeted, SISPA, ONT, and Untargeted, SMARTer, Illumina
RacSTT347	*Rhinolophus acuminatus*	2023-05-05	Phnom Chhngauk	30.51	28.78	29744	2	Untargeted, SISPA, ONT, and Ampliseq, ONT
RacSTT351	*Rhinolophus acuminatus*	2023-05-05	Phnom Chhngauk	22.13	19.66	29883	2	Untargeted, SISPA, ONT, and Untargeted, Nextera XT, Illumina
RpuSTT361	*Rhinolophus pusillus*	2023-05-06	Phnom Ka Ngoak	31.41	33.09	29750	3	Untargeted, SISPA, ONT, and Ampliseq, ONT
RmaSTT500	*Rhinolophus malayanus*	2023-06-15	Phnom Chhngauk	27.47	28.45	29642	4	Untargeted, SISPA, ONT, and Ampliseq, ONT

NA: Not available. RdRp: RNA-dependent RNA polymerase.

Untargeted, Nextera XT, Illumina: Metatranscriptomic sequencing following RNase H-based ribosomal RNA (rRNA) depletion, using the Nextera XT library preparation kit and the Illumina NextSeq system.

Untargeted, SISPA, ONT: Metatranscriptomic sequencing using the Sequence-Independent, Single-Primer Amplification (SISPA) technique and the ONT system.

Untargeted, SMARTer, Illumina: Metatranscriptomic sequencing using the SMARTer Stranded Total RNA-Seq kit and the Illumina NovaSeq system.

Ampliseq, ONT: PCR amplicon approach using a primer set optimized for full-genome coverage and the ONT system.

Supplementary Table 3. Repeated molecular testing of bat samples with low viral loads.

Bat genus	Sample ID	Bat species	Site of sample collection	Date of sampling	Sarbecovirus RT-qPCR (19)					
					1st extraction		2nd extraction		3rd extraction	
					E-gene Ct value	N-gene Ct value	E-gene Ct value	N-gene Ct value	E-gene Ct value	N-gene Ct value
Non-Rhinolophus	MspSTT571	*Megaderma spasma*	Phnom Chab Phleurng	2021-11-30	38.4	Negative	Negative	38.27	Negative	38.21
	TmeSTT622	*Taphozous melanopogon*	Phnom Ka Ngoak	2021-12-01	37.99	Negative	Negative	36.38	Negative	37.35
	HlaSTT221	*Hipposideros larvatus*	Phnom Chhngauk	2023-05-02	39.25	38.74	Negative	39.57	NA	NA
	HarSTT369	*Hipposideros armiger*	Phnom Ka Ngoak	2023-05-06	38.9	Negative	Negative	38.47	NA	NA
	LlySTT440	*Lyroderma lyra*	Phnom Ka Ngoak	2023-06-13	37.94	Negative	36.87	Negative	NA	NA
Rhinolophus	RshSTT783	*Rhinolophus shameli*	Phnom Ka Ngoak	2021-12-06	34.38	33.34	37.33	35.02	Negative	35
	RshSTT785	*Rhinolophus shameli*	Phnom Ka Ngoak	2021-12-06	40.63	36.77	36.15	37.68	Negative	38.05
	RshSTT814	*Rhinolophus shameli*	Phnom Ka Ngoak	2021-12-06	36.77	37.03	38.35	35.21	38.35	Negative
	RchSTT337	*Rhinolophus chaseni*	Phnom Chhngauk	2023-05-05	Negative	37.05	39.58	38.37	NA	NA
	RchSTT352	*Rhinolophus chaseni*	Phnom Chhngauk	2023-05-05	Negative	38.41	38.19	37.25	NA	NA

NA: Not available

Supplementary Table 4. Sequence identity of group 2 (RacSTT345) compared with SARS-CoV-2 and representative sarbecovirus genomes

	SARS-CoV-2		RaTG13		BANAL-20-236		RacCS203		Group3 (RpuSTT361)		Group4 (RmaSTT500)		Group1b (RshSTT570)	
	nt (%)	aa (%)	nt (%)	aa (%)	nt (%)	aa (%)	nt (%)	aa (%)	nt (%)	aa (%)	nt (%)	aa (%)	nt (%)	aa (%)
**Complete genome**	93.18		92.94		93.88		91.15		97.29		93.38		92.14	
**Orf 1ab**	94.26	97.32	94.03	96.94	94.07	97.10	94.07	96.79	96.33	97.87	96.26	97.72	93.47	96.01
**nsp1**	93.70	97.21	92.59	94.97	94.63	97.21	94.07	96.65	99.81	99.44	99.44	99.44	93.89	95.53
**nsp2**	92.22	95.60	92.58	94.98	92.27	94.51	94.72	95.45	98.07	97.49	98.12	97.02	88.30	91.05
**nsp3**	92.20	94.24	92.00	93.67	91.77	93.93	91.31	93.16	93.94	95.22	93.85	95.17	90.21	92.01
**nsp4**	92.53	97.60	92.67	97.60	92.53	97.60	90.67	96.79	93.07	96.79	93.27	97.19	92.53	96.99
**nsp5**	95.75	99.34	95.32	99.34	96.41	99.34	96.08	99.02	97.93	99.67	97.82	99.67	95.42	99.02
**nsp6**	97.70	99.65	95.98	100.00	98.28	99.65	97.93	99.31	98.85	99.31	98.74	98.96	98.28	98.62
**nsp7**	95.58	100.00	94.38	100.00	95.58	98.78	97.19	100.00	95.98	100.00	95.98	100.00	95.98	98.78
**nsp8**	97.98	99.49	96.80	99.49	96.63	98.98	98.15	98.98	97.81	99.49	97.98	99.49	98.15	99.49
**nsp9**	95.28	100.00	95.87	100.00	95.87	100.00	94.69	100.00	96.46	100.00	96.46	100.00	95.87	99.11
**nsp10**	96.16	99.28	95.68	99.28	95.92	99.28	95.20	99.28	98.80	99.28	98.32	99.28	97.60	98.55
**RdRp**	96.85	99.57	96.74	99.35	96.96	99.68	95.31	99.46	97.07	99.68	98.18	99.78	96.85	99.35
**nsp13**	96.56	99.17	96.28	99.17	95.95	99.00	95.90	98.83	98.17	99.50	97.17	98.83	95.90	99.00
**nsp14**	94.31	98.67	94.18	98.10	93.80	98.67	94.88	97.72	97.79	98.29	98.36	98.86	94.88	98.10
**nsp15**	93.55	97.97	93.64	96.81	93.45	97.68	98.17	99.13	98.46	99.42	98.55	99.42	97.88	99.13
**nsp16**	94.18	98.32	94.18	98.32	93.29	98.32	92.39	96.97	94.30	98.99	90.83	96.30	90.60	96.63
**S**	84.38	89.63	83.99	89.36	90.91	96.91	71.58	73.00	99.79	99.84	71.29	72.92	81.22	82.41
**NTD**	63.47	66.32	63.58	67.01	89.20	95.10	46.94	42.32	100.00	100.00	46.60	42.66	53.22	46.02
**RBD**	91.18	96.40	86.40	91.89	90.13	96.40	62.03	64.41	99.70	99.55	62.18	64.41	78.62	85.59
**RBM**	86.85	94.37	73.71	78.87	84.98	94.37	40.38	32.39	100.00	100.00	39.44	32.39	65.73	67.61
**ofr3a**	95.03	95.62	94.91	95.62	95.15	95.62	90.30	95.62	99.64	99.27	96.97	97.45	91.64	93.07
**E**	98.67	98.65	98.22	98.65	96.89	98.65	97.78	98.65	100.00	100.00	98.22	97.30	97.33	95.95
**M**	93.39	98.64	93.24	99.10	93.24	98.64	93.84	98.64	99.70	100.00	99.25	99.55	94.29	99.10
**orf6**	96.17	95.00	96.72	95.00	95.08	91.67	93.44	96.67	99.45	100.00	100.00	100.00	97.27	95.00
**orf7a**	96.41	98.33	95.58	97.50	96.69	99.17	93.09	95.83	99.72	99.17	98.34	98.33	95.58	95.00
**orf7b**	94.57	88.10	95.35	90.48	93.80	88.10	94.57	90.48	100.00	100.00	99.22	97.62	94.57	90.48
**orf8**	95.04	95.00	94.77	96.67	95.32	95.83	91.46	95.00	99.72	99.17	97.52	98.33	91.18	90.83
**N**	94.11	95.93	94.27	95.93	94.43	95.93	94.43	96.41	99.52	99.28	98.17	97.61	95.78	97.37
**orf10**	99.12	97.30	98.25	94.59	97.37	91.89	100.00	100.00	100.00	100.00	100.00	100.00	98.25	94.59

nt (%): Percent nucleotide identity, aa (%): Percent amino acid identity

Pairwise distances were calculated from the eight alignments of nucleotide and/or amino acid sequences of the complete genome and each gene using Geneious Prime v2025.0.3.

Supplementary Table 5. Codon sites presenting signal of selection in protein-coding genes of group 1 virus

Protein coding region	Model selection in EasyCodeML v1.41			Datamonkey (HyPhy)					
	Codon #	M8		Codon #	MEME	aBRSEL	SLAC	FEL	FUBAR
		dN/dS > 1	Bayes Empirical Bayes (BEB) > 0.95		P-value ≤ 0.1	P-value ≤ 0.05	P-value ≤ 0.1	P-value ≤ 0.1	Posterior probability ≥ 0.9
**ORF1ab**	123			123	**0.063**				
	211	**1.443**	0.934	211					**0.959**
	433			433	**0.096**				
	581	**1.318**	0.821	581					**0.915**
	797			797	**0.063**				
	971	**1.328**	0.829	971					
	1177			1177	**0.099**				
	1441			1441	**0.085**				
	1536			1536	**0.084**				
	2116	**1.329**	0.831	2116					
	3164			3164	**0.087**				
	3673	**1.33**	0.831	3673					**0.91**
	4470	**1.324**	0.826	4470					
	4540	**1.443**	0.935	4540					**0.963**
	5370	**1.336**	0.837	5370					
	6630	**1.319**	0.822	6630					**0.917**
	6651			6651	**0.059**				
	6709			6709	**0.069**				
	6957	**1.325**	0.827	6957	**0.048**				**0.907**
**S-gene recombinant region**	517	**1.245**	0.611	517					
	866			866	**0.091**				
	1051			1051	**0.035**				
	1103	**1.247**	0.652	1103					
**ORF3a**	169			169	**0.096**				

Supplementary Table 6. Primers and probes for coronaviruses molecular detection.

Name of assay	Oligos name	Oligos sequence (5'--> 3')	Gene targeted	Target virus family noted by the authors
Chu et al RT-PCR (21)	Chu1-Forward	GGKTGGGAYTAYCCKAARTG	RdRp gene	Pan-coronaviruses
	Chu1-Reverse	TGYTGTSWRCARAAYTCRTG		
	Chu2-Forward	GGTTGGGACTATCCTAAGTGTGA		
	Chu2-Reverse	CCATCATCAGATAGAATCATCAT		
Quan et al RT-PCR (20)	CoV-FWD1	CGTTGGIACWAAYBTVCCWYTICARBTRGG	RdRp gene	Pan-coronaviruses
	CoV-RVS1	GGTCATKATAGCRTCAVMASWWGCNACATG		
	CoV-FWD2	GGCWCCWCCHGGNGARCAATT		
	CoV-RVS2	GGWAWCCCCAYTGYTGWAYRTC		
Corman et al RT-qPCR (19)	E_Sarbeco_F	ACAGGTACGTTAATAGTTAATAGCGT	E gene	Sarbecoviruses
	E_Sarbeco_P1	FAM-ACACTAGCCATCCTTACTGCGCTTCG-BBQ		
	E_Sarbeco_R	ATATTGCAGCAGTACGCACACA		
	N_Sarbeco_F	CACATTGGCACCCGCAATC	N gene	
	N_Sarbeco_P	FAM-ACTTCCTCAAGGAACAACATTGCCA-BBQ		
	N_Sarbeco_R	GAGGAACGAGAAGAGGCTTG		

Supplementary Table 7. List of publicly available sequences used in this study

Sequence name	Country	Species	Accession number (GenBank or GISAID)	Collection date
BANAL-20-103	Laos	Bat-*R_pusillus*	MZ937001	2020-07-07
BANAL-20-116	Laos	Bat-*R_malayanus*	MZ937002	2020-07-07
BANAL-20-236	Laos	Bat-*R_marshalli*	MZ937003	2020-07-10
BANAL-20-247	Laos	Bat-*R_malayanus*	MZ937004	2020-07-10
BANAL-20-52	Laos	Bat-*R_malayanus*	MZ937000	2020-07-05
CoVZC45	China-Zhoushan-Dinghai	Bat-*R_sinicus*	MG772933	2017-02
CoVZXC21	China-Zhoushan-Dinghai	Bat-*R_sinicus*	MG772934	2015-07
HN2021A	China-Hunan	Bat-*R_pusillus*	OK017803	2021-02
HN2021B	China-Hunan	Bat-*R_pusillus*	OK017804	2021-02
HN2021G	China-Hunan	Bat-*R_pusillus*	OK017805	2021-03
PrC31	China-Yunnan	Bat-*R_pusillus*	MW703458	2018-08
Ra22QT106	Vietnam-Quang Tri	Bat-*R_affinis*	OR233322	2022-11-03
Ra22QT135	Vietnam-Quang Tri	Bat-*R_affinis*	OR233323	2022-11-04
Ra22QT137	Vietnam-Quang Tri	Bat-*R_affinis*	OR233328	2022-11-04
Ra22QT77	Vietnam-Quang Tri	Bat-*R_affinis*	OR233324	2022-11-03
RacCS203	Thailand-Chachoengsao	Bat-*R_acuminatus*	MW251308	2020-06-19
RaTG13	China-Yunnan	Bat-*R_affinis*	MN996532	2013-07-24
RmYN02	China-Yunnan	Bat-*R_malayanus*	EPI_ISL_412977	2019-06-25
Rp_JCC9	China-Yunnan	Bat-*R_blythi*	OK287355	2020-03
Rp22DB159	Vietnam-Dien Bien	Bat-*R_pusillus*	OR233302	2022-06-26
RpYN06	China-Yunnan	Bat-*R_pusillus*	MZ081381	2020-05-25
Wuhan-Hu-1	China-Wuhan	Human	NC_045512	2019-12
YN2021	China-Yunnan	Bat-*R_pusillus*	OK017806	2021-04
R24	China-Yunnan	Bat-*R_marshalli*	OP963576	2018
G1	China-Guangdong	Pangolin	EPI_ISL_410721	2019
MP789	China-Guangdong	Pangolin	MT121216	2019-03-29
P1E	China-Guangxi	Pangolin	MT040334	2017
P2V	China-Guangxi	Pangolin	MT072864	2018
P4L	China-Guangxi	Pangolin	MT040333	2017
P5E	China-Guangxi	Pangolin	MT040336	2017
P5L	China-Guangxi	Pangolin	MT040335	2017
P79-9	China-Guangdong	Pangolin	OQ297708	2019-03
RshSTT182	Cambodia-Steung Treng	Bat-*R_shameli*	EPI_ISL_852604	2010-12-06
RshSTT200	Cambodia-Steung Treng	Bat-*R_shameli*	EPI_ISL_852605	2010-12-06
GZ02*	China-Guangzhou	Human	AY390556	2002

R= Rhinolophus

*This sequence was only included in the analysis presented in Figure 3.

Supplementary Table 8. Evaluation of temporal signal and clock models comparisons

Dataset	Clock model	Log marginal likelihood with sampling dates	Log marginal likelihood without sampling dates
R1	SC	-14918	-14970
	UCLN	-14894	-14918
R2	SC	-9854	-9893
	UCLN	-9824	-9843
R3	SC	-13024	-13052
	UCLN	-12955	-12977
R4	SC	-19341	-19377
	UCLN	-19043	-19061
R5	SC	-6512	-6575
	UCLN	-6483	-6517
R6	SC	-10615	-10664
	UCLN	-10588	-10606
R7	SC	-6566	-6594
	UCLN	-6565	-6596
R8	SC	-9051	-9097
	UCLN	-9025	-9052
R9	SC	-10172	-10176
	UCLN	-10152	-10171
R10	SC	-8577	-8623
	UCLN	-8540	-8550
R11	SC	-6229	-6273
	UCLN	-6205	-6234
R12	SC	-13997	-14022
	UCLN	-13827	-13846
R13	SC	-6366	-6382
	UCLN	-6349	-6362
NRA	SC	-95382	-95468
	UCLN	-95364	-95388
Non recombinant region group 1	SC	-43407	-43493
	UCLN	-43403	-43424
Non recombinant region group 1a	SC	-40830	-40889
	UCLN	-40830	-40866

SC: Strict clock, UCLN: uncorrelated relaxed clock with lognormal distribution.

Log-marginal likelihood estimates using the generalized stepping stone sampling (GSS) model selection approach are presented for dataset using the sample dates, and the same dataset analyzed without dates, resulting in positive Bayes factors and indicating that there was temporal signal in the data.

Supplementary Table 9. Coalescent tree priors comparisons

Dataset		Coalescent tree prior Log marginal likelihood				
		Constant size	Exponential growth	Bayesian skygrid	Bayesian skyline	Bayesian skyride
R1	PS	-14851	-14848	-14810	**-14807**	-14842
	SS	-14852	-14849	-14811	**-14807**	-14842
R2	PS	-9810	-9801	-9762	**-9760**	-9800
	SS	-9811	-9801	-9763	**-9761**	-9800
R3	PS	-12910	-12909	-12875	**-12872**	-12904
	SS	-12911	-12910	-12877	**-12873**	-12904
R4	PS	-18925	-18920	-18884	**-18880**	-18908
	SS	-18926	-18921	-18885	**-18881**	-18908
R5	PS	-6464	-6461	-6443	**-6440**	-6468
	SS	-6465	-6462	-6443	**-6441**	-6468
R6	PS	-10545	-10544	-10502	**-10493**	-10541
	SS	-10546	-10545	-10503	**-10494**	-10541
R7	PS	-6536	-6531	-6492	**-6487**	-6537
	SS	-6537	-6532	-6493	**-6488**	-6537
R8	PS	-9007	-9004	-8969	**-8963**	-9006
	SS	-9008	-9005	-8969	**-8964**	-9006
R9	PS	-10140	-10139	-10094	**-10089**	-10141
	SS	-10141	-10140	-10094	**-10091**	-10141
R10	PS	-8525	-8524	-8492	**-8489**	-8528
	SS	-8526	-8525	-8493	**-8490**	-8528
R11	PS	-6196	-6193	**-6158**	-6161	-6191
	SS	-6197	-6193	**-6158**	-6161	-6191
R12	PS	-13774	-13772	-13732	**-13726**	-13752
	SS	-13775	-13772	-13733	**-13726**	-13752
R13	PS	-6340	-6337	-6305	**-6304**	-6329
	SS	-6340	-6337	-6305	**-6304**	-6329
NRA	PS	-94937	-94930	-94893	**-94881**	-94931
	SS	-94938	-94931	-94895	**-94880**	-94930
Non recombinant region group 1	PS	**-43360**	**-43360**	**-43360**	**-43360**	-43366
	SS	**-43360**	**-43360**	**-43360**	**-43360**	-43366
Non recombinant region group 1a	PS	-40821	-40822	**-40820**	-40826	-40822
	SS	-40821	-40822	**-40820**	-40826	-40822

The log marginal likelihood values were estimated using both path sampling (PS) and stepping-stone sampling (SS), and the values highlighted in bold indicate the best supported model from log Bayes factor comparisons.
