# Supplementary material for: Characterization and evolutionary history of novel SARS-CoV-2-related viruses in bats from Cambodia
Source: Nat Commun. 2026 Jul 23;17:9002. doi: 10.1038/s41467-026-75954-1 (PMC13507159; doi:10.1038/s41467-026-75954-1)
Supplement: Supplementary file 2 [file 41467_2026_75954_MOESM2_ESM.pdf]

nature portfolio

Corresponding author(s): Veasna DUONG and Etienne SIMON-LORIERE

Last updated by author(s): May 26, 2025

Reporting Summary

Nature Portfolio wishes to improve the reproducibility of the work that we publish. This form provides structure for consistency and transparency in reporting. For further information on Nature Portfolio policies, see our Editorial Policies and the Editorial Policy Checklist.

Statistics

For all statistical analyses, confirm that the following items are present in the figure legend, table legend, main text, or Methods section.

n/a	Confirmed	
☐	☒	The exact sample size (*n*) for each experimental group/condition, given as a discrete number and unit of measurement
☒	☐	A statement on whether measurements were taken from distinct samples or whether the same sample was measured repeatedly
☐	☒	The statistical test(s) used AND whether they are one- or two-sided<br>*Only common tests should be described solely by name; describe more complex techniques in the Methods section.*
☒	☐	A description of all covariates tested
☒	☐	A description of any assumptions or corrections, such as tests of normality and adjustment for multiple comparisons
☐	☒	A full description of the statistical parameters including central tendency (e.g. means) or other basic estimates (e.g. regression coefficient) AND variation (e.g. standard deviation) or associated estimates of uncertainty (e.g. confidence intervals)
☐	☒	For null hypothesis testing, the test statistic (e.g. *F*, *t*, *r*) with confidence intervals, effect sizes, degrees of freedom and *P* value noted<br>*Give P values as exact values whenever suitable.*
☐	☒	For Bayesian analysis, information on the choice of priors and Markov chain Monte Carlo settings
☒	☐	For hierarchical and complex designs, identification of the appropriate level for tests and full reporting of outcomes
☒	☐	Estimates of effect sizes (e.g. Cohen's *d*, Pearson's *r*), indicating how they were calculated

Our web collection on statistics for biologists contains articles on many of the points above.

Software and code

Policy information about availability of computer code

Data collection	Reference genome sequence data were downloaded from GenBank and GISAID via their web interfaces.
Data analysis	Software used: MAFFT v7.508, Trimmomatic v0.39, SPAdes v3.14.0, MEGAHIT v1.2.9, canu v2.2, DIAMOND v2.0.4, CLC Assembly Cell v5.1.0, minimap2 v2.17-r941, IGV v2.12.3, iVar v1.3.1, seqtk v1.3-r106, IQ-TREE v2.2.0, RDP4 v4.97, Geneious Prime 2023.0.1, ARTIC pipeline, TempEst v.1.5.3, BEAST v.1.10.4, Tracer v.1.7.1, Tree Annotator v.1.10.4, FigTree v.1.4.7, DATAMONKEY (web), EasyCODEML (Java), SWISS-MODEL (web).

For manuscripts utilizing custom algorithms or software that are central to the research but not yet described in published literature, software must be made available to editors and reviewers. We strongly encourage code deposition in a community repository (e.g. GitHub). See the Nature Portfolio guidelines for submitting code & software for further information.

Data

Policy information about availability of data

All manuscripts must include a data availability statement. This statement should provide the following information, where applicable:

- Accession codes, unique identifiers, or web links for publicly available datasets
- A description of any restrictions on data availability
- For clinical datasets or third party data, please ensure that the statement adheres to our policy

Raw sequence data (from targeted or untargeted sequencing) were deposited on the NCBI Sequence Reads Archive (SRA) under BioProject PRJNA1267097 (https://www.ncbi.nlm.nih.gov/bioproject/PRJNA1267097).

Assembled viral genomes are also available on the GISAID EpiCoV database with ID EPI_SET_250414kp (https://doi.org/10.55876/gis8.250414kp) and on the GenBank database with accession numbers PZ487726-PZ487758.
The COI and ACE2 sequences were deposited on the European Nucleotide Archive (ENA) under BioProject PRJEB87055.
Partial (targeted) RdRp sequences were deposited on the ENA under BioProject PRJEB86850.
Primers designed in this study are available at https://github.com/Simon-LoriereLab/BatCoVCambodia2020-23 and on https://doi.org/10.5281/zenodo.20629491.
Source data are provided with this paper.

Research involving human participants, their data, or biological material

Policy information about studies with human participants or human data. See also policy information about sex, gender (identity/presentation), and sexual orientation and race, ethnicity and racism.

Reporting on sex and gender	This study did not include human participants, their data, or biological material.
Reporting on race, ethnicity, or other socially relevant groupings	This study did not include human participants, their data, or biological material.
Population characteristics	This study did not include human participants, their data, or biological material.
Recruitment	This study did not include human participants, their data, or biological material.
Ethics oversight	This study did not include human participants, their data, or biological material.

Note that full information on the approval of the study protocol must also be provided in the manuscript.

Field-specific reporting

Please select the one below that is the best fit for your research. If you are not sure, read the appropriate sections before making your selection.

☒ Life sciences ☐ Behavioural & social sciences ☐ Ecological, evolutionary & environmental sciences

For a reference copy of the document with all sections, see nature.com/documents/nr-reporting-summary-flat.pdf

Life sciences study design

All studies must disclose on these points even when the disclosure is negative.

Sample size	We screened 1,462 rectal swab samples collected from nine bat genera during eight field missions between August 2020 and June 2023. Of the 1,462 rectal swab samples tested, 146 were positive by at least one method: 23 exclusively by sarbecoviruses RT-qPCR, 56 by pan-coronaviruses RT-PCRs, and 67 by both sarbecoviruses RT-qPCR and pan-coronaviruses RT-PCRs. Sanger sequencing was performed on 123 samples that tested positive with at least one of the pan-coronaviruses RT-PCRs targeting distinct parts of the RNA-dependent RNA polymerase gene. We used metatranscriptomic and amplicon-based approaches to generate sequence data from a selection of 50 bat samples that tested positive for sarbecovirus by RT-qPCR, out of a total of 90 (23+67) positive bat samples.
Data exclusions	No data were excluded. Only complete or near complete genomes were kept for the phylodynamic analyses.
Replication	Sequencing of the spike was performed independently for confirmation.<br>To investigate a sample presenting numerous intrahost single nucleotide variants (iSNVs), an independent RNA extraction and sequencing run was performed.
Randomization	Randomization was not relevant for this study as assays were read in an unbiased manner - i.e. there was little or no interpretation involved that could be influenced by unconscious bias.
Blinding	Randomization was not relevant for this study as assays were read in an unbiased manner - i.e. there was little or no interpretation involved that could be influenced by unconscious bias.

Reporting for specific materials, systems and methods

We require information from authors about some types of materials, experimental systems and methods used in many studies. Here, indicate whether each material, system or method listed is relevant to your study. If you are not sure if a list item applies to your research, read the appropriate section before selecting a response.

Materials & experimental systems

n/a	Involved in the study	
x		Antibodies
	x	Eukaryotic cell lines
x		Palaeontology and archaeology
	x	Animals and other organisms
x		Clinical data
x		Dual use research of concern
x		Plants

Methods

n/a	Involved in the study	
x		ChIP-seq
x		Flow cytometry
x		MRI-based neuroimaging

Eukaryotic cell lines

Policy information about cell lines and Sex and Gender in Research

Cell line source(s)	HEK293T and Vero cells were purchased from Sigma. The establishment of the Rhileki cell line is described in Mah MG et al, Microbiol Spectr. 2023 Jun 15;11(3):e0348322. doi: 10.1128/spectrum.03483-22. PMID: 37199653.
Authentication	The purchase was recent for the HEK293T and Vero cells, and we did not perform re-authentication of the cell lines.
Mycoplasma contamination	All 3 cell lines tested negative for mycoplasma.
Commonly misidentified lines (See ICLAC register)	No commonly misidentified cell lines were used in the study.

Animals and other research organisms

Policy information about studies involving animals; ARRIVE guidelines recommended for reporting animal research, and Sex and Gender in Research

Laboratory animals	No laboratory animals were involved in this study.
Wild animals	Eight sampling sessions were conducted on the following dates: August 18-22, 2020; October 12-17, 2020, March 13-17, 2021; August 30-September 3, 2021; November 29-December 7, 2021; March 27-30, 2023; May 2-6, 2023 and June 12-17, 2023. Each mission spanned consecutive nights across four different karst hill locations (Phnom Chab Phleurng, Phnom Chhngauk, Phnom Ka Ngoak, and Phnom Kork Romeas) and each hill was investigated on two non-consecutive nights. Mist nets were set up each evening, either at the entrance of caves or on potential bat flyways and monitored during a consistent 2-hour period per night, before dusk and continued until 8:00 pm each. Capture, handling, species identification, sampling and release of live bats followed established standard procedures used by the study group and adhered to international standards for animal research. Captured bats were immediately placed in individual cloth bags until being processed. The following biological samples were collected from captured bats: blood, oral swab, rectal swab, and wing punch. Swabs were individually stored in TRIzol™ reagent (Sigma-Aldrich) and viral transport medium (VTM; containing tryptose phosphate Broth 2.95%, 145 mM of NaCl, 5% gelatin, 54 mM Amphotericin B, 106 U of penicillin-streptomycine per liter, 80 mg of gentamycine per liter [Sigma-Aldrich, Steinheim, Germany]).
Reporting on sex	Our study did not include only one sex. All captured bats were examined for species identification based on morphological criteria, sex determination, age and reproductive status.
Field-collected samples	All field activities were conducted in accordance with appropriate biosafety standards. Personnel wore suitable personal protective equipment, and strict decontamination procedures were applied to all materials and equipment used during sampling and transport. All samples were stored on ice during collection and subsequently in liquid nitrogen in the field, and subsequently transferred to the Institut Pasteur du Cambodge biobank, where they were stored at -80°C. Downstream processing was conducted under biosafety level 3 (BSL-3) conditions.
Ethics oversight	The study followed all relevant institutional and national guidelines for animal care and use. A study agreement was obtained from the Forestry Administration who participated in the field investigations and oversaw all aspects of the study for active sampling, as no animal ethics committee exists in Cambodia. All bat captures, handling and sampling were conducted with the participation of officers of the Forestry Administration of Cambodia under a MoU signed between Institut Pasteur du Cambodge and the Ministry of Agriculture, Forestry and Fisheries. Animal capture and handling were in accordance with the guidelines approved by the American Society of Mammalogists, in addition to the requirements of the statutory study permission provided by the national authority responsible for wildlife research, i.e.: the Forestry Administration of the Cambodian Ministry of Agriculture, Forest and Fisheries. The experimental protocol was approved by the Ethical Committee of VetAgro-Sup in Lyon, France (Comité d'Ethique de VetAgroSup n∘18, Avis 2301).

Note that full information on the approval of the study protocol must also be provided in the manuscript.

Plants

Seed stocks	*Report on the source of all seed stocks or other plant material used. If applicable, state the seed stock centre and catalogue number. If plant specimens were collected from the field, describe the collection location, date and sampling procedures.*
Novel plant genotypes	*Describe the methods by which all novel plant genotypes were produced. This includes those generated by transgenic approaches, gene editing, chemical/radiation-based mutagenesis and hybridization. For transgenic lines, describe the transformation method, the number of independent lines analyzed and the generation upon which experiments were performed. For gene-edited lines, describe the editor used, the endogenous sequence targeted for editing, the targeting guide RNA sequence (if applicable) and how the editor was applied.*
Authentication	*Describe any authentication procedures for each seed stock used or novel genotype generated. Describe any experiments used to assess the effect of a mutation and, where applicable, how potential secondary effects (e.g. second site T-DNA insertions, mosiacism, off-target gene editing) were examined.*
