# Supplementary material for: Characterization and evolutionary history of novel SARS-CoV-2-related viruses in bats from Cambodia
Source: Nat Commun. 2026 Jul 23;17:9002. doi: 10.1038/s41467-026-75954-1 (PMC13507159; doi:10.1038/s41467-026-75954-1)
Supplement: Supplementary file 3 [file 41467_2026_75954_MOESM3_ESM.pdf]

nature portfolio

Peer Review File

Characterization and evolutionary history of novel SARS-CoV-2-related viruses in bats from Cambodia

Corresponding Author: Dr Etienne SIMON-LORIERE

This file contains all reviewer reports in order by version, followed by all author rebuttals in order by version.

Version 0:

Reviewer comments:

Reviewer #1

(Remarks to the Author)
In this manuscript, Ou et al., reported discovery and characterization of groups of novel sarbecoviruses phylogenetically related to SARS-CoV-2 from bats in Cambodia. The findings of this study have enriched the data about genetic diversity of bat sarbecoviruses, and have provided valuable information for understanding the spatial-temporal evolution of bat sarbecoviruses in Southeast Asia, which is known as hotspot for sarbecovirus circulation and evolution. Through pseudovirus assays, their results suggest the spillover risk of one group of newly identified bat sarbecoviruses from Cambodia, which is important for strategy of prevention and early warning of future emergence of sarbecoviruses in this region. However, major revision is needed before the manuscript can be considered for publication.

Major points:

1. The authors demonstrated that the spike of Group 2 Cambodian bat sarbecoviruses, represented by RacSTT345, is able to mediate the entry into cells expressing human ACE2 and bat ACE2. The bat ACE2 tested here is from Rhinolophus shameli (Rsh). However, the natural host species of this group of virus is Rhinolophus acuminatus (Rac). Hence the utilization of RacACE2 also needs to be tested and presented. In addition, for RacSTT345, it is strongly recommended that the authors can compare its usage of ACE2 from more host species, and perform a testing of cell tropism using cell lines from a diversity of mammalian species and tissues, to better evaluate its cross-species transmission risk and identify potential intermediate hosts.

2. According to the phylogenetic trees, the group 2 viruses (RacSTT345 and others) formed novel clades in several genomic regions. To more directly illustrate the similarity/distinction between these viruses and previously known sarbecoviruses, the authors need do a Simplot analysis using the genome sequences, and provide a supplementary table showing their sequence identity for full-length genome and in different genes (ORFs).

3. As this study is based on a longitudinal surveillance, the authors should compare and discuss the variation of prevalence rate as well as virus loading of bat sarbecoviruses in Cambodia in different sampling months or seasons. Such information is important for long-term sarbecovirus surveillance in the future.

4. In the figure 3, it is suggested that a phylogenetic tree of RBM of the sarbecoviruses selected for RBM alignment should be added (in a panel on the left of the alignment). The phylogenetic tree in parallel to the sequence alignment can more clearly reflect the grouping and RBM genotyping of different sarbecoviruses.

Minor points:

1. Although the group 2 viruses from Cambodia bats can use hACE2, the virus isolation was unsuccessful. The author should discuss the possible reason for the failure of virus isolation.

2. Line 34: “33 complete sarbecovirus genome” should be “33 complete sarbecovirus genome sequences”.

3. The detection of sarbecovirus in non-Rhinolophus bat samples such as Taphozous are probably due to contamination. The authors can repeat the PCR testing of these samples can confirm whether it is false positive.

4. In supplementary figure 10, the authors provide a map of the sampling locations showing the distribution of the newly detected bat sarbecoviruses in Cambodia. As the manuscript discusses the evolutionary relationship between the Cambodian strains and the strains from other SEA countries such as Laos, Thailand and Vietnam, the origin location of those viruses shall also be labeled in an extended map of Indo-chine peninsula.

Reviewer #2

(Remarks to the Author)
Ou et al. provide evidence for several novel SARS-CoV-2 related bat coronavirus in Cambodia which they identified through extensive, multi-year surveillance work and a combination of genomic approaches. They further investigate the genetic diversity and evolution of these bat coronaviruses via phylogenetic and recombination analysis. The findings provide valuable insights into bat-coronavirus diversity and ecology, a topic that remains of high scientific (and political) interest since the closest viral sequence relateded to SARS-CoV-2 viruses were isolated from swabs collected from bats in Southern China. This work further adds evidence to a series of studies that various currently circulating BatCoVs are potentially able to use human ACE2 as cell entry receptor. This represents an additional geographical location and independent report on the diversity, mutational and recombinatorial capacity of these viruses, supporting continuation of similar surveillance and research work. In summary, the manuscript contributes to our understanding of the prevalence and ecology of BatCoVs which is crucial for informing further research as well as pandemic preparedness planning. Sequences, genomes and molecular tools (amplicon primer shemes) presented here can further aid and inform future studies. Thus, the manuscript is well suited for publication in Nature Communications after incorporating some minor revisions as listed below.
Minor Issues:
• Line 75: for context, it would be helpful to give some more details / show a map where the province is located within the country and the region? also see comment below
• Line 93: diversity (of?) alpha- and betacoronaviruses
• Lines 95 onwards: hard to read this paragraph with only the sample names - might help to explain the sample naming abbreviations / numbers are chronological, related to a date / species?, it seems there is no figure associated to this paragraph, although it contains a lot of important information, I feel like a combination of Supplemental figure 10 and number of samples / % positives per site could be helpful
• Line 143: RDP4 - wasn’t previously introduced as Recombination Detection Program
• Line 211 onwards: how does this range compare to the analysis in Pekar et al 2025, Cell (The recency and geographical origins of the bat viruses ancestral to SARS-CoV and SARS-CoV-2)?
• Line 242: How do these results compare to the expectations based on the modelling in the previous paragraph?
• Line 345 onwards: How were the sampling sessions established – do they correspond to any factors like rainfall / breeding activity etc?
• Line 345 onwards: were there any individuals recaptured? This could be informative to estimate how much % of the overall population the sampling did reach (assuming would be quite low but given the longitudinal study and longevity of bats, recaptures might occur?)
• Line 345 onwards: adding the buffer in which swabs were stored and transported might be useful for the readers
• Line 364 onward: It might be good to elaborate on why these specific PCR primers were selected and list them in a table, which target, which viral family and the source?
• Line 389: which kits / probes were used for rRNA depletion? Human-based or bat-specific?
• Line 381 onwards: an overview which sequencing method was used on how many samples and which was deemed the best would be helpful
• Line 578: paragraph missing?
Anne Hahn

Reviewer #3

(Remarks to the Author)
Putita Ou et al. present a targeted sampling, sequencing, and phylodynamic study of SARS-CoV-2-related viruses in bats from Cambodia. Through extensive sampling and virus screening, they identified novel SARS-CoV-2-related lineages, and they subsequently characterized these viruses and their relationships to known sarbecoviruses. Several of these exhibit high similarity to SARS-CoV-2, and lab work suggested moderate zoonotic risk from at least one lineage. Overall, the study is well-conceived and methodologically sound, and the new data contribute to our understanding of sarbecovirus diversity in Southeast Asia. I only have minor suggestions and requests for clarification, outlined below.

Some of the sequences (e.g., RmiSTT521) were unable to be obtained with both PCRs, as seen in Fig. S1; this should explicitly be noted in the text.

Please clarify what is meant by “late” Ct values and what Ct threshold(s) are being referred to.

The authors state that the constellation of mutations in RshSTT564 corresponding to the minor variant population was distinct from all other consensus sequences generated in this work. What about subsets of the constellations? Were any observed in other sequences? And how did these mutations compare to other iSNVs observed among the other samples? A more thorough examination here is warranted to ensure that cross-contamination did not occur.

How was the genome set(s) for RDP4 and the subsequent phylodynamics chosen? Is it constrained to the sequences in

Supplementary Table S3?

There were fewer breakpoints than observed in other studies on recombination (e.g., Lytras et al., 2022 [GBE] and Pekar et al., 2025 [Cell]). Can you contextualize your results relative to theirs? Were there any matching breakpoints, particularly in known “recombination hotspots”? The inference of fewer breakpoints could result in potentially false evolutionary histories of some of these viruses, that are ultimately aggregates of multiple evolutionary histories. Although there likely was not substantial recombination missed among the Cambodian samples themselves, if a breakpoint(s) was missed among other closely related but geographically distant lineages (e.g., the samples in Vietnam or Thailand), the phylogeographic inferences and interpretations could be biased.

There are other tools in the Datamonkey suite that are often used to determine site-specific selection, namely MEME, that could be helpful. Additionally, have the authors considered examining branch-specific selection, such as with aBRSEL?

While the current functional assays appropriately address the authors’ primary goals, the manuscript could be further strengthened by expanding the panel of spike proteins and ACE2 orthologs tested. A broader “round-robin”–style assay—including additional viruses and host receptors—would not be necessary for the central conclusions but could provide valuable insight into sarbecovirus host range and cross-species tropism.

Lines 189–190, the authors discuss that the substitution rates may be affected by the short time range and could reflect “incomplete purifying selection processes”. Such potential selection processes are ongoing, rather than something incomplete; consider rephrasing. These statements also allude to the lack of observation of the time dependent rate phenomenon, which should be noted in the text, if that is indeed what the authors are suggesting. Importantly, we do not yet have a good understanding of the rate of evolution of sarbecoviruses more broadly, with substantial differences across the sarbecovirus phylogeny. This additional context would be helpful for the reader.

Lines 313–315, the authors mention there could be host adaptation through codon usage optimization. Although true, the synonymous mutations could also reflect either differing evolutionary rates or, more simply, neutral evolution. Additional qualifications here would strengthen the discussion.

Lines 460–462: the phrasing “sarbecoviruses not sampled in Cambodia also presented evidence of recombination but were kept in these alignments as they were not the focus of the analyses” (emphasis mine) is slightly confusing. Were the sequences included or excluded from the final dataset, and, if they were excluded, why? Please clarify.

Line 496: Please state how many rate categories for the GTR+I+G model?

Supplementary Fig. 3: This figure would benefit from an in-figure legend.

Can the authors provide more detail about ACE2 variation in R. shameli and related bats in the discussion? This would elucidate the potential host range of these viruses and further strengthen the discussion.

Version 1:

Reviewer comments:

Reviewer #1

(Remarks to the Author)
Authors have greatly imporved the manuscript following the suggestions from the reviewers. This reviewer has no more suggestions.

Reviewer #3

(Remarks to the Author)
The authors have broadly addressed my concerns. I have only a few remaining minor comments.

Supplementary Figure 9: Can you please change this to a log axis? This would be helpful to the reader in understanding rate differences.

Line 198: Please include the 95% HPD for the evolutionary rate estimate.

Line 204: The time dependent rate phenomena would not be particular to a lineage in this case—it would likely be present throughout the phylogeny.

Data and code availability: the breakpoints, alignments (except for restricted data), and phylogenies should be made available. Additionally, it would be helpful to have tables of the relevant tMRCAs across the different regions of the genome.

Open Access This Peer Review File is licensed under a Creative Commons Attribution 4.0 International License, which permits use, sharing, adaptation, distribution and reproduction in any medium or format, as long as you give appropriate credit to the original author(s) and the source, provide a link to the Creative Commons license, and indicate if changes were made.
In cases where reviewers are anonymous, credit should be given to 'Anonymous Referee' and the source.
The images or other third party material in this Peer Review File are included in the article's Creative Commons license, unless indicated otherwise in a credit line to the material. If material is not included in the article's Creative Commons license and your intended use is not permitted by statutory regulation or exceeds the permitted use, you will need to obtain permission directly from the copyright holder.
To view a copy of this license, visit https://creativecommons.org/licenses/by/4.0/

REVIEWER COMMENTS

Reviewer #1 (Remarks to the Author):

In this manuscript, Ou et al., reported discovery and characterization of groups of novel sarbecoviruses phylogenetically related to SARS-CoV-2 from bats in Cambodia. The findings of this study have enriched the data about genetic diversity of bat sarbecoviruses, and have provided valuable information for understanding the spatial-temporal evolution of bat sarbecoviruses in Southeast Asia, which is known as hotspot for sarbecovirus circulation and evolution. Through pseudovirus assays, their results suggest the spillover risk of one group of newly identified bat sarbecoviruses from Cambodia, which is important for strategy of prevention and early warning of future emergence of sarbecoviruses in this region. However, major revision is needed before the manuscript can be considered for publication.

We thank the reviewer for this positive analysis, and for the points raised and the revisions suggested.

Major points:

1. The authors demonstrated that the spike of Group 2 Cambodian bat sarbecoviruses, represented by RacSTT345, is able to mediate the entry into cells expressing human ACE2 and bat ACE2. The bat ACE2 tested here is from Rhinolophus shameli (Rsh). However, the natural host species of this group of virus is Rhinolophus acuminatus (Rac). Hence the utilization of RacACE2 also needs to be tested and presented. In addition, for RacSTT345, it is strongly recommended that the authors can compare its usage of ACE2 from more host species, and perform a testing of cell tropism using cell lines from a diversity of mammalian species and tissues, to better evaluate its cross-species transmission risk and identify potential intermediate hosts.
The reviewer raised a very relevant point, which we had not addressed due to resources constraints. Thanks to a new collaboration, we were able to obtain plasmids coding for the ACE2 of different species, including R. acuminatus. This allowed us to test the capacity of the group 2 spike to mediate entry into cells expressing these ACE2 versions, confirming that this spike is compatible with diverse bat and mammalian species, similar to what has been shown for SARS-CoV-2 spike (new results page 6 and revised Fig. 4).

2. According to the phylogenetic trees, the group 2 viruses (RacSTT345 and others) formed novel clades in several genomic regions. To more directly illustrate the similarity/distinction between these viruses and previously known sarbecoviruses, the authors need do a Simplot analysis using the genome sequences, and provide a supplementary table showing their sequence identity for full-length genome and in different genes (ORFs).
Following the suggestion of the reviewer, we have generated a SimPlot showing the similarity of group 2 viruses to a selection of known sarbecoviruses (Supplementary Fig. 6), and an identity table (Supplementary Table 4). These additional data are presented page 4.

3. As this study is based on a longitudinal surveillance, the authors should compare and discuss the variation of prevalence rate as well as virus loading of bat sarbecoviruses in Cambodia in different sampling months or seasons. Such information is important for long-term sarbecovirus surveillance in the future.
We thank the reviewer for this legitimate suggestion. We also appreciate the importance of better understanding seasonal and temporal variations in the prevalence rates and viral load

of bat sarbecoviruses. However, we could not perform this comparison within the scope of the current study:
The samples analyzed here were collected as part of three different projects, each with its own collection schedule and protocol. There were gaps between these projects due to variability in funding availability and implementation timelines. The dataset presented here unfortunately does not support an accurate analysis of seasonal or temporal variations in prevalence rates. We have now better explained these limitations in the method section. We hope that such an analysis on variation of prevalence rate and on viral loads will be possible in the future thanks to ongoing efforts in Cambodia.

4. In the figure 3, it is suggested that a phylogenetic tree of RBM of the sarbecoviruses selected for RBM alignment should be added (in a panel on the left of the alignment). The phylogenetic tree in parallel to the sequence alignment can more clearly reflect the grouping and RBM genotyping of different sarbecoviruses.
We thank the reviewer for this suggestion. We have added the tree in Fig. 3 panel b, and grouped the sequences of the alignment accordingly. We used a cladogram visualization to fit the suggested tree as the divergence between some taxa led to both very short and long branches.

Minor points:

1. Although the group 2 viruses from Cambodia bats can use hACE2, the virus isolation was unsuccessful. The author should discuss the possible reason for the failure of virus isolation.
We now discuss the possible reasons for this failure in the result section (page 4).

2. Line 34: “33 complete sarbecovirus genome” should be “33 complete sarbecovirus genome sequences”.
We have added the missing word.

3. The detection of sarbecovirus in non-Rhinolophus bat samples such as Taphozous are probably due to contamination. The authors can repeat the PCR testing of these samples can confirm whether it is false positive.
Thank you for this relevant question. While we acknowledge the possibility of contamination, we believe that the detection of sarbecovirus in non-Rhinolophus bat samples like Taphozous is of significant interest to the scientific community. To enhance the rigor of our findings, we have conducted multiple separate RNA extractions for each sample with high Ct values (>37), performed on distinct days, in different rooms, and by different experimenters. These results, now detailed in Supplementary Table 3, show that RT-qPCR results were consistently positive across all extracted products for at least one of the two targets, indicating the presence of low amounts of viral RNA. This consistency across multiple extractions and tests provides solid evidence against laboratory contamination. While we have taken utmost care during sampling, we cannot entirely rule out field contamination. We hope that our findings will lead to new collections, allowing to further validate these observations.

4. In supplementary figure 10, the authors provide a map of the sampling locations showing the distribution of the newly detected bat sarbecoviruses in Cambodia. As the manuscript discusses the evolutionary relationship between the Cambodian strains and the strains from other SEA countries such as Laos, Thailand and Vietnam, the origin location of those viruses shall also be labeled in an extended map of Indo-chine peninsula.

We thank the reviewer for this suggestion. We have revised Supplementary Fig. 10 (now

renumbered to Supplementary Fig. 1) to present an extended map recapitulating the sampling location of the publicly available viral genomes used in this study.

Reviewer #2 (Remarks to the Author):

Ou et al. provide evidence for several novel SARS-CoV-2 related bat coronavirus in Cambodia which they identified through extensive, multi-year surveillance work and a combination of genomic approaches. They further investigate the genetic diversity and evolution of these bat coronaviruses via phylogenetic and recombination analysis. The findings provide valuable insights into bat-coronavirus diversity and ecology, a topic that remains of high scientific (and political) interest since the closest viral sequence relateded to SARS-CoV-2 viruses were isolated from swabs collected from bats in Southern China. This work further adds evidence to a series of studies that various currently circulating BatCoVs are potentially able to use human ACE2 as cell entry receptor. This represents an additional geographical location and independent report on the diversity, mutational and recombinatorial capacity of these viruses, supporting continuation of similar surveillance and research work. In summary, the manuscript contributes to our understanding of the prevalence and ecology of BatCoVs which is crucial for informing further research as well as pandemic preparedness planning. Sequences, genomes and molecular tools (amplicon primer shemes) presented here can further aid and inform future studies. Thus, the manuscript is well suited for publication in Nature Communications after incorporating some minor revisions as listed below.

We thank the reviewer for this positive review, and for the revisions suggested.

Minor Issues:
• Line 75: for context, it would be helpful to give some more details / show a map where the province is located within the country and the region? also see comment below
We have generated a new version of Supplementary Fig. 10 (now Supplementary Fig. 1) to highlight the sampling sites in the context of the country and the region.

• Line 93: diversity (of?) alpha- and betacoronaviruses
We have added the missing word.

• Lines 95 onwards: hard to read this paragraph with only the sample names - might help to explain the sample naming abbreviations / numbers are chronological, related to a date / species?, it seems there is no figure associated to this paragraph, although it contains a lot of important information, I feel like a combination of Supplemental figure 10 and number of samples / % positives per site could be helpful
We have added a sentence to describe the samples coding, cited the Supplementary Table 1 where the metadata (location, sampling date, tests results) are detailed, and described the symbols used to highlight these samples in the trees shown in Supplementary Fig. 2 (previously Supplementary Fig. 1, renumbered). We also added the number of samples / % positives per site into Supplementary Fig. S10 (renumbered as Supplementary Fig. 1).

• Line 143: RDP4 - wasn't previously introduced as Recombination Detection Program
We have now introduced RDP and added the corresponding citation.

• Line 211 onwards: how does this range compare to the analysis in Pekar et al 2025, Cell (The recency and geographical origins of the bat viruses ancestral to SARS-CoV and SARS-CoV-2)?

This comparison of course has limitations as the data sets used, non-recombinant regions selected, and methodology differ, as well as the specific focus (closest ancestor to SARS-CoV-1 or 2 compared to our analysis focused on the closest ancestor to our newly discovered SARS-CoV-2-like viruses). However, we could compare - for non-recombinant regions that could be compared - our substitution rate estimates. Our estimates ranges overlap those reported in Pekar et al, for the "SARS-CoV-2-like viruses" with the "prior using late 2020 SARS-CoV-2 genomes" (95% highest posterior density ranging from 1.08E-04 to 1.92E-03 subst./site/year). We have added this citation in this section (page 5)

• Line 242: How do these results compare to the expectations based on the modelling in the previous paragraph?
For the group 2/3 spike, the results are in line with the expectation based on the modeling. We have now edited the text to mention this (page 7). For the group 1 spike (not able to mediate entry into cells expressing hACE2 in our pseudovirus system), the results are also compatible with the homology modeling analysis, likely reflecting the variations in residues described as key contact for hACE2, or the shorter loop at the beginning of the RBM. However, as we noted in the text, a publication using another pseudovirus system (VSV based) reported a (weak) capacity to mediate entry of a group 1 spike into cells expressing hACE2.

• Line 345 onwards: How were the sampling sessions established – do they correspond to any factors like rainfall / breeding activity etc?
The samples analyzed here were collected as part of three different projects, each with its own collection schedule and protocol. The field missions were planned to be distributed throughout the year to sample bats in the rainy and dry season, with the hope of identifying periods corresponding to peaks of births (increased sampling of juveniles). We hope that a comprehensive analysis factors influencing the prevalence rate will be possible in the future thanks to ongoing efforts in Cambodia.

• Line 345 onwards: were there any individuals recaptured? This could be informative to estimate how much % of the overall population the sampling did reach (assuming would be quite low but given the longitudinal study and longevity of bats, recaptures might occur?)
The field teams did not recapture any bat during these missions. We do not know whether this is due to chance (we do not know the scale of the bat populations in this area, but it is likely very big), if the captured bats learned to avoid capture, or if despite the care taken during the sampling procedures, the sampled bats all died before the following sampling session (hopefully not the case).

• Line 345 onwards: adding the buffer in which swabs were stored and transported might be useful for the readers
We present our apologies for the lack of precision; we have now completed the Methods section to specify the buffers used (page 10).

• Line 364 onward: It might be good to elaborate on why these specific PCR primers were selected and list them in a table, which target, which viral family and the source?
We now present the molecular detection assays in a table (Supplementary Table 6), and explain why these assays were selected.

• Line 389: which kits / probes were used for rRNA depletion? Human-based or bat-specific?
We present our apologies for the lack of precision; we have now specified the use of custom DNA probes corresponding to human rRNAs.

• Line 381 onwards: an overview which sequencing method was used on how many samples and which was deemed the best would be helpful
We have added an overview of the approaches used, and explained why the combination (we must say also taking costs into account) seemed the most reasonable and efficient.

• Line 578: paragraph missing?
We have clarified that no custom code was developed for this project.

Anne Hahn

Reviewer #3 (Remarks to the Author):

Putita Ou et al. present a targeted sampling, sequencing, and phylodynamic study of SARS-CoV-2-related viruses in bats from Cambodia. Through extensive sampling and virus screening, they identified novel SARS-CoV-2-related lineages, and they subsequently characterized these viruses and their relationships to known sarbecoviruses. Several of these exhibit high similarity to SARS-CoV-2, and lab work suggested moderate zoonotic risk from at least one lineage. Overall, the study is well-conceived and methodologically sound, and the new data contribute to our understanding of sarbecovirus diversity in Southeast Asia. I only have minor suggestions and requests for clarification, outlined below.

We thank the reviewer for this positive evaluation, and for the suggestions.

Some of the sequences (e.g., RmiSTT521) were unable to be obtained with both PCRs, as seen in Fig. S1; this should explicitly be noted in the text.
It is indeed difficult to track the information about sequences from Supplementary Fig. 2 (renumbered, was Fig. S1) as there are hundreds of them. We have now added a symbol (*) to highlight samples for which the Sanger sequencing of one of the 2 PCR products failed, and another symbol (**) for those where the included sequence was obtained from high-throughput sequencing attempts.

Please clarify what is meant by “late” Ct values and what Ct threshold(s) are being referred to.
We used a Ct value of 32 as a threshold for “late” Ct value, and have specified this in the revised text.

The authors state that the constellation of mutations in RshSTT564 corresponding to the minor variant population was distinct from all other consensus sequences generated in this work. What about subsets of the constellations? Were any observed in other sequences? And how did these mutations compare to other iSNVs observed among the other samples? A more thorough examination here is warranted to ensure that cross-contamination did not occur.

We had concerns similar to those of the reviewer. Among the 112 iSNVs observed at a frequency of ~0.23 [0.21 to 0.25], 26 changes, distributed along the genome (visualization below), were unique compared to the other genomes of our dataset, including in the related subset (32 sequences of group 1 viruses, Figure below). This spread of unique variations along the genome gave us confidence that this sample correspond to a co-infection with a closely related virus.

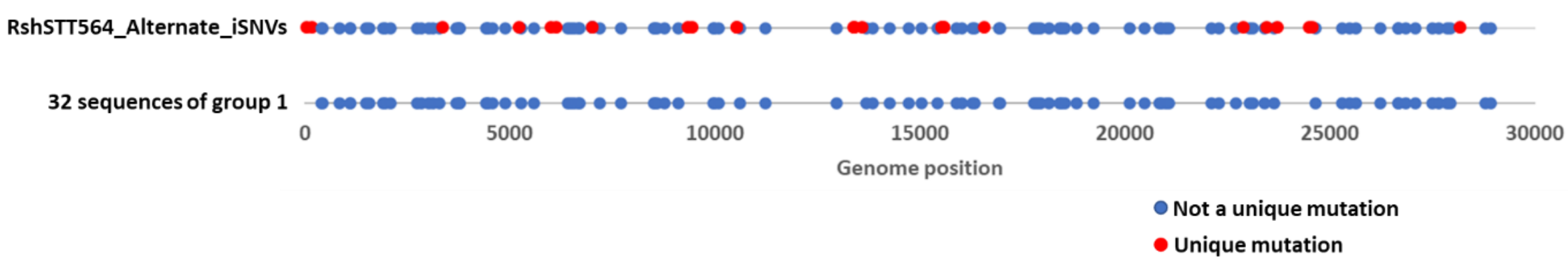

How was the genome set(s) for RDP4 and the subsequent phylodynamics chosen? Is it constrained to the sequences in Supplementary Table S3?
The genomes set for recombination and subsequent analyses were chosen to include the publicly available/known (published work) diversity of the SARS-CoV-2-related viruses among the sarbecoviruses. The analysis was indeed constrained to sequences listed in Supplementary Table 7 (renumbered, was previously Supplementary Table 3) (after also excluding partial genomes).

There were fewer breakpoints than observed in other studies on recombination (e.g., Lytras et al., 2022 [GBE] and Pekar et al., 2025 [Cell]). Can you contextualize your results relative to theirs? Were there any matching breakpoints, particularly in known “recombination hotspots”? The inference of fewer breakpoints could result in potentially false evolutionary histories of some of these viruses, that are ultimately aggregates of multiple evolutionary histories. Although there likely was not substantial recombination missed among the Cambodian samples themselves, if a breakpoint(s) was missed among other closely related but geographically distant lineages (e.g., the samples in Vietnam or Thailand), the phylogeographic inferences and interpretations could be biased.
Indeed, our analysis was focused on the novel viral genomes identified in Cambodia and their close relatives, and is thus not systematic as the studies cited, that used larger datasets, including notably SARS-CoV-1 lineage genomes. Interestingly, comparisons with these studies indicate that most of our regions’ boundaries match breakpoints detected in these larger datasets, including some defined as hotspots, but most often, our 13 regions are further divided into smaller regions, which is expected. Some exceptions correspond to the spike and the end of the genome when compared to breakpoints reported for SARS-CoV-2-like viruses in Pekar et al., 2025 [Cell], suggesting cold spots (due to recombination itself, or to selection for functional viruses) in this lineage.
As we considered performing phylodynamic and phylogeographic analyses on the novel viruses using different regions of the genomes, we were very mindful of the point raised by the reviewer on the risk of bias. Thus, we have not attempted to draw conclusions that could be biased by unsampled data or missed recombination breakpoints.

There are other tools in the Datamonkey suite that are often used to determine site-specific selection, namely MEME, that could be helpful. Additionally, have the authors considered examining branch-specific selection, such as with aBRSEL?
We thank the reviewer for this suggestion. We have added the results of the suggested MEME and aBRSEL analysis and compiled all the results into a new Supplementary Table 5. We note in the text that one site (position 6957 within ORF1ab in our alignment), detected by two methods, was close to statistical significance.

While the current functional assays appropriately address the authors’ primary goals, the manuscript could be further strengthened by expanding the panel of spike proteins and ACE2 orthologs tested. A broader “round-robin”–style assay—including additional viruses and host receptors—would not be necessary for the central conclusions but could provide valuable insight into sarbecovirus host range and cross-species tropism.

We agree with this relevant comment of the reviewer, also noted by reviewer 1. Thanks to a new collaboration, we were able to obtain plasmids coding for the ACE2 of different species, including *R. acuminatus*. Focusing on the novel group 2 spike, we were able to test its capacity to mediate entry into cells expressing additional ACE2 versions, confirming that this spike is compatible with the sampled host, and with other mammalian species ACE2, similar to what has been shown for SARS-CoV-2 spike (new results page 6 and revised Fig 4).

Lines 189–190, the authors discuss that the substitution rates may be affected by the short time range and could reflect "incomplete purifying selection processes". Such potential selection processes are ongoing, rather than something incomplete; consider rephrasing. These statements also allude to the lack of observation of the time dependent rate phenomenon, which should be noted in the text, if that is indeed what the authors are suggesting. Importantly, we do not yet have a good understanding of the rate of evolution of sarbecoviruses more broadly, with substantial differences across the sarbecovirus phylogeny. This additional context would be helpful for the reader.
We thank the reviewer for pushing us to improve this section. We have rephrased the sentence and added context with a new citation (page 5).

Lines 313–315, the authors mention there could be host adaptation through codon usage optimization. Although true, the synonymous mutations could also reflect either differing evolutionary rates or, more simply, neutral evolution. Additional qualifications here would strengthen the discussion.
We agree with the reviewer that we were too narrow in this discussion point. We have expanded the list of factors that could contribute to the observed differences as suggested (page 8).

Lines 460–462: the phrasing "sarbecoviruses not sampled in Cambodia also presented evidence of recombination but were kept in these alignments as they were not the focus of the analyses" (emphasis mine) is slightly confusing. Were the sequences included or excluded from the final dataset, and, if they were excluded, why? Please clarify.

We apologize for the lack of clarity. We confirm that these sequences were included in the final dataset, with the rationale that they would not affect inferences made on the newly discovered viruses from Cambodia. We have edited the text to clarify the inclusion of these genomes (page 13).

Line 496: Please state how many rate categories for the GTR+I+G model?
We have added this information to the text.

Supplementary Fig. 3: This figure would benefit from an in-figure legend.
We have added an in-figure legend.

Can the authors provide more detail about ACE2 variation in R. shameli and related bats in the discussion? This would elucidate the potential host range of these viruses and further strengthen the discussion.
Phylogenetic analysis (Supplementary Figure 10, renumbered, was previously Supplementary Figure 8) and pairwise comparisons of ACE2 sequences showed 99.99–100% nucleotide identity among *R. shameli* bats, with no amino acid variations. Compared to other bat species, nucleotide identities were 99% with *R. microglobosus* and *R. affinis*, and 97% with *R. acuminatus*, *R. pusillus*, and *R. malayanus*. We have also generated an alignment showing the variations at positions identified as proximity or contact residues

between hACE2 and SARS-CoV-2 (as a model for the interaction), and present this in a new panel of Supplementary Figure 10. We note both conserved and variable sites among bat species related to those sampled in Cambodia.

REVIEWER COMMENTS

Reviewer #1 (Remarks to the Author):

Authors have greatly imporved the manuscript following the suggestions from the reviewers. This reviewer has no more suggestions.

We thank the reviewer again for his time and expertise.

Reviewer #3 (Remarks to the Author):
The authors have broadly addressed my concerns. I have only a few remaining minor comments.

We thank the reviewer again for his time and expertise.

Supplementary Figure 9: Can you please change this to a log axis? This would be helpful to the reader in understanding rate differences.

We have changed the axis to help the readers, and also added a value we were already citing in the main text.

Line 198: Please include the 95% HPD for the evolutionary rate estimate.

We have added the 95% HPDs.

Line 204: The time dependent rate phenomena would not be particular to a lineage in this case—it would likely be present throughout the phylogeny.

We have corrected this sentence.

Data and code availability: the breakpoints, alignments (except for restricted data), and phylogenies should be made available. Additionally, it would be helpful to have tables of the relevant tMRCAs across the different regions of the genome.

We have added the data to the GitHub repository, for which we obtained a permanent identifier at Zenodo: https://doi.org/10.5281/zenodo.20395167
